# The backbone of the post-synaptic density originated in a unicellular ancestor of choanoflagellates and metazoans

**DOI:** 10.1186/1471-2148-10-34

**Published:** 2010-02-03

**Authors:** Alexandre Alié, Michaël Manuel

**Affiliations:** 1 UPMC Univ Paris 06, UMR 7138 Systématique, Adaptation, Evolution CNRS IRD MNHN, Bâtiment A, 4ème étage, Case 05, Université Pierre et Marie Curie, 7 quai St Bernard, 75 005 Paris, France

## Abstract

**Background:**

Comparative genomics of the early diverging metazoan lineages and of their unicellular sister-groups opens new window to reconstructing the genetic changes which preceded or accompanied the evolution of multicellular body plans. A recent analysis found that the genome of the nerve-less sponges encodes the homologues of most vertebrate post-synaptic proteins. In vertebrate excitatory synapses, these proteins assemble to form the post-synaptic density, a complex molecular platform linking membrane receptors, components of their signalling pathways, and the cytoskeleton. Newly available genomes from *Monosiga brevicollis *(a member of Choanoflagellata, the closest unicellular relatives of animals) and *Trichoplax adhaerens *(a member of Placozoa: besides sponges, the only nerve-less metazoans) offer an opportunity to refine our understanding of post-synaptic protein evolution.

**Results:**

Searches for orthologous proteins and reconstruction of gene gains/losses based on the taxon phylogeny indicate that post-synaptic proteins originated in two main steps. The backbone scaffold proteins (Shank, Homer, DLG) and some of their partners were acquired in a unicellular ancestor of choanoflagellates and metazoans. A substantial additional set appeared in an exclusive ancestor of the Metazoa. The placozoan genome contains most post-synaptic genes but lacks some of them. Notably, the master-scaffold protein Shank might have been lost secondarily in the placozoan lineage.

**Conclusions:**

The time of origination of most post-synaptic proteins was not concomitant with the acquisition of synapses or neural-like cells. The backbone of the scaffold emerged in a unicellular context and was probably not involved in cell-cell communication. Based on the reconstructed protein composition and potential interactions, its ancestral function could have been to link calcium signalling and cytoskeleton regulation. The complex later became integrated into the evolving synapse through the addition of novel functionalities.

## Background

Many of the complex traits that distinguish the organisation and biology of multicellular animals originated during the early phase of their evolutionary history. Among these, the emergence of the neuro-sensory system represents a particularly fascinating conundrum, because the biological support of animal perception and behaviour is extraordinarily complex at the molecular, cellular and anatomical scales, and without equivalent in the living world. A promising path to understanding the genetic changes that shaped the body plan of multicellular animals has been recently opened by comparative analyses of genomes from the early-diverging animal lineages and the closest unicellular relatives of metazoans (e.g. [1-10])

The recent characterisation of a nearly complete set of post-synaptic protein orthologues in the genome of the sponge *Amphimedon queenslandica *[2] represents a successful illustration of this approach. Excitatory synapses in the vertebrate central nervous system are characterised by an electron-dense structure called the post-synaptic density (PSD) below the post-synaptic membrane. Proteomic investigations have identified about one thousand proteins in this complex [11-14]. Some of these proteins (e.g. Shank, DLG, Homer, GRIP) have pivotal roles in building a scaffold for the anchorage of the other PSD components, for instance membrane receptors for neurotransmitters, cation channels, members of intracellular signalling pathways, and cytoskeleton or cytoskeleton regulation proteins [15-18]. This complex sub-membrane plateform thereby connects cell surface receptors with components of signal transduction pathways and with the cytoskeleton machinery. The PSD is also involved in synaptogenesis, dendritic spine formation and synaptic plasticity [17,19,20]. The three-dimensional architecture of the PSD is determined by multiple protein-protein interactions through binding domains (e.g. [17,18,21]). For example, the vertebrate Shank protein, the master scaffold protein of the PSD [18], contains five interacting domains (from N-ter to C-ter: ankyrin repeats, SH3 domain, PDZ domain, proline-rich region, SAM domain) involved in a huge variety of homomeric and heteromeric interactions. Among PSD scaffold proteins, PDZ (for Post-synaptic density protein 95/Drosophila disc large tumor suppressor/Zo-1) is the most common type of binding domain, with many proteins containing multiple PDZ domains (e.g. three in the DLG protein; seven in GRIP).

Not only were most PSD proteins identified in the sponge genome (whereas most are absent in fungal or plant genomes), but moreover these proteins essentially contain the same interacting domains arranged into the same architectures as in their vertebrate counterparts [2]. Intriguingly, several of the sponge PSD-like genes (*DLG, GKAP, GRIP, HOMER, CRIPT*) were found co-expressed in a particular non-ciliated epidermal cell type of the *Amphimedon *larva, the globular cell (erroneously called "flask cells" in [2], see [22]). These observations are consistent with the existence in sponges of an assembled structure similar to the vertebrate PSD, despite absence of recognisable nerve cells and synapses.

The PSD protein complex would therefore have originated before the acquisition of synapses in a nerve-less ancestor, at least under the conventional view that sponges branched off earlier than animals with a nervous system in the phylogeny (e.g. [23-27]). Moreover, since the sponge globular cells express Notch/Delta homologues and a proneural-like bHLH transcription factor [22] in addition to PSD genes, they might represent putative sensory "proto-neural" cells responding to environmental stimuli [22] and a possible "starting point" for the evolution of neural cells [2]. Some recent molecular phylogenies [28-31] have pointed to more complicated evolutionary scenarios (e.g. secondary loss of nerve cells and synapses in sponges) by challenging the monophyly of animals with a nervous system (Cnidaria, Ctenophora and Bilateria, classically grouped under the Eumetazoa) and the early divergence of sponges (discussed in [32,33]). However, a recent phylogenomic study with increased species sampling of non-bilaterian lineages and sophisticated model of sequence evolution [34] lent support to the conventional phylogeny and thereby to a single acquisition of nerve cells and synapses in a eumetazoan ancestor.

Since the publication of [2], additional annotated complete genomes have become available. The present study aims at improving our understanding of the emergence of the PSD protein complex by focusing on sequence data from *Monosiga brevicollis *and *Trichoplax adhaerens*, two organisms of particular interest with respect to early metazoan evolution. *Monosiga *is a member of choanoflagellates, the unicellular sister-group of the Metazoa [1,8]. Previous studies have suggested (without rigorous analyses) the existence of some PSD protein orthologues in the *Monosiga *genome [2], but these have been considered "very few"[10], supporting the idea that the PSD scaffold is essentially an innovation of the metazoan lineage. However, this remains to be tested rigorously through systematic analyses of choanoflagellate orthologues of the post-synaptic genes. *Trichoplax *(phylum Placozoa) is a nerve-less animal whose flat and asymmetric body comprises two epithelial cell layers enclosing a loose formation of interconnected fibre cells sandwiched in between [35]. The phylogenetic placement of placozoans remains highly contentious (e.g. [28,34,36]). Analyses of the *Trichoplax *genome have revealed a degree of genetic complexity that was totally unexpected given its morpho-anatomical simplicity [36]. As the only non-sponge animal lacking a nervous system, *Trichoplax *is clearly of pivotal importance for understanding the origin of neural cells and synapses.

The gene set investigated in the present study comprises the main scaffold proteins of the PSD, and their more significant interacting partners as recognised in the recent literature (e.g. [15-18]). It is essentially the gene set analysed in [2]. Similarity searches for PSD proteins were conducted against the genomes of *M. brevicollis, T. adhaerens*, several non-metazoan eukaryotes (as outgroups) and a represensative sampling of metazoan species. Our analyses provide evidence for the presence of a substantial subset of functionally important scaffold PSD proteins in the common ancestor of choanoflagellates and metazoans, and for secondary loss of the master scaffold protein Shank in placozoans.

## Methods

### Data collection

**Figure 1:**
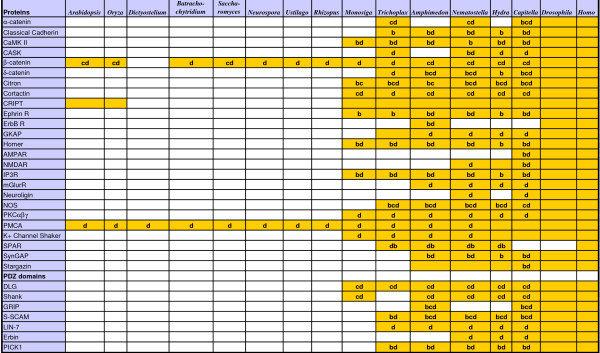
**Summary of the occurrence of post-synaptic proteins in the investigated genomes**. Yellow fields indicate presence of an orthologue as determined from the corresponding gene tree topology. Letters correspond to the three additional confidence criteria for orthology assignments defined in the Methods section: b, ML bootstrap value >= 70% and/or Bayesian posterior probability >= 0.95; c, congruence between at least two partitions of the same protein or between different domains; d, conservation of domain architecture. Note that *Homo *and *Drosophila *have been used as reference taxa for defining the orthology groups.

The protein set examined in this study is listed in Fig. 1 and Additional file 1. It is the same as in ref [2], except that we discarded GABA-B receptor, Tamalin and LIM kinase (because we were unable to find evidence from the literature for physical interaction of these proteins with the PSD scaffold), as well as Kir and Mint (the latter is a presynaptic adaptor protein [37] ; the former is a large family of inwardly rectifying potassium channels which is not particularly associated with the PSD, see [38]). In addition to the full protein alignments, we built a separate alignment for PDZ domains (as in ref [2] but with an extended gene sampling) and constructed specific alignments for the SAM and SH3 domains of Shank. The starting data set [2] comprised sequences from *Saccharomyces cerevisiae *(Ascomycota, Fungi), plants (*Arabidopsis thaliana *and *Oryza sativa*), *Dictyostelium discoideum *(Ameobozoa), *Amphimedon queenslandica *(Demospongiae, Porifera), *Nematostella vectensis *(Anthozoa, Cnidaria), *Drosophila melanogaster *(Hexapoda, Arthropoda) and *Homo sapiens*. These files were updated by TBLASTN searches (using proteins from *Drosophila melanogaster *as input sequences) on the most up-to-date collections of predicted transcripts from plants (*Arabidopsis thaliana *and *Oryza sativa*), *Dictyostelium discoideum *(Ameobozoa), fungi (*Batrachochytridium dendrobatidis*, *Saccharomyces cerevisiae, Neurospora crassa, Ustilago maydis, Rhizopus oryzae*), *Monosiga brevicolis *(Choanoflagellata), *Trichoplax adhaerens *(Placozoa), cnidarians (*Hydra magnipapillata*, Hydrozoa, and *Nematostella vectensis*, Anthozoa), and *Capitella *sp. (Lophotrochozoa, Annelida). An E-value threshold was determined empirically for each protein family by using the E-value associated with the closest paralogue retrieved from Blast of the fly protein against the fly genome (see Additional file 2: E-values used for each family, with names and accession numbers of the closest fly paralogues).

### Alignments and phylogenetic analyses

Sequences retrieved from Blast searches were automatically integrated into the corresponding alignments and aligned using CLUSTALW, implemented in our in-house automated Blast and alignment pipeline. Alignments were slightly corrected manually for elimination of major mistakes. Ambiguous regions were identified by visual inspection and removed manually and positions containing more than 80% of missing data were deleted. When several non-overlapping partial sequences were present in the same alignment, the alignment was split into several partitions to avoid artificial grouping in the trees of non-overlapping sequences (observed in preliminary Maximum Likelihood analyses). These partitions, labelled "A, B, C, ..." (see Additional file 1), were analysed separately. The alignments are given in Additional file 3.

Maximum-Likelihood (ML) analyses were performed using the PhyML program [39], with the WAG model of amino-acid substitution and a BioNJ tree as the input tree. A gamma distribution with four discrete categories was used in these ML analyses. The gamma shape parameter and the proportion of invariant sites were optimised during the searches. Branch support was tested with bootstrapping (100 replicates). Bayesian analyses were done with Mr Bayes under the WAG model (prset=invgamma). For each data set, two searches were run in parallel for 500,000 generations, representing 20 times the number of generations discarded as burn in (first 25,000 trees, determined by stabilisation of the likelihoods). We estimated convergence by checking the "average standard deviation in partition frequency values across independent analyses", using a threshold value of 0.05. Since for most alignments the number of phylogenetically informative positions is low with respect to the number of sequences, trees contain a high number of unstable nodes and therefore it was not possible to constrain searches for convergence with a lower threshold value. In some cases, this threshold of 0.05 was not reached after 500,000 generations (and concomitantly stationarity of the likelihoods was not reached after 25,000 generations). Then, burn-in was expanded and search was prolonged until the index had dropped below 0.05. Posterior probabilities were estimated by constructing a majority-rule consensus of trees sampled every 100 generations. Trees were rooted as in ref [2].

After preliminary (not shown) phylogenetic analyses, all (protein/species) absences were further tested by additional Blast searches on the full (when possible, assembled) nucleotide genome sequences of the corresponding species, with a very relaxed E-value (10) to ensure that these genes have not been missed due to limitations of the initial Blast searches (i.e. the use of predicted transcript data sets, or too strict E-values). In several cases, these genomic searches detected protein orthologues that had been missed during initial searches on predicted transcripts (e.g. IP3R in *Hydra magnipapillata*; PMCA in *Capitella *sp.). In these cases, protein alignments were updated using the procedure described above, prior to final phylogenetic analyses (trees shown in Additional file 4).

### Orthology assignments

An orthology group is here defined as the clade containing all sequences that are more closely related to the fly and/or human sequence(s) of reference than to any paralogue of ancient origin (i.e. resulting from duplication before the last common ancestor of bilaterians). Our orthology assessments therefore rely primarily on gene tree topologies, and the yellow fields in Fig. 1 indicate the presence of an orthologue, based on the corresponding gene tree. Three additional criteria were taken into account to estimate confidence for these orthology assignments: (i) support values (ML bootstrap support >= 70% and/or Bayesian posterior probability >= 0.95) ("b" in Fig. 1), (ii) congruence between separate analyses of partitions of the same protein alignment (labelled A, B, ...: see above) or between different domains of the same protein ("c" in Fig. 1), (iii) comparison of domain architectures reconstructed using Smart http://smart.embl-heidelberg.de/ and Pfam http://pfam.sanger.ac.uk/search. Domain architecture was considered as conserved ("d" in Fig. 1) if the main functional domain(s) existing in the human and fly proteins were present and showed similar arrangement. These domain reconstructions are shown in Additional file 5. They were systematically done for the *Monosiga *and *Trichoplax *putative orthologues of human PSD proteins. In addition, we compared the five C-ter residues of PDZ-binding proteins in search of PDZ ligand conservation (Additional file 6), but the extent of sequence variability observed among PDZ ligands across taxa and proteins is so high that in practice it is generally impossible to conclude either for or against presence of a PDZ ligand. In addition, intron positions and frames were determined for the Shank proteins using NCBI sequence viewer (human and fly proteins) or JGI browse tool (proteins from *Capitella, Nematostella *and *Monosiga*) and the alignment procedure described above.

### Reconstruction of protein acquisitions and losses onto the taxon phylogeny

Presence/absence of the PSD proteins across our species sampling was formalised into a taxon/character matrix. These characters were mapped onto the taxon phylogeny under parsimony and a Dollo model of gene gain/loss (each protein has been acquired only once - no convergence - and proteins can be lost but not re-acquired - no reversion). We used the taxon phylogeny of [34], as it is the most comprehensive phylogenomic study of basal metazoan relationships to date. This means in particular that our interpretation of the data relies upon a hypothesis of eumetazoan monophyly (here represented by Cnidaria + Bilateria). However, basal metazoan relationships remain controversial (see alternative views in [28,29,31]), and the reconstruction may differ under alternative topologies. Since the relationships between sponges, *Trichoplax *and eumetazoans (here represented by cnidarians and bilaterians) were unsupported in ref [34], the reconstruction was done under the three possible tree topologies.

## Results and Discussion

### Reconstructing evolutionary acquisitions and losses of the major PSD components

**Figure 2:**
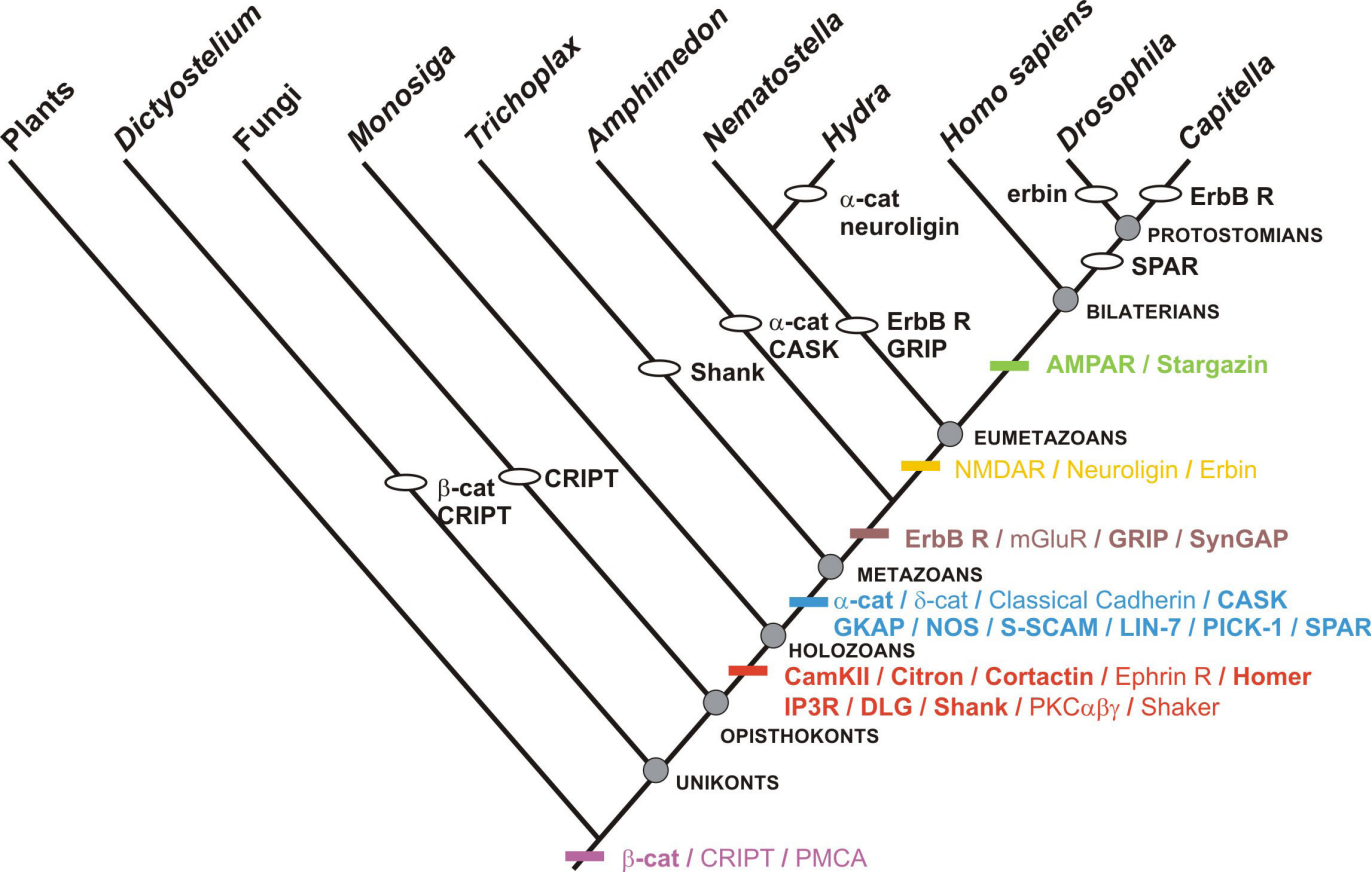
**Gains (coloured dashes) and losses (ellipses) of post-synaptic proteins reconstructed onto the taxon phylogeny under the parsimony criterion**. Placozoans are here placed as the sister-group to other metazoans. The total number of gains + losses is 45 (see less parsimonious reconstructions obtained for alternative placements of placozoans in Additional file 6). Events are labelled in bold when the corresponding gene orthology is supported by at least two confidence criteria in addition to gene tree topology (see explanations in Methods); otherwise they are labelled in normal font.

**Figure 3:**
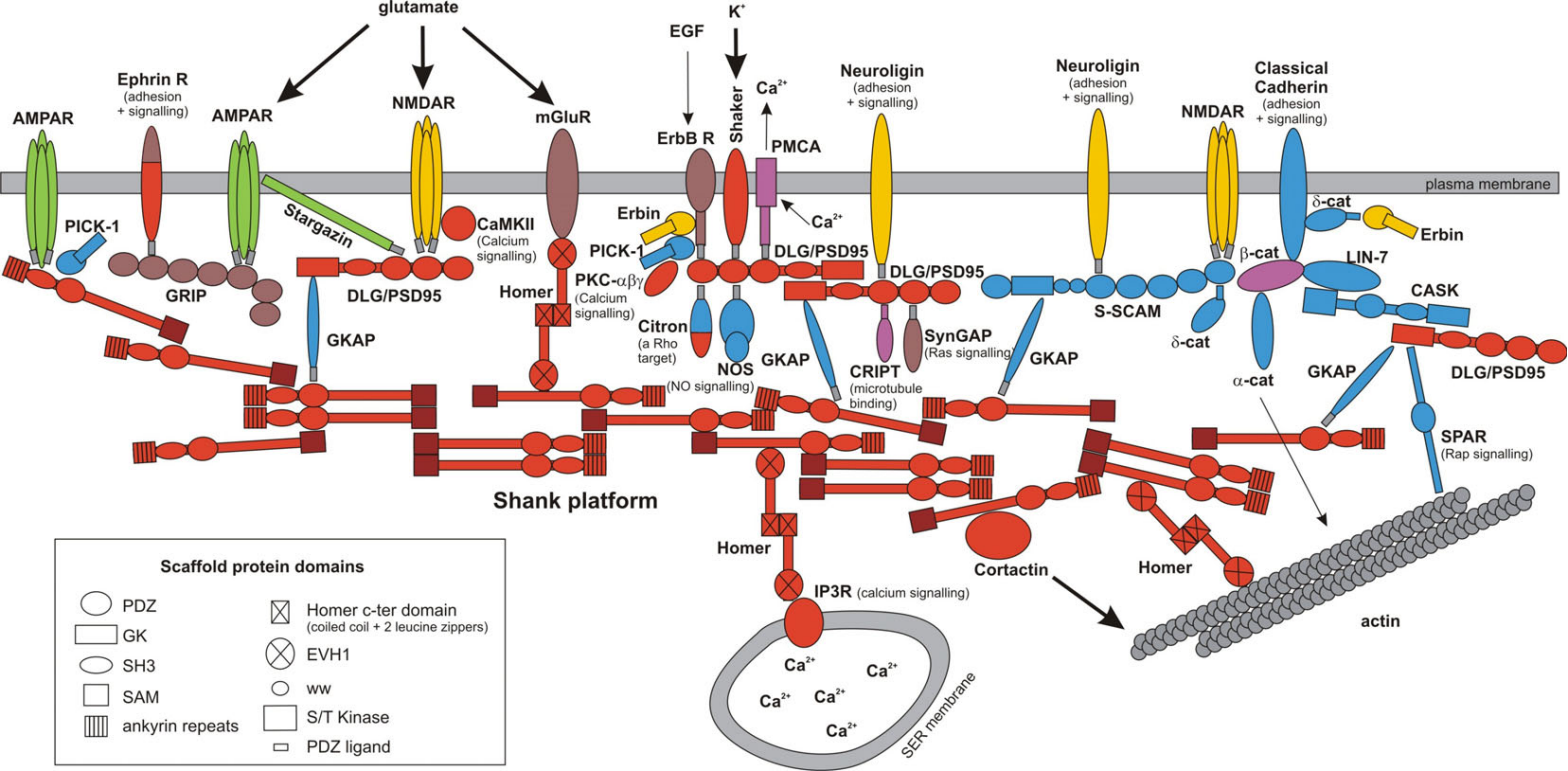
**Graphic representation of the mammalian post-synaptic density, with the origination period of the genes indicated by the same colours as in Fig. 2**. Domain structures are represented only for scaffold proteins. For most PDZ-binding proteins, the PDZ ligand has been labelled in grey, because the extent of sequence variability observed among PDZ ligands across taxa and proteins is so high that it is generally impossible to conclude either for or against the presence of a PDZ ligand (except for CRIPT and δ-cat; see Additional file 4). A darker red colour has been used for the Shank SAM domain, as it might not have been present in the Shank protein of the common choanoflagellate-metazoan ancestor (see text). Note that the general configuration of the post-synaptic scaffold has been completely re-drawn with respect to [2] (their Fig. 1), to account for data available in the recent literature (e.g. [17,18]).

Starting from the PSD protein and domain alignments of ref [2] we have extended the taxonomic coverage by adding genes retrieved from the complete genomes of several additional fungal species, the choanoflagellate *Monosiga brevicolis*, the placozoan *Trichoplax adhaerens*, the cnidarian *Hydra magnipapillata *and the annelid *Capitella *sp. Protein/domain orthology was determined based on gene trees (Additional file 4) and comparison of domain structures (Additional file 5). Results are summarised in Fig. 1, with a more detailed overview in Additional file 1. The most parsimonious reconstruction of protein acquisitions and losses is shown in Fig. 2 (see less parsimonious reconstructions obtained for alternative placements of *Trichoplax*, in Additional file 7). The inferred order of emergence of the genes is indicated by a colour code in Fig. 2 and Fig. 3. In Fig. 2, gene gains/losses are labelled in bold when orthology is supported by at least two criteria of confidence (in addition to gene tree topology; see Fig. 1 and Methods); otherwise they are labelled in normal font.

### PSD components originated in two main steps

Protein acquisitions are concentrated in the two branches preceding and following the divergence of choanoflagellates in the opisthokont phylogeny (Fig. 2). A significant protein set originated in an exclusive ancestor of choanoflagellates and metazoans. This includes the scaffold proteins Shank, Homer and DLG, and the other PSD components CamKII, Citron, Cortactin, Ephrin R, IP3R, PKCαβγ and Shaker K^+ ^channels. Another set, including the scaffold proteins CASK, GKAP and S-SCAM (= Magi), and the other PSD components α and δ-catenin, classical Cadherins, NOS, LIN-7, PICK1 and SPAR, is synapomorphic for the Metazoa. Very few proteins are of more ancient origin: PMCA (present in all investigated eukaryotes), β-catenin (present in plants and fungi) and CRIPT (present in plants).

A significant implication is that several core components of the mammalian PSD (red colour in Fig. 3) originated not only before the acquisition of synapses and nerve cells (absent in sponges and placozoan), but even before the acquisition of multicellularity, given that the choanoflagellate-metazoan ancestor was unequivocally unicellular [1,8]. Notably, the scaffold proteins Shank, Homer and DLG/PSD95, known in mammalians to structure the post-synaptic density by linking a huge variety of target proteins (e.g. membrane receptors, components of signal transduction pathways, cytoskeleton-interacting proteins, etc.), originated before the divergence of the choanoflagellates. In our analyses, the orthology of Homer and DLG between choanoflagellates and metazoans is well supported by the corresponding gene trees, the conservation of their domain structure, high bootstrap support (for Homer) and the congruence between the evolutionary histories of the domains (for DLG) (see Fig. 1).

### Shank emerged before the divergence of choanoflagellates

The Shank protein is considered as the "master-scaffolding" molecule of the PSD [17,18]. Our PDZ tree identifies a putative choanoflagellate orthologue of metazoan Shank PDZs (PDZ tree in Additional file 4). This orthology assignment is not supported by statistical indices, but several additional lines of evidence argue in favour of orthology. The *Monosiga *protein that contains the putative Shank PDZ shows the combination of protein-interacting domains typical of vertebrate Shanks (N-ter ankyrin repeats, a SH3 domain, a PDZ domain, and a SAM C-ter domain), a domain structure that is unique among vertebrate proteins. Reciprocal Blast searches support orthology between the ankyrin repeats present in this *Monosiga *protein and those of metazoan Shanks (not shown - note that phylogenetic analyses of ankyrin repeats are not feasible). In addition, the putative *Monosiga *Shank protein shares with metazoan Shanks the presence of a conserved N-terminal 126-amino-acids region (upstream of the Ankyrin repeats) with no similarity to any other protein sequence (not shown result of Blast search against NCBI referenced proteins) (see alignment of this N-terminal region in Additional file 8). Within this region, there are two introns with position conserved down to the phase and present in metazoan Shanks as well as in the *Monosiga *protein (red boxes in the alignment, Additional file 8). Finally, we analysed SH3 domains and found that the SH3 domain of the *Monosiga *putative Shank falls in a clade that contains all metazoan Shank SH3s (and two non-Shank SH3s, from *Drosophila *and *Hydra*) (see SH3 tree in Additional file 4).

These observations indicate that *Monosiga *has a *bona fide *orthologue of Shank. Although there is no experimental evidence that in the choanoflagellate cell Shank acts like its mammalian orthologues, the conserved domain combination suggests comparable scaffold properties. In addition, some of the proteins known in mammalians to interact directly with Shank (including Homer, the IP3 receptor, and Cortactin) are also present in *Monosiga*.

The history of Shank has been slightly more complex in the details. Our phylogenetic analyses suggest that the SAM C-ter domain of *Monosiga *Shank might not be orthologous to the SAM domains present in some (but not all) metazoan Shanks (see SAM tree in Additional file 4). A SAM domain might thus have been added independently in choanoflagellate and metazoan Shanks. This convergence event could have occurred by chance, or it may have been favoured by similar functional constraints acting on the Shank protein. A second peculiarity of choanoflagellate Shank is that its SH3 domain is located between PDZ and SAM, whereas in metazoans it is placed between the Ank repeats and the PDZ domain, indicating that a domain inversion took place in one of these two lineages. We compared intron positions along the Shank proteins of metazoans and *Monosiga *without finding evidence for or against this hypothesis of domain inversion (Additional file 8).

### Evolution of the PSD protein repertoire within the Metazoa

Compared to the gene complement present in eumetazoan genomes, different gene subsets happen to be absent in the two nerve-less species *Amphimedon queenslandica *(sponge) and *Trichoplax adhaerens *(placozoan). This makes the reconstruction of acquisition/losses in this region of the tree highly sensitive to the phylogenetic placement of placozoans with respect to sponges and eumetazoans. *Trichoplax *lacks orthologues of Shank (present in *Monosiga*, sponges and eumetazoans), GRIP, ErbB R, mGluR and SynGAP (present in sponges and at least some eumetazoans). If *Trichoplax *is the sister-group to other metazoans (Fig. 2), then GRIP, ErbB R, mGluR, and SynGAP were absent in the metazoan ancestor and are synapomorphic for sponges + eumetazoans (brown colour in Fig. 2 and 3). The same taxon phylogeny implies secondary loss of α-catenin and CASK (present in *Trichoplax *and in eumetazoans) in the sponge lineage. Alternative taxon phylogenies (e.g. *Trichoplax *sister-group to eumetazoans as in [34]) imply secondary losses of GRIP, ErbB R, mGluR and SynGAP in placozoans (Additional file 7). Whatever the position of placozoans, the parsimony optimisation suggests secondary loss of Shank in *Trichoplax adhaerens*.

The apparent loss of Shank in placozoans is particularly puzzling as Shank is the main protein responsible for the higher-order structure of the PSD in mammals [18]. However, alternative scenarios cannot be ruled out at this stage, e.g., Shank could have been overlooked due to incomplete coverage of the *T. adhaerens *genome (currently 8×), or the gene might have diverged in the placozoan lineage to the extent that it is no longer recognisable, or it might have been lost in *Trichoplax adhaerens *but not in other placozoan species. If confirmed by future studies, loss of Shank in placozoans would imply disruption or at least considerable alteration of the macromolecular architecture of the PSD-like protein complex. In addition, the *Trichoplax *situation is somewhat difficult to understand when it is realised that Homer and Cortactin, known as direct Shank partners in mammals and present in the *Monosiga *genome, are still present in *Trichoplax *despite apparent absence of Shank. As an alternative possibility, Shank could have been replaced by another protein. Functional work is certainly needed in *Trichoplax *to determine the molecular interactions and cellular functions of these proteins. Whether these genes are expressed in the fibre cells, proposed by some [40] to represent a placozan neural-like cell type, will be important to assess. However, placozoans are not likely to teach us much about the ancestral properties and functions of PSD proteins prior to the emergence of a nervous system, because the absence of Shank (if confirmed) makes their PSD-like complex totally idiosyncratic.

Whereas the emergence of nerve cells and synapses in a eumetazoan ancestor was seemingly not accompanied by a significant expansion of the PSD complex in terms of protein number, the few proteins acquired in this branch are significant with respect to neural function. They include the ionotropic glutamate receptors NMDAR (involved in synaptic transmission) and the Neuroligins (involved in synaptic adhesion and signalling in synaptogenesis, through interaction with their ligands the Neurexins [41,42]). Within eumetazoans, we inferred a few protein losses: ErbB receptor and GRIP (a scaffold protein) in cnidarians, α-catenin and Neuroligin in *Hydra*, SPAR in protostomians, Erbin in *Drosophila *and ErbB receptor in the annelid *Capitella*. Curiously enough, there is no concomitant loss of proteins known to interact with each other (e.g. losses of Erbin are not associated with losses of the ErbB receptor), suggesting that proteome and interactome evolved to a large extent independently, or that protein interactions in non-model organisms differ substantially from what happens in the mammalian synapse.

### What were the ancestral functions of the PSD protein complex?

Comparative genomics contributes to our understanding of hierarchy in biological systems, by sorting ancient components from more recent additions, a basis for making predictions about ancestral interactions and functions. In bilaterian animals, the PSD is tightly linked functionally to intercellular signalling at the synapse. However, the proto-PSD of the choanoflagellate/metazoan ancestor (red + violet colours in Fig. 3) was probably not involved in intercellular signalling, since all post-synaptic membrane receptors linked to the mammalian PSD (the different kinds of glutamate receptors, Neuroligin, ErbB receptor, receptor domain of the Ephrin receptor) are of more recent origin. In addition, the global structure of the ancestral proto-PSD, which lacks several important scaffold proteins (GRIP, S-SCAM, CASK), was certainly much simpler than that of the mammalian PSD.

Shank and its conserved direct or indirect partners appear to form the ancestral core of the post-synaptic protein complex (Fig. 3). The associated functions are actin cytoskeleton regulation (Homer links actin to Shank; the actin-regulating protein Cortactin binds to Shank) and Ca^2+ ^signalling (Homer links the IP3 receptor to Shank; CaMKII and PKCαβγ, also involved in Ca^2+ ^signalling, are present in the choanoflagellate genome). It is thereby proposed here that the main function of the ancestral Shank interactome was to provide a link between intracellular Ca^2+ ^signalling and actin cytoskeleton regulation.

The DLG scaffold protein might have ancestrally formed the centre of an additional interactome, possibly independent from the Shank module (GKAP, the physical link between DLG and Shank in mammals, is absent in *Monosiga*). We identified three DLG partners with clear orthologues in the choanoflagellate genome: CRIPT (a microtubule-interacting protein [43]), the K+ channel Shaker, and the Ca^2+ ^plasma membrane channel PMCA. The choanoflagellate orthologue of Citron (a Rho target) is not likely to interact with DLG as in mammalians because only the most N-ter part of the protein shows homology with mammalian Citron (Fig. 3). These observations suggest ancestral functions of the DLG interactome in concentrating cation channels on particular plasma membrane areas, and in providing a link between these channels and the regulation of microtubule polymerisation.

From a morphological point of view, the choanoflagellate + metazoan clade is characterised by a unique synapomorphy: acquisition of an apical cell complex with a central flagellum surrounded by a collar of microvilli. This is suggested by the close resemblance between apical cell complexes of choanoflagellates and of sponge choanocytes [44]. According to some [45], this flagellum-collar structure would be homologous with the apical complex of eumetazoan sensory cells. Because choanoflagellate collar microvilli are contractile and labile structures (suggesting highly dynamic regulation), and because their cytoskeleton is principally made of F-actin, it is tempting to speculate about a possible functional link between the emergence of the proto-PSD and the acquisition of the flagellum-collar apical complex. This hypothesis can be tested by determining the subcellular localisation and function of the key proteins Shank, Homer and DLG in *Monosiga*. Another stimulating path for future investigation on the ancestral functions of core PSD components will involve the characterisation of physical interactions between the various choanoflagellate PSD-like proteins.

## Conclusion

The backbone of the post-synaptic density, notably featuring the scaffold proteins Shank, Homer and DLG, was acquired in a common ancestor of choanoflagellates and metazoans, in a unicellular context. It was probably not involved in cell-cell communication, but rather in linking transmembrane cation currents, calcium signalling, and cytoskeleton regulation. Future investigations on complete genome sequences of unicellular opisthokonts that are more closely related to the choanoflagellate + metazoan clade than to Fungi (ichthyosporids and *Capsaspora*) [1] might happen to push even earlier the period of origination of these proteins.

After the addition of several novel components (among which GKAP, the link between DLG and Shank, and the CASK/S-SCAM/classical cadherin interactome), metazoan ancestors became endowed with a nearly complete set of PSD proteins (violet + red + blue colours in Fig. 3). Proteins that are specifically involved in synaptic transmission (i.e. AMPAR, NMDAR and mGluR) or adhesion (neuroligin) in eumetazoans were nevertheless absent from this ancestral animal repertoire (except possibly for mGluRs, present in the metazoan ancestor if *Trichoplax *is not the basal-most lineage, see Additional file 7). This suggests that PSD proteins ancestrally acted in a non-synaptic context, in consistence with the probable monophyly of Eumetazoa [34] and its corollary, that the last common ancestor of all metazoan animals was nerve-less. In this ancestor, the PSD-like complex was probably a sub-membrane platform for bringing together molecules involved in cation currents, Ca^2+ ^and other signalling pathways, and cytoskeleton regulation, possibly (but not necessarily) in relation to cell sensitivity to external stimuli. The complex later became integrated into the evolving synapse, in a eumetazan ancestor, through the addition of novel proteins and functionalities.

## Authors' contributions

AA and MM conceived and designed the analyses. AA performed the data collection and the analyses. MM drew the figures. AA and MM wrote the paper. Both authors read and approved the final manuscript.

## Supplementary Material

Additional file 1 **Detailed overview of the results from the phylogenetic analyses**. Grey lines: presence or absence of orthologue(s) for each protein group in each species. White lines: number of orthologues (if any) identified for each alignment or alignment partition. Click here for file

Additional file 2 **Table indicating the threshold E-values used for Blast searches for each family of investigated genes, and the closest paralogue retrieved from the fly genome**. Click here for file

Additional file 3 **Protein sequence alignments used for phylogenetic analyses**. The alignments are in Phylip format. Click here for file

Additional file 4 **Maximum Likelihood phylogenetic trees of the post-synaptic proteins and of the PDZ, SAM and SH3 domains (arranged in alphabetic order)**. Maximum likelihood boostraps (ML bs) and Bayesian posterior probabilities (Bay pp) are indicated only for the main orthology groups. Abbreviations for taxon names: Aqu = *Amphimedon queenslandica*, Ath = *Arabidopsis thaliana*, Bde = *Batrachochytridium dendrobatidis*, Cca = *Capitella*sp., Ddi = *Dictyostelium discoideum*, Dme = *Drosophila melanogaster*, Hma = *Hydra magnipapillata*, Hsa = *Homo sapiens*, Mbr = *Monosiga brevicolis*, Ncr = *Neurospora crassa*, Nve = *Nematostella vectensis*, Osa = *Oryza sativa*, Ror = *Rhizopus oryzae*, Sce = *Saccharomyces cerevisiae*, Tad = *Trichoplax adhaerens*, Uma = *Ustilago maydis*. Except for plant, yeast, slime mold, sponge, fly and human sequences, genes are named by using a neutral code consisting in the taxon abbreviation followed by a letter (different for each protein family) and a number. Thanks to this label, a given gene can be identified across different partitions of the same alignment or different domains of the same protein (e.g. for Shank in the PDZ, SAM and SH3 trees). Click here for file

Additional file 5 **Domain architectures of selected members of the post-synaptic gene families (in alphabetic order)**. Abbreviations used in displays for taxon names are as in Additional file 2. Click here for file

Additional file 6 **The C-ter five residues of the PDZ-binding post-synaptic proteins, for metazoan species and Monosiga**. Click here for file

Additional file 7 **Gains (coloured dashes) and losses (ellipses) of post-synaptic proteins reconstructed with placozoans as the sister-group to sponges, and with placozoans as the sister-group to eumetazoans**. The number of gains + losses is indicated for each tree. Click here for file

Additional file 8 **Comparison of intron positions in the Shank proteins of metazoans and Monosiga**. Overview of intron distribution along the whole protein sequences (A) and detailed view of intron position on the alignment of the N-terminal region upstream of the Ankyrin repeats (B). Click here for file
