# Supplementary material for: The backbone of the post-synaptic density originated in a unicellular ancestor of choanoflagellates and metazoans
Source: BMC Evol Biol. 2010 Feb 3;10:34. doi: 10.1186/1471-2148-10-34 (PMC2824662; doi:10.1186/1471-2148-10-34)
Supplement: Additional file 4 — Maximum Likelihood phylogenetic trees of the post-synaptic proteins and of the PDZ, SAM and SH3 domains (arranged in alphabetic order). Maximum likelihood boostraps (ML bs) and Bayesian posterior probabilities (Bay pp) are indicated only for the main orthology groups. Abbreviations for taxon names: Aqu = Amphimedon queenslandica, Ath = Arabidopsis thaliana, Bde = Batrachochytridium dendrobatidis, Cca = Capitellasp., Ddi = Dictyostelium discoideum, Dme = Drosophila melanogaster, Hma = Hydra magnipapillata, Hsa = Homo sapiens, Mbr = Monosiga brevicolis, Ncr = Neurospora crassa, Nve = Nematostella vectensis, Osa = Oryza sativa, Ror = Rhizopus oryzae, Sce = Saccharomyces cerevisiae, Tad = Trichoplax adhaerens, Uma = Ustilago maydis. Except for plant, yeast, slime mold, sponge, fly and human sequences, genes are named by using a neutral code consisting in the taxon abbreviation followed by a letter (different for each protein family) and a number. Thanks to this label, a given gene can be identified across different partitions of the same alignment or different domains of the same protein (e.g. for Shank in the PDZ, SAM and SH3 trees). [file 1471-2148-10-34-S4.PDF]

α–Catenin partition A

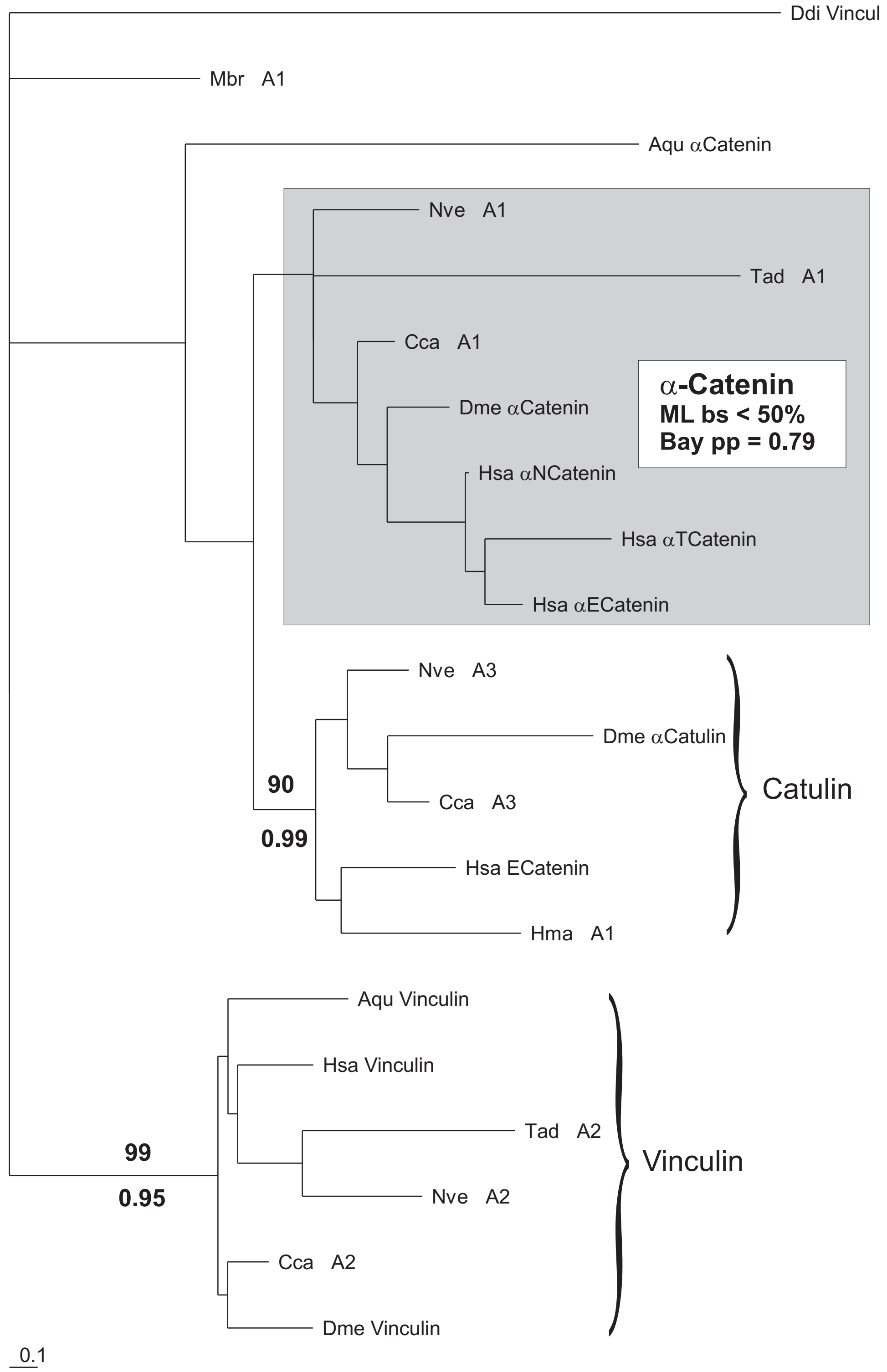

α-Catenin partition B

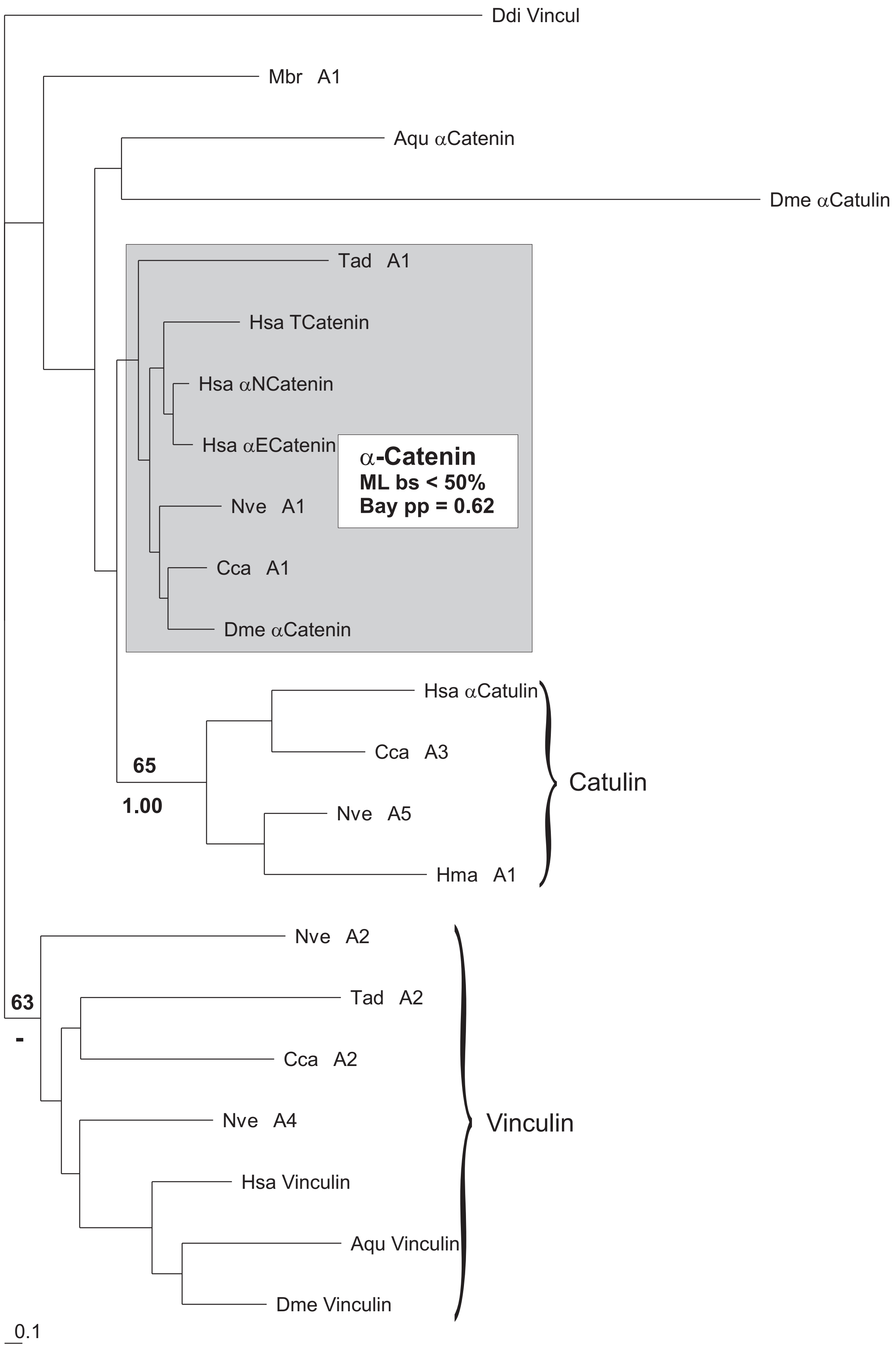

α-Catenin partition C

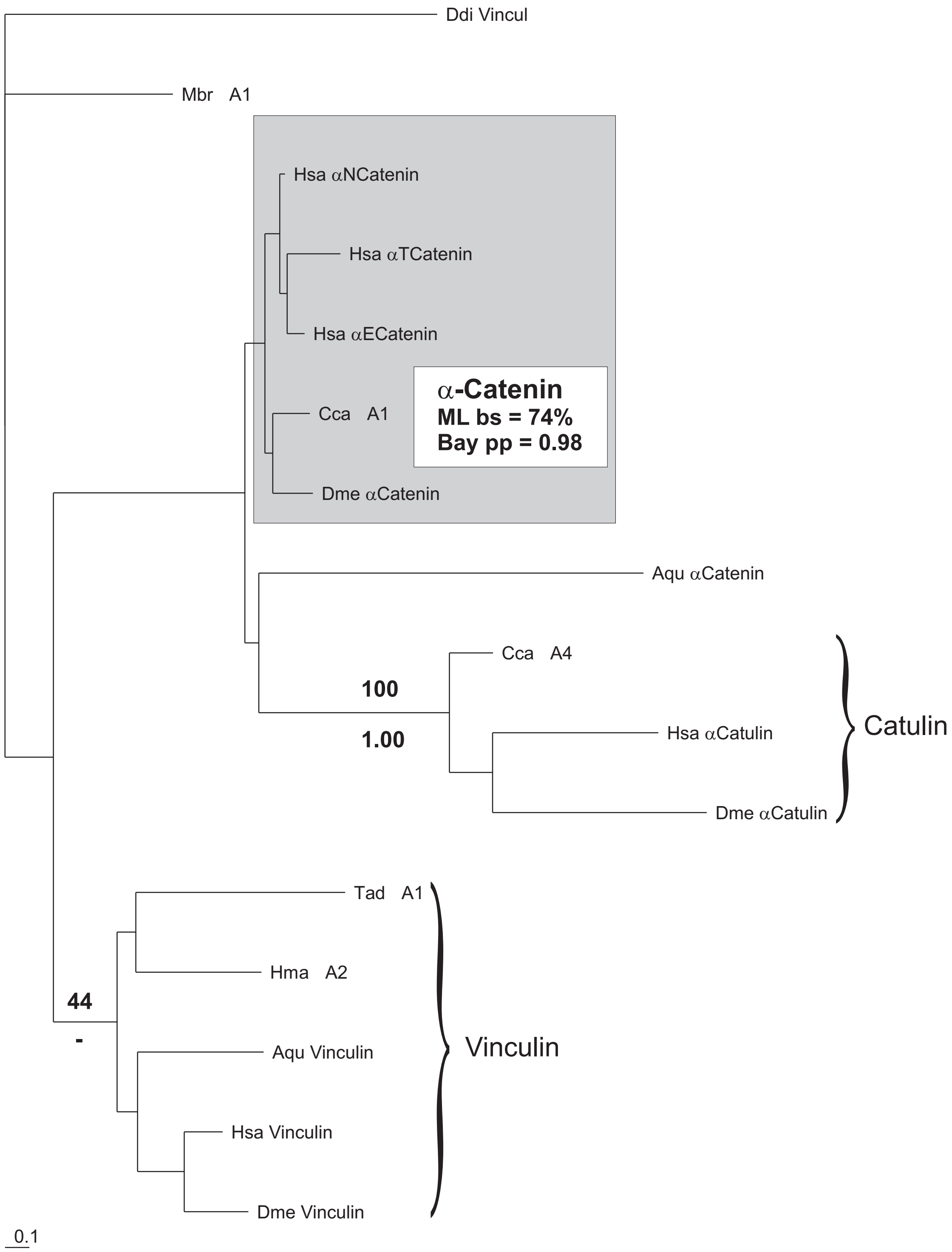

Classical Cadherins

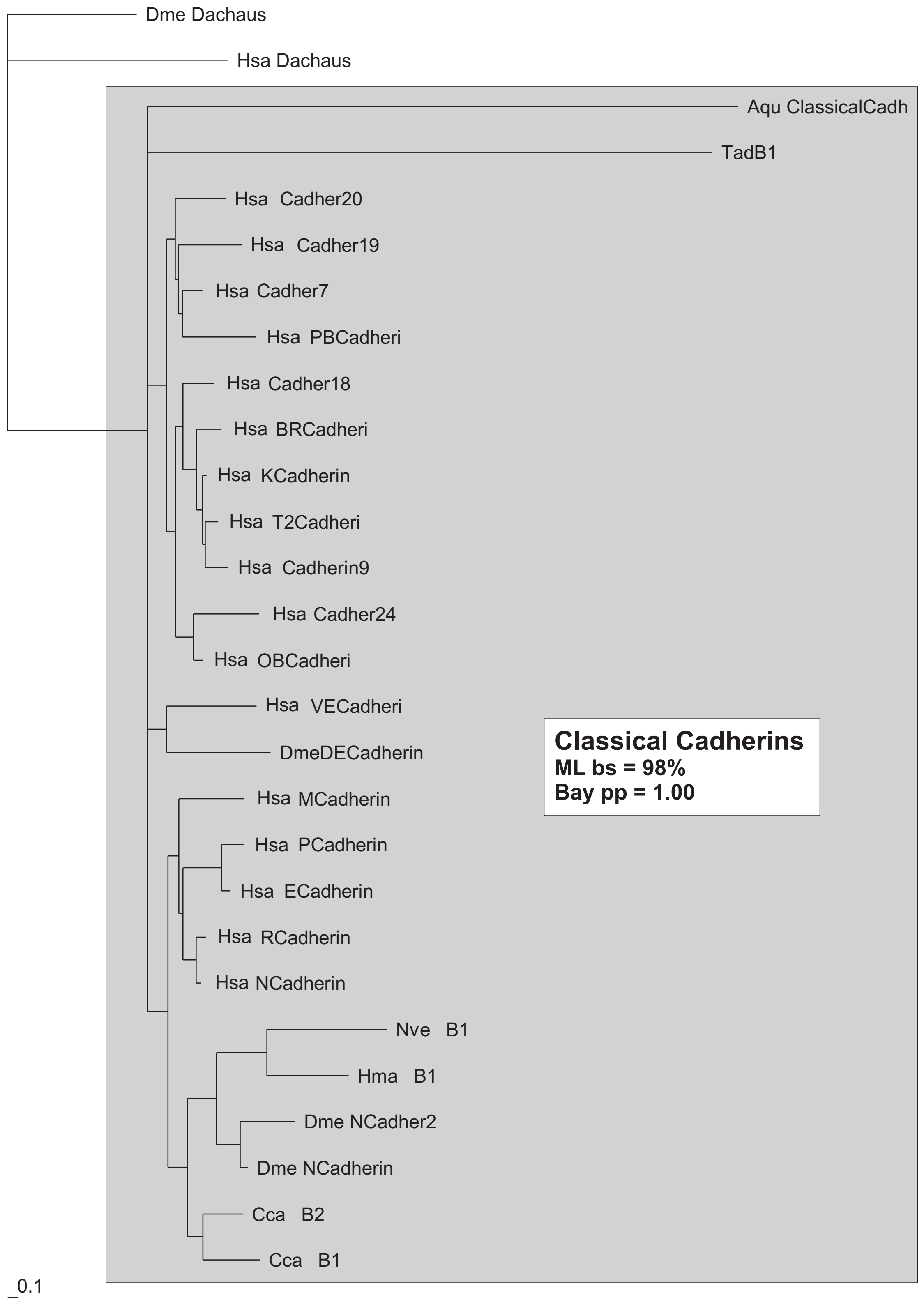

CamKII

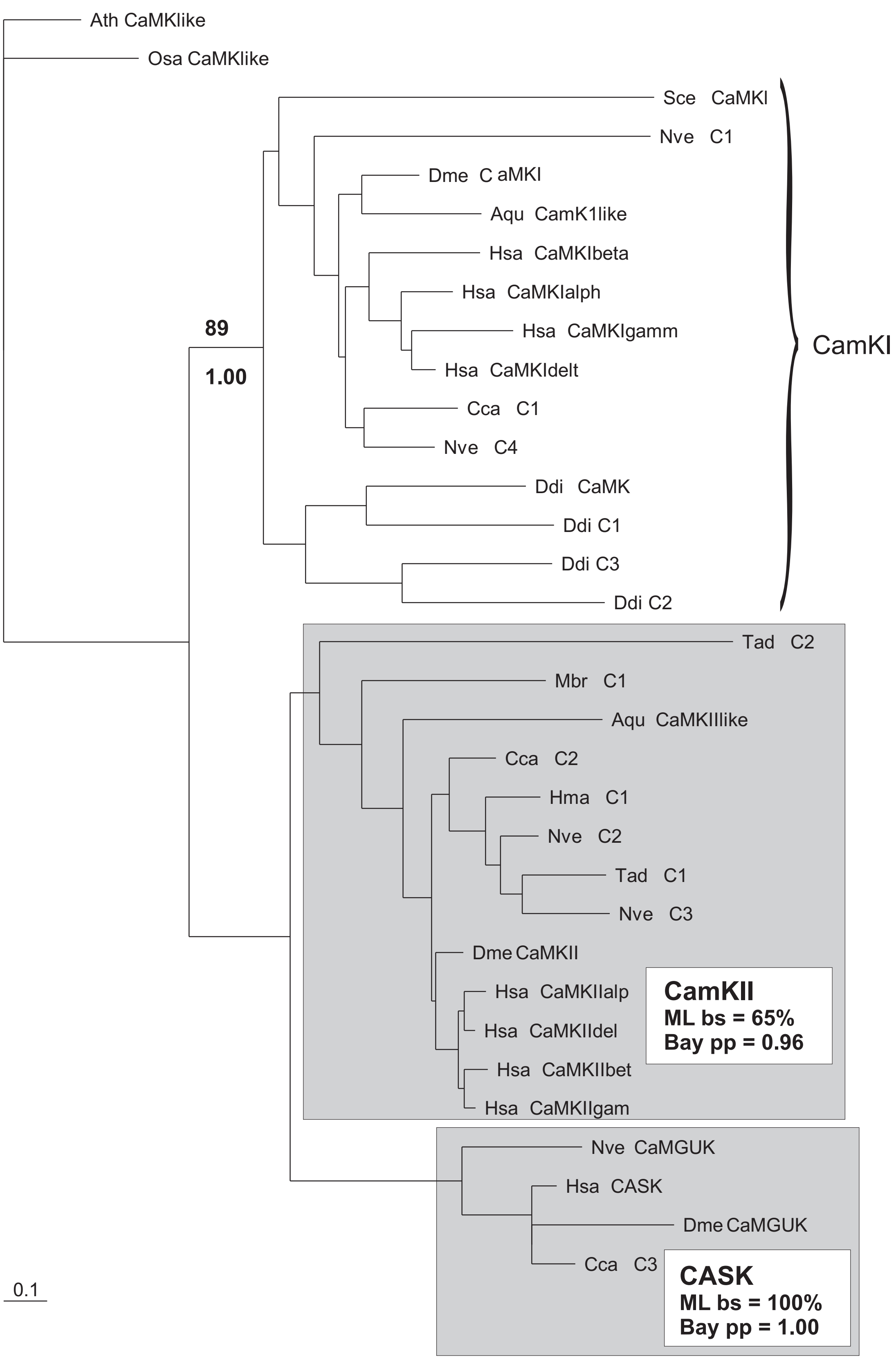

Ath CaMKlike
Osa CaMKlike
Sce CaMKI
Nve C1
Dme C aMKI
Aqu CamK1like
Hsa CaMKIbeta
Hsa CaMKIalph
Hsa CaMKIgamm
Hsa CaMKIdelt
Cca C1
Nve C4
Ddi CaMK
Ddi C1
Ddi C3
Ddi C2
CamKI
89
1.00
Tad C2
Mbr C1
Aqu CaMKIIlike
Cca C2
Hma C1
Nve C2
Tad C1
Nve C3
Dme CaMKII
Hsa CaMKIIalp
Hsa CaMKIIdel
Hsa CaMKIIbet
Hsa CaMKIIgam
CamKII
ML bs = 65%
Bay pp = 0.96
Nve CaMGUK
Hsa CASK
Dme CaMGUK
Cca C3
CASK
ML bs = 100%
Bay pp = 1.00
0.1

Catenins partition A

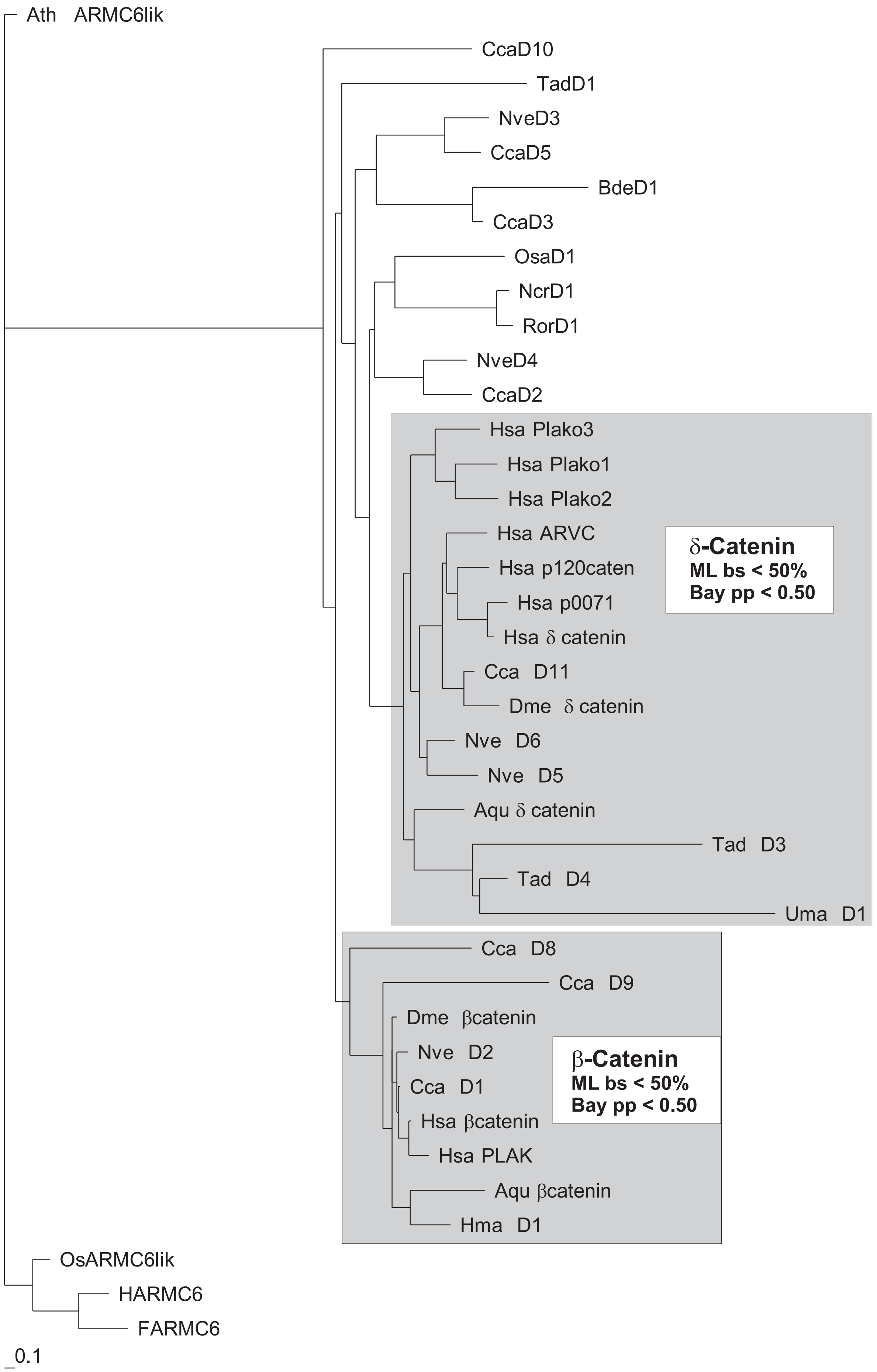

Catenins partition B

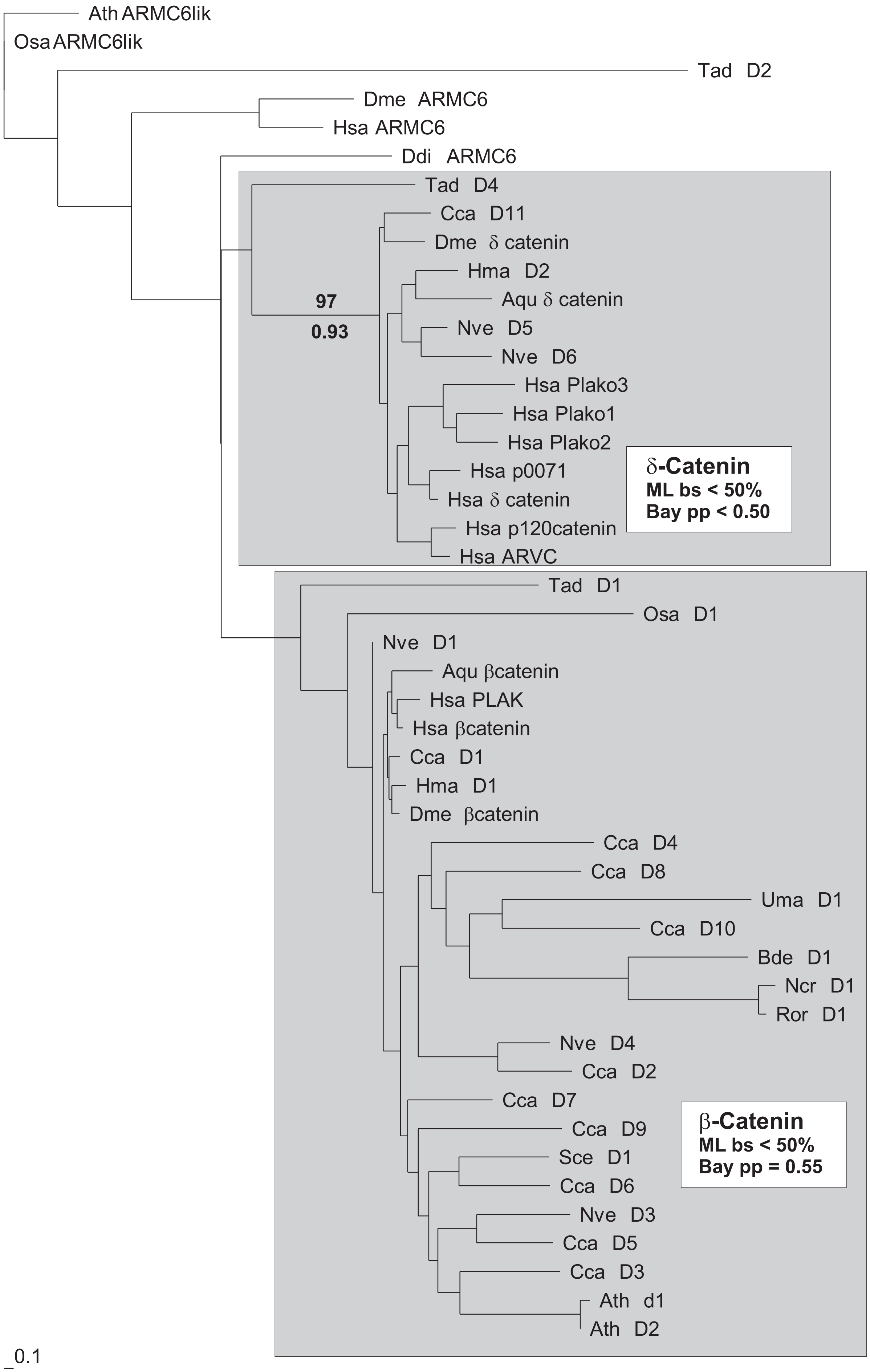

Catenins partition C

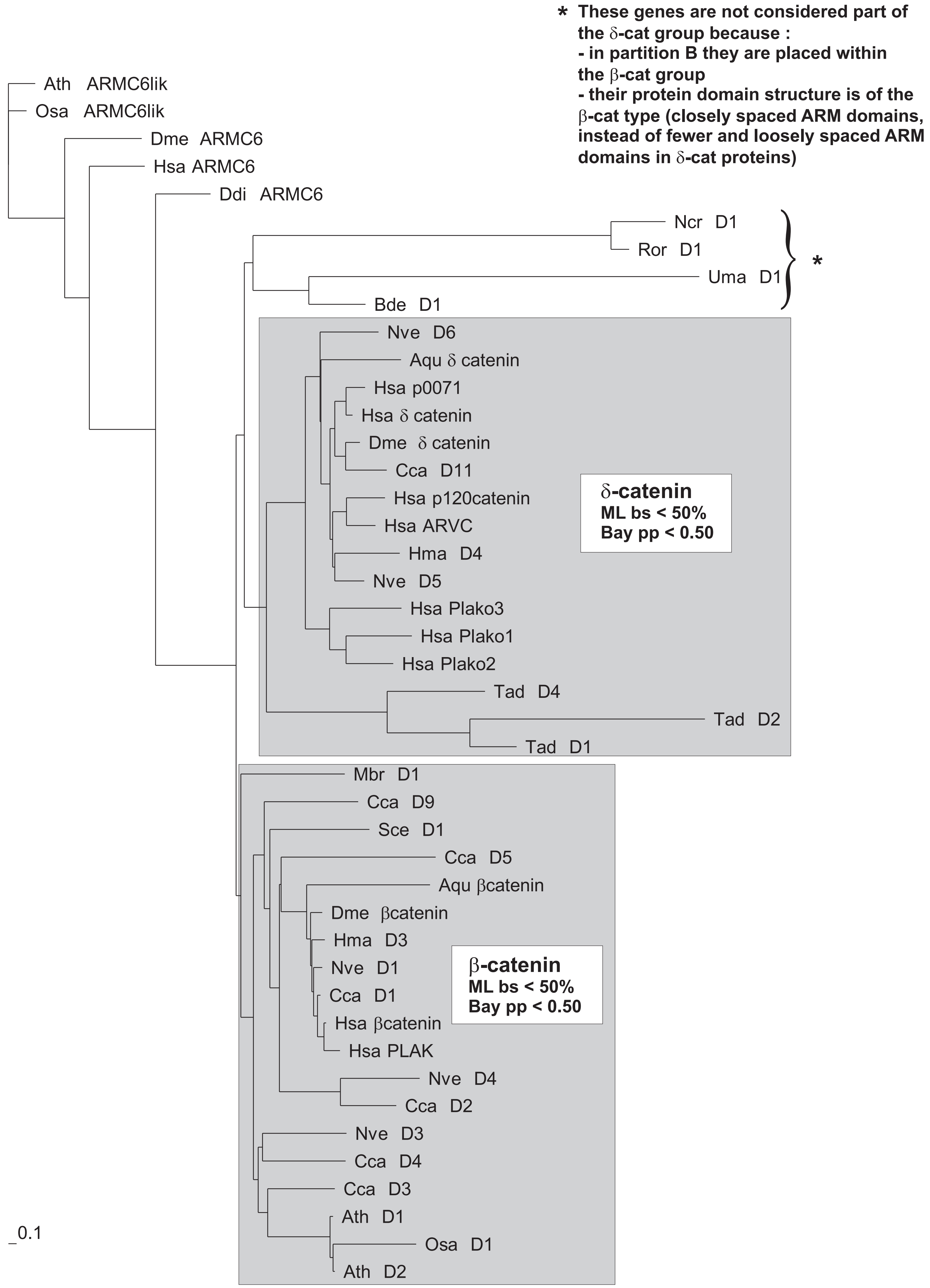

Citron partition A

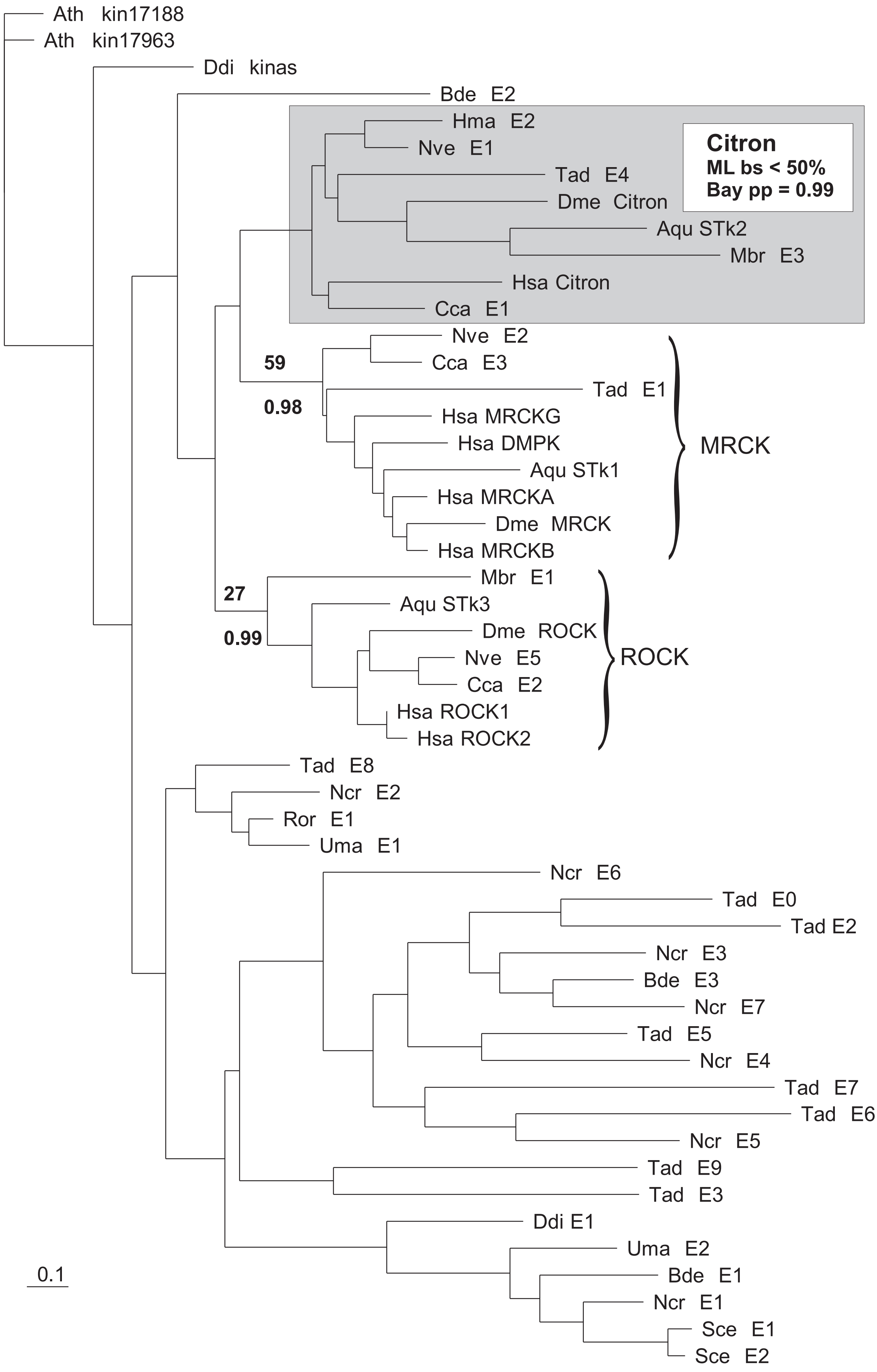

Citron partition B

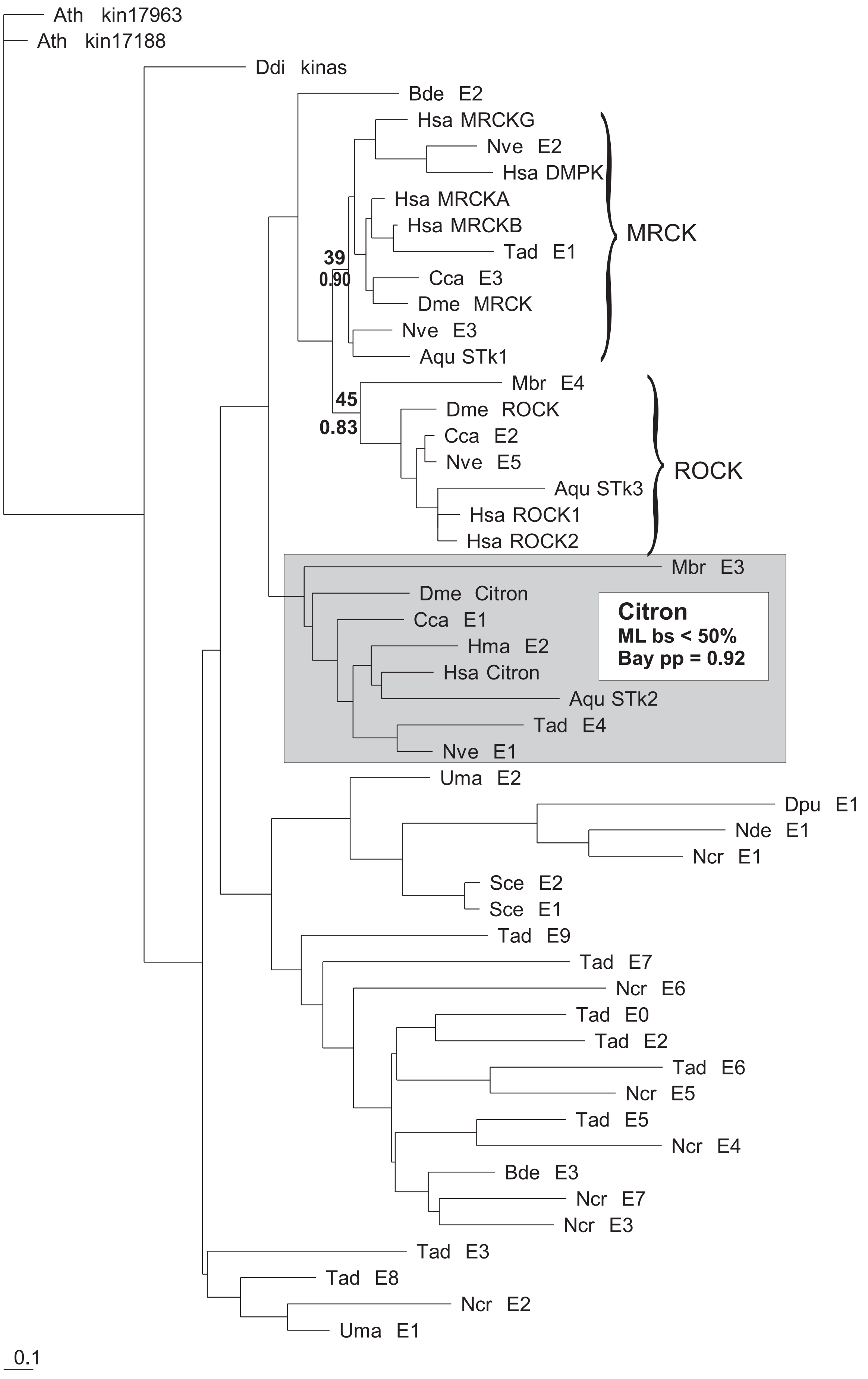

Cortactin partition A

This part of cortactin has no similarity to other proteins

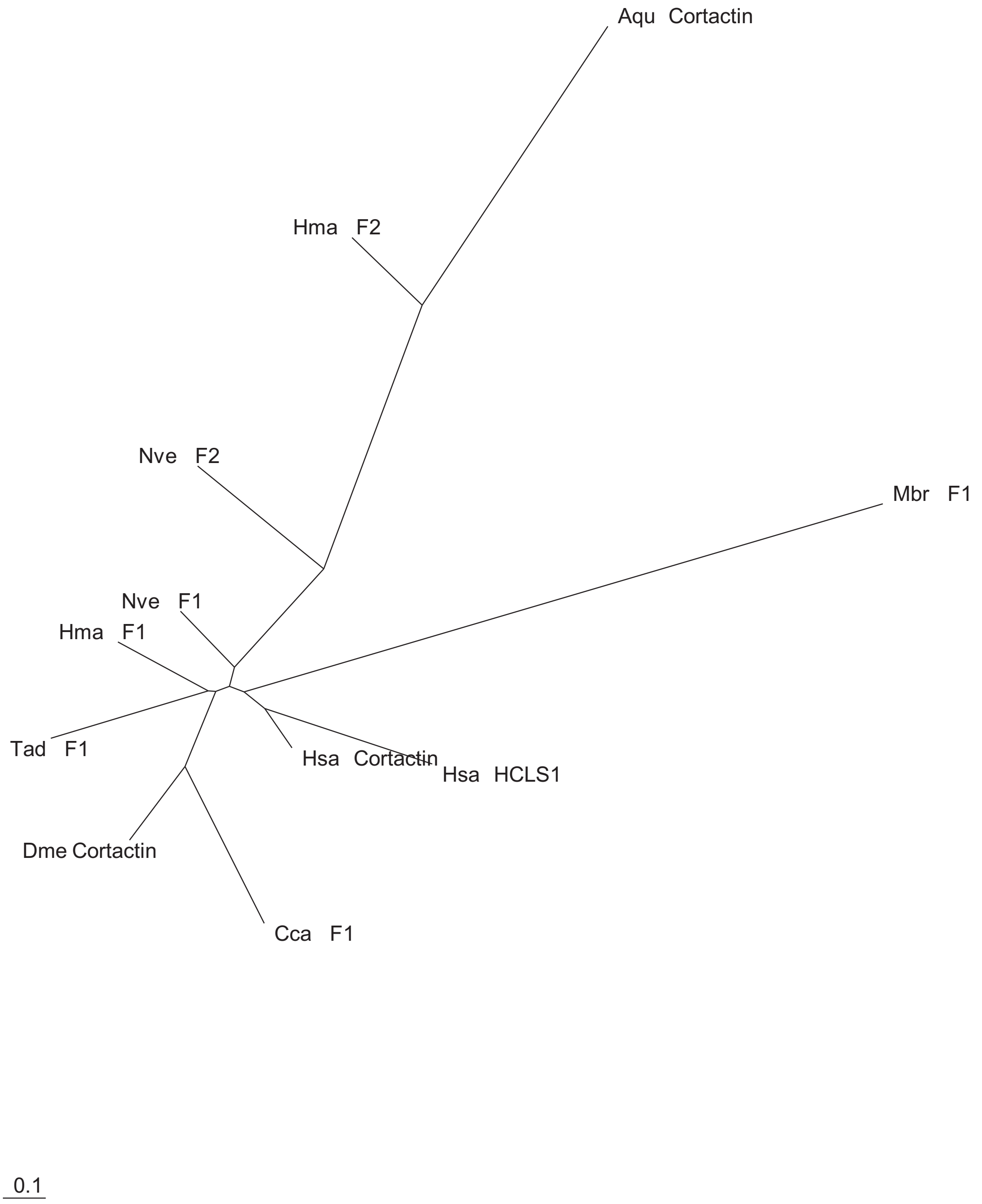

Cortactin partition B

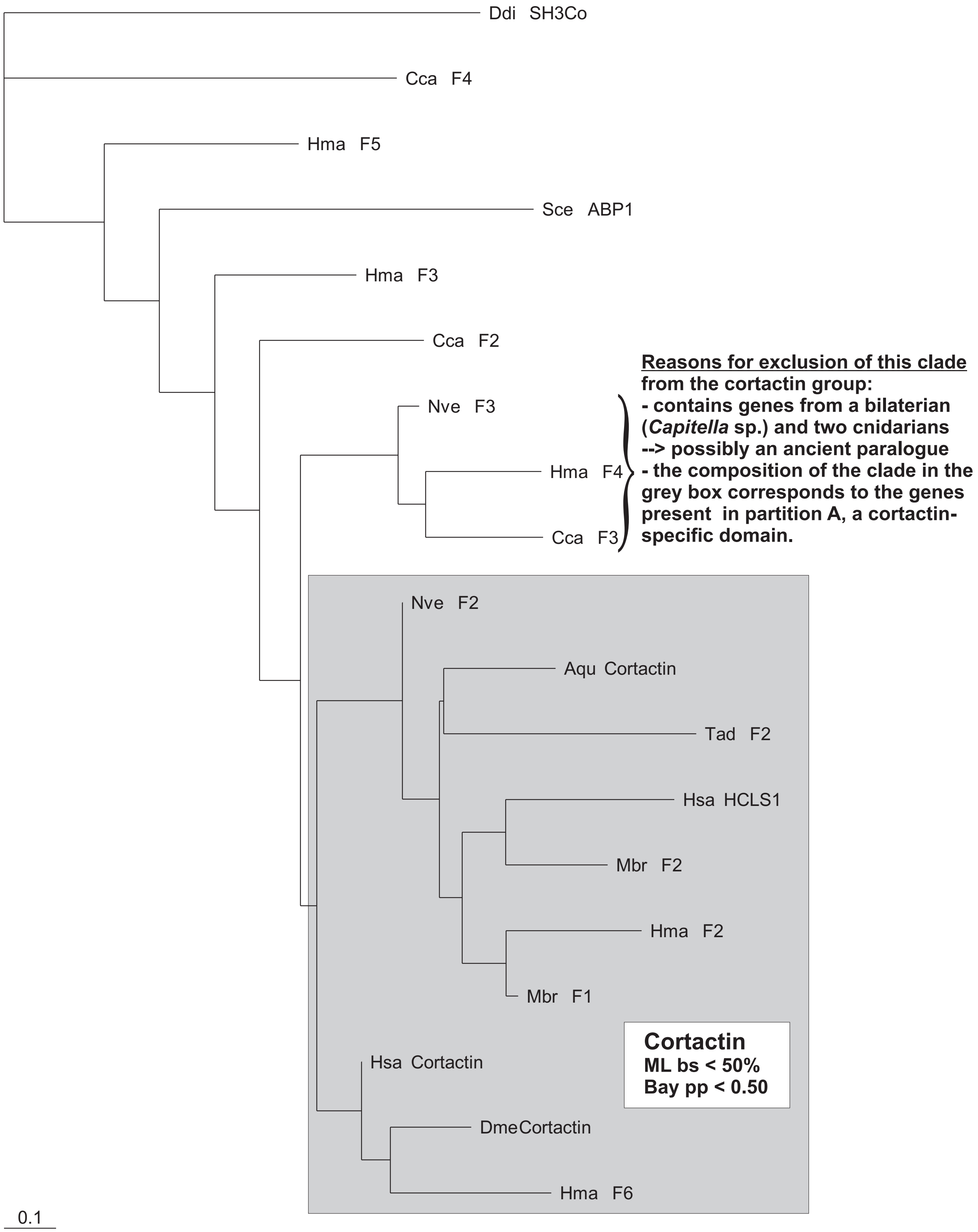

CRIPT

CRIPT has no similarity to other proteins

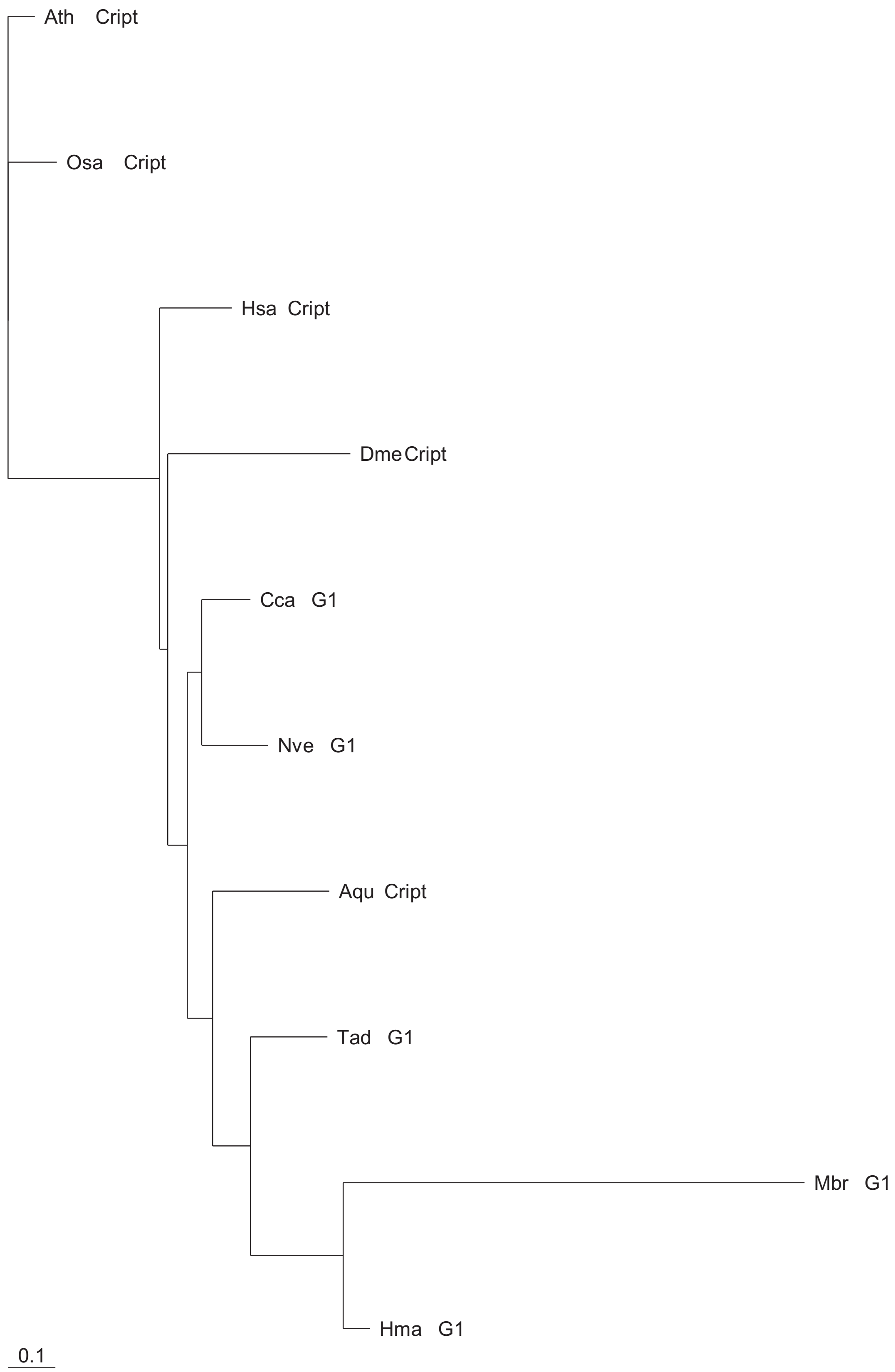

Ephrin R / ErbB R

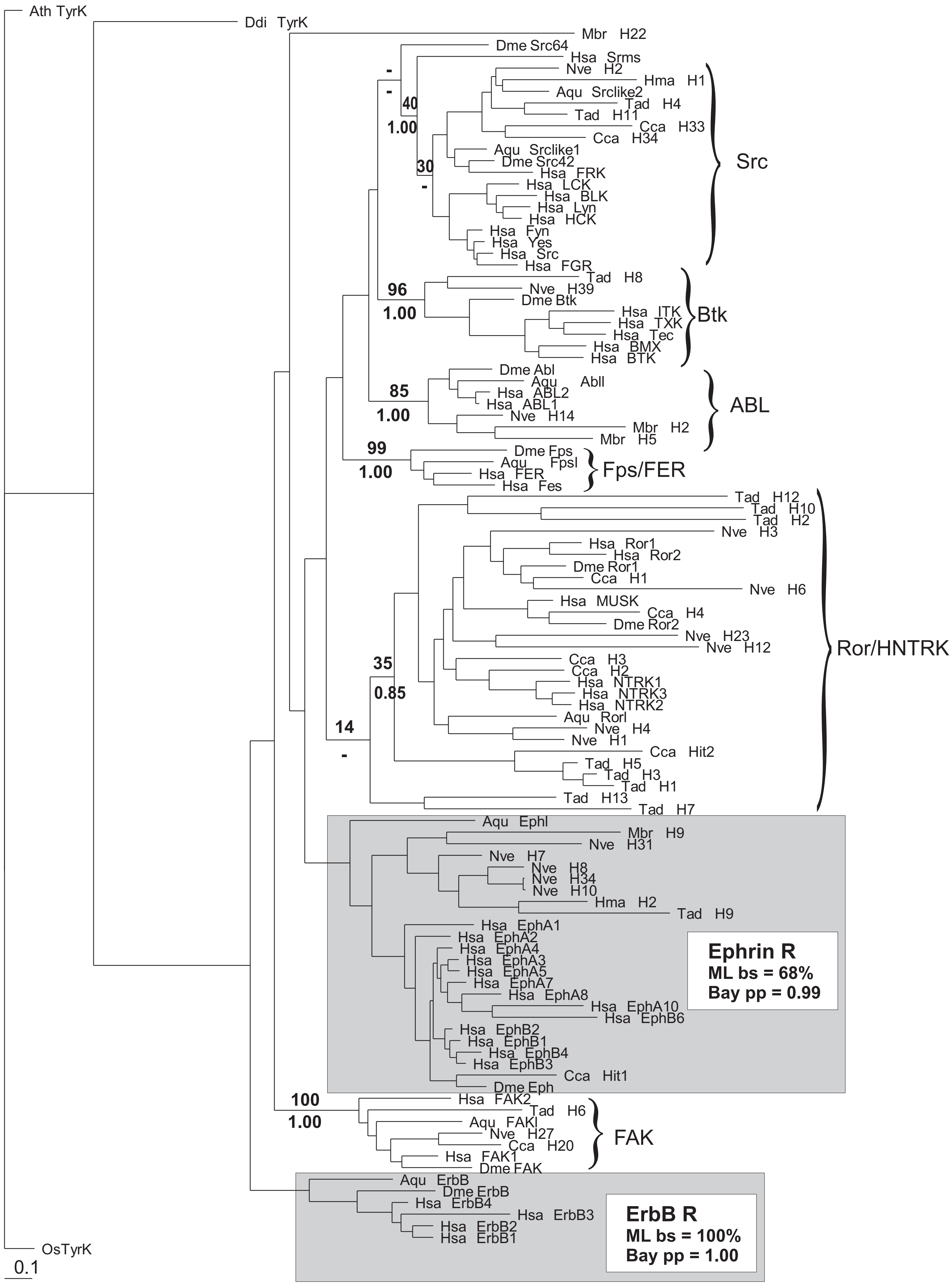

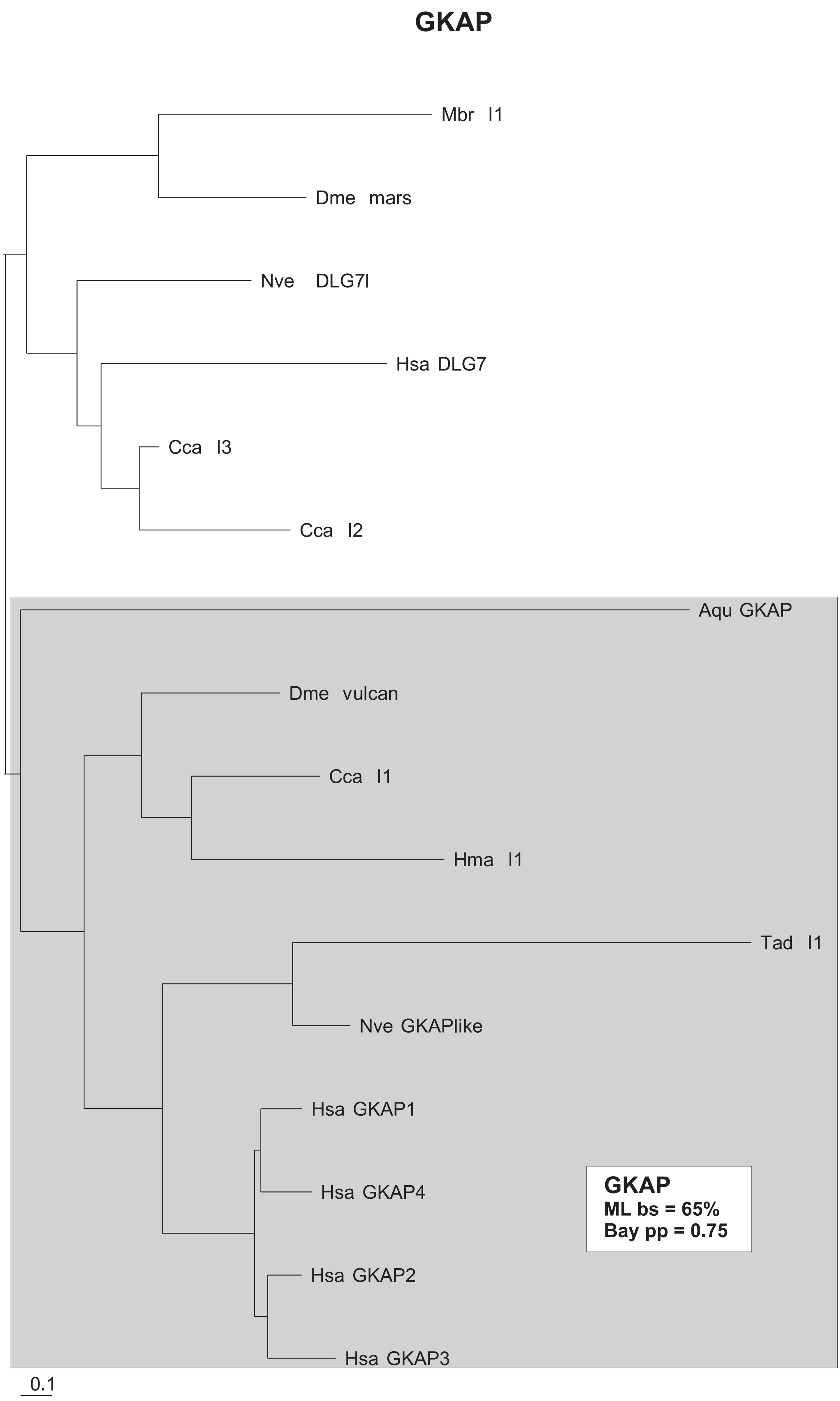
GKAP
Mbr I1
Dme mars
Nve DLG7l
Hsa DLG7
Cca I3
Cca I2
Aqu GKAP
Dme vulcan
Cca I1
Hma I1
Tad I1
Nve GKAPlike
Hsa GKAP1
Hsa GKAP4
Hsa GKAP2
Hsa GKAP3
GKAP
ML bs = 65%
Bay pp = 0.75
0.1

Homer

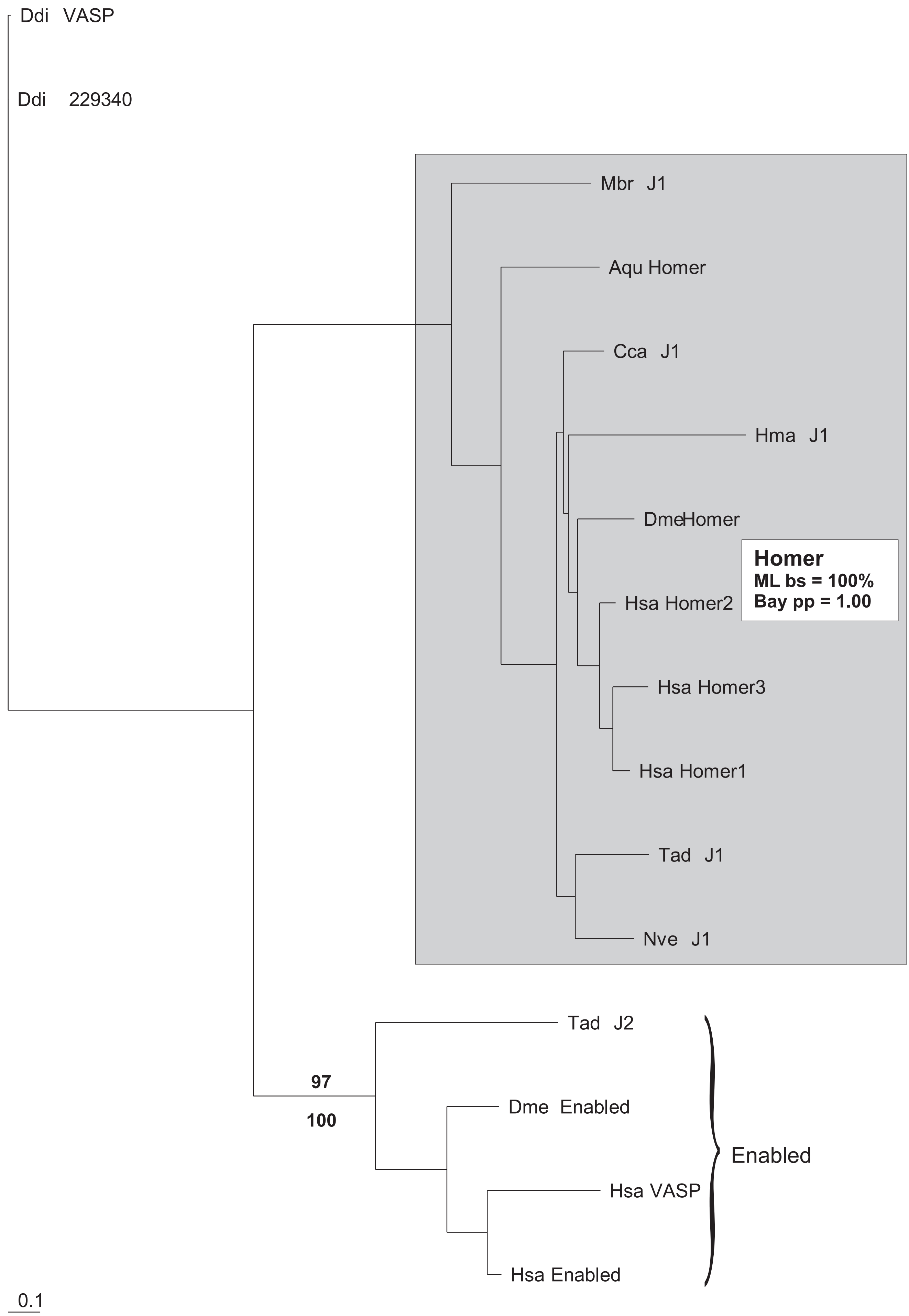
Ddi VASP
Ddi 229340
Mbr J1
Aqu Homer
Cca J1
Hma J1
DmeHomer
Hsa Homer2
Hsa Homer3
Hsa Homer1
Tad J1
Nve J1
Homer
ML bs = 100%
Bay pp = 1.00
Tad J2
97
100
Dme Enabled
Hsa VASP
Hsa Enabled
Enabled
0.1

Ionotropic Glutamate Receptors

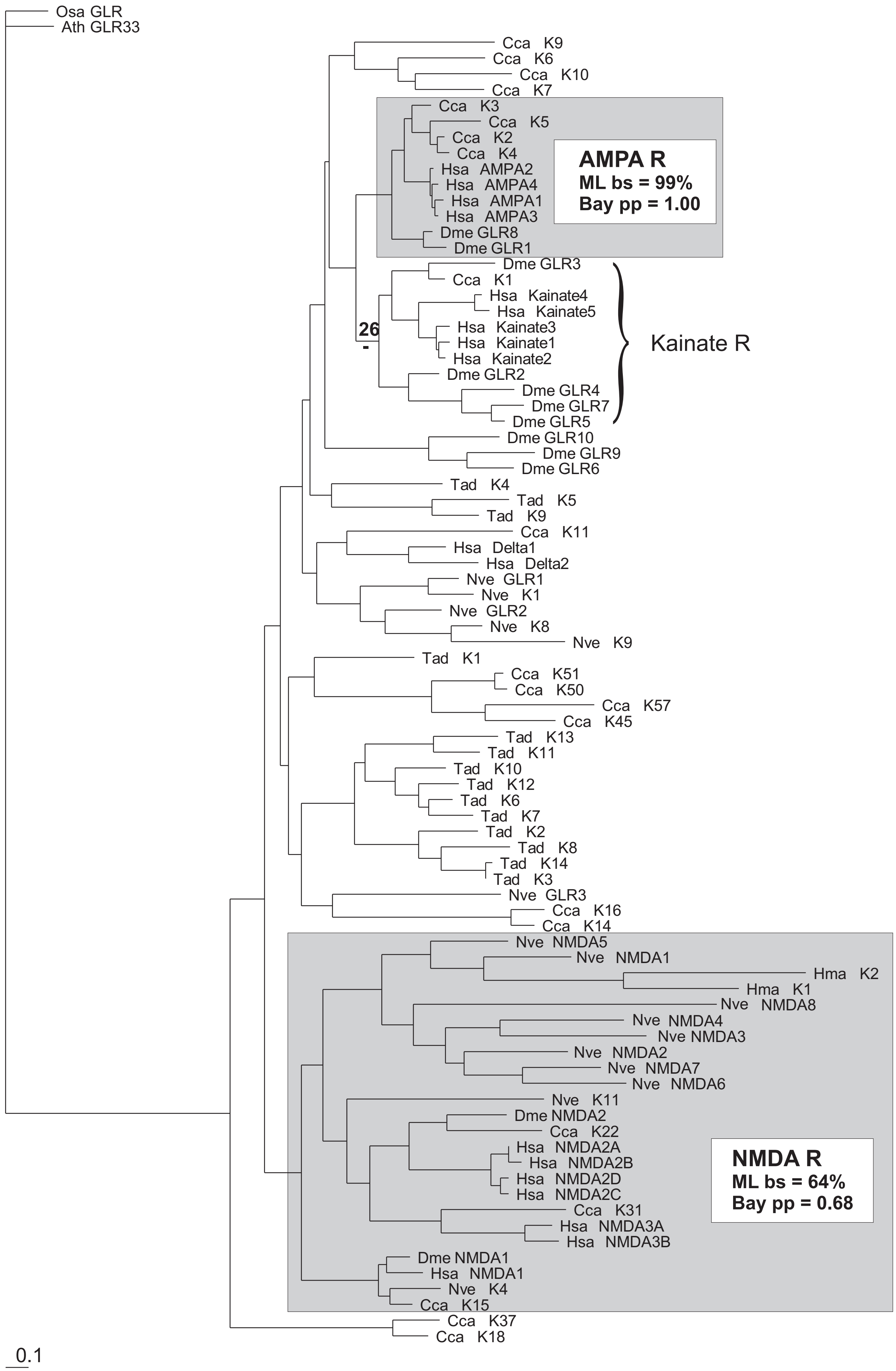

IP3 Receptor

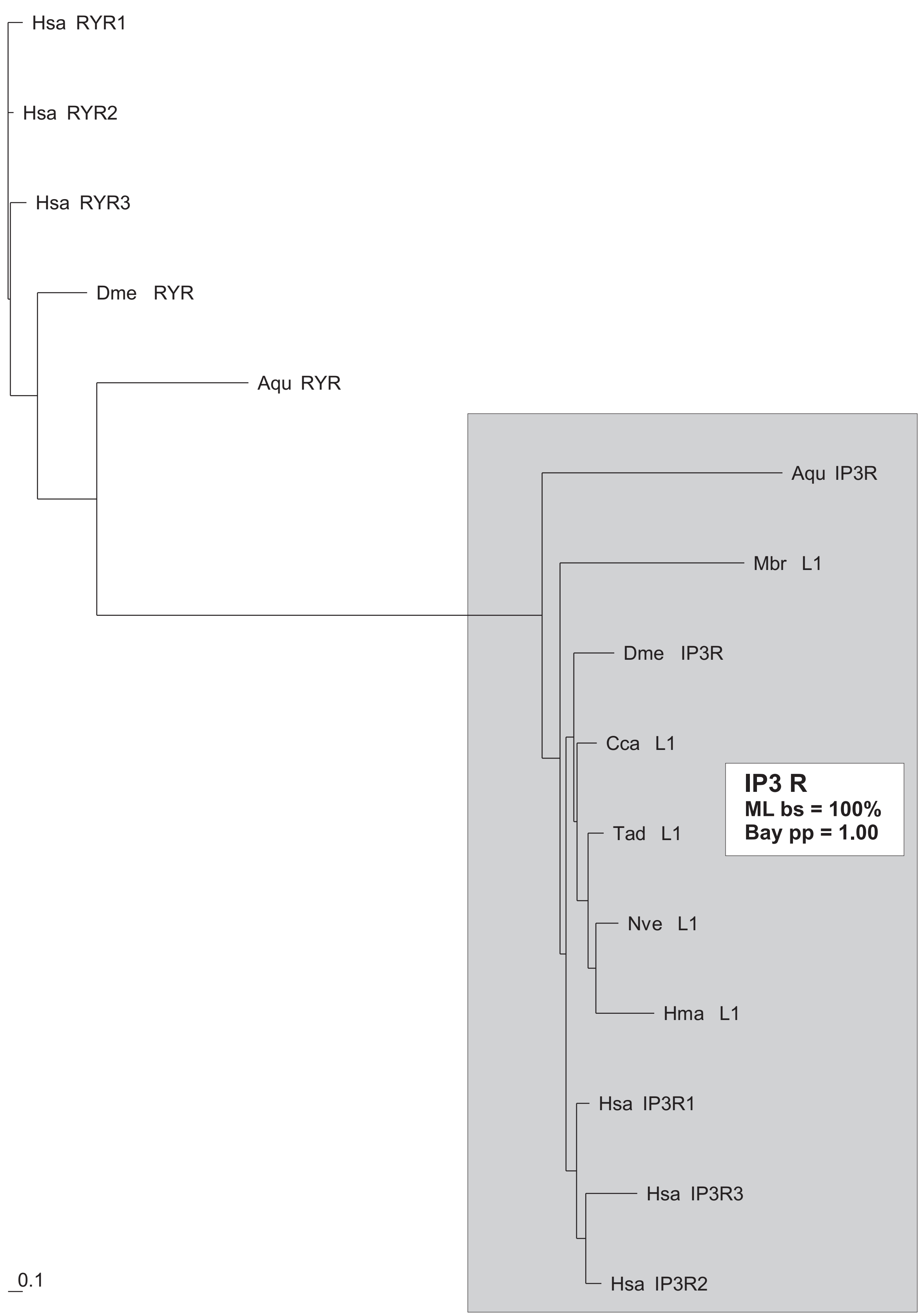

Metabotropic Glutamate Receptors

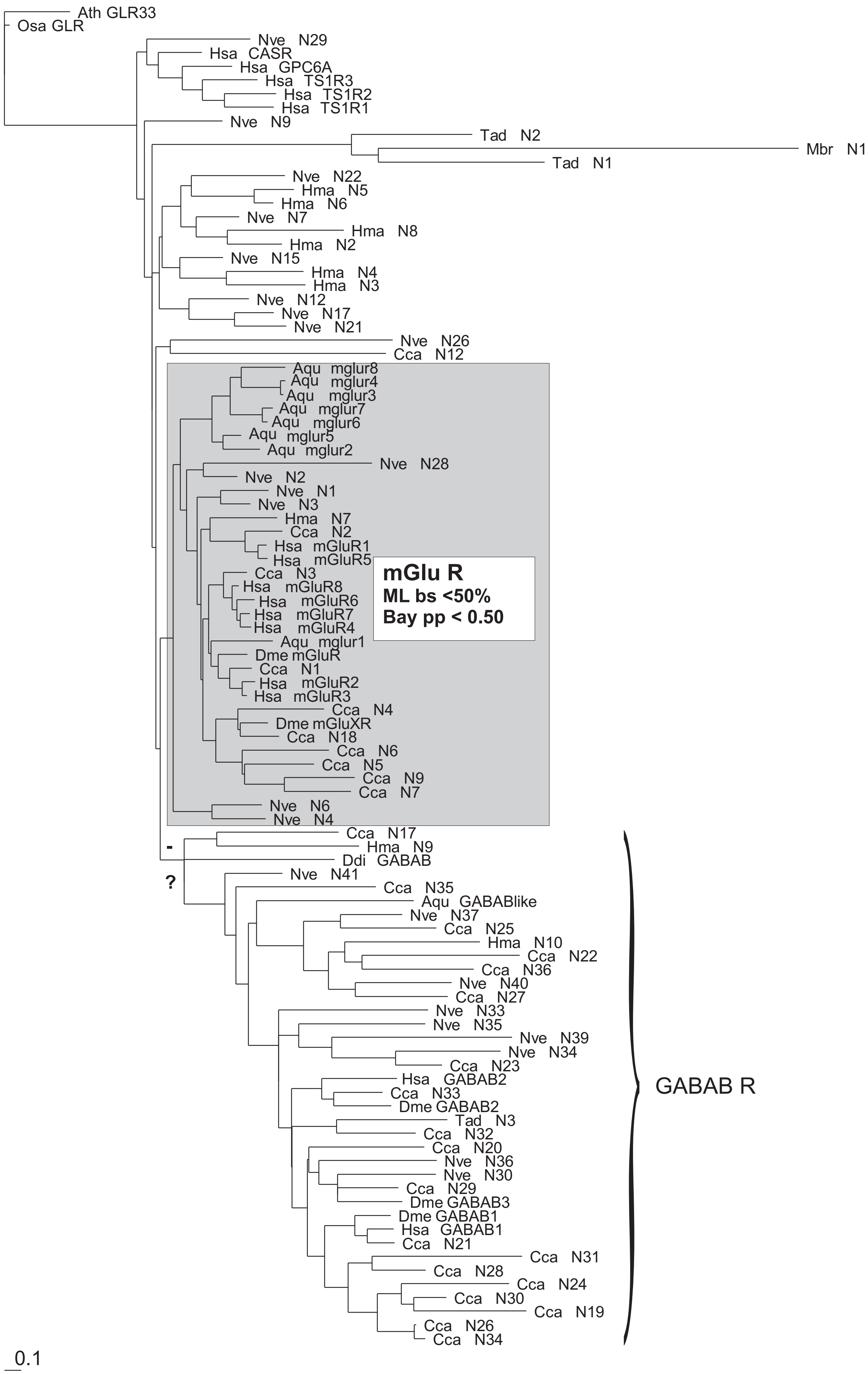

Neuroligin

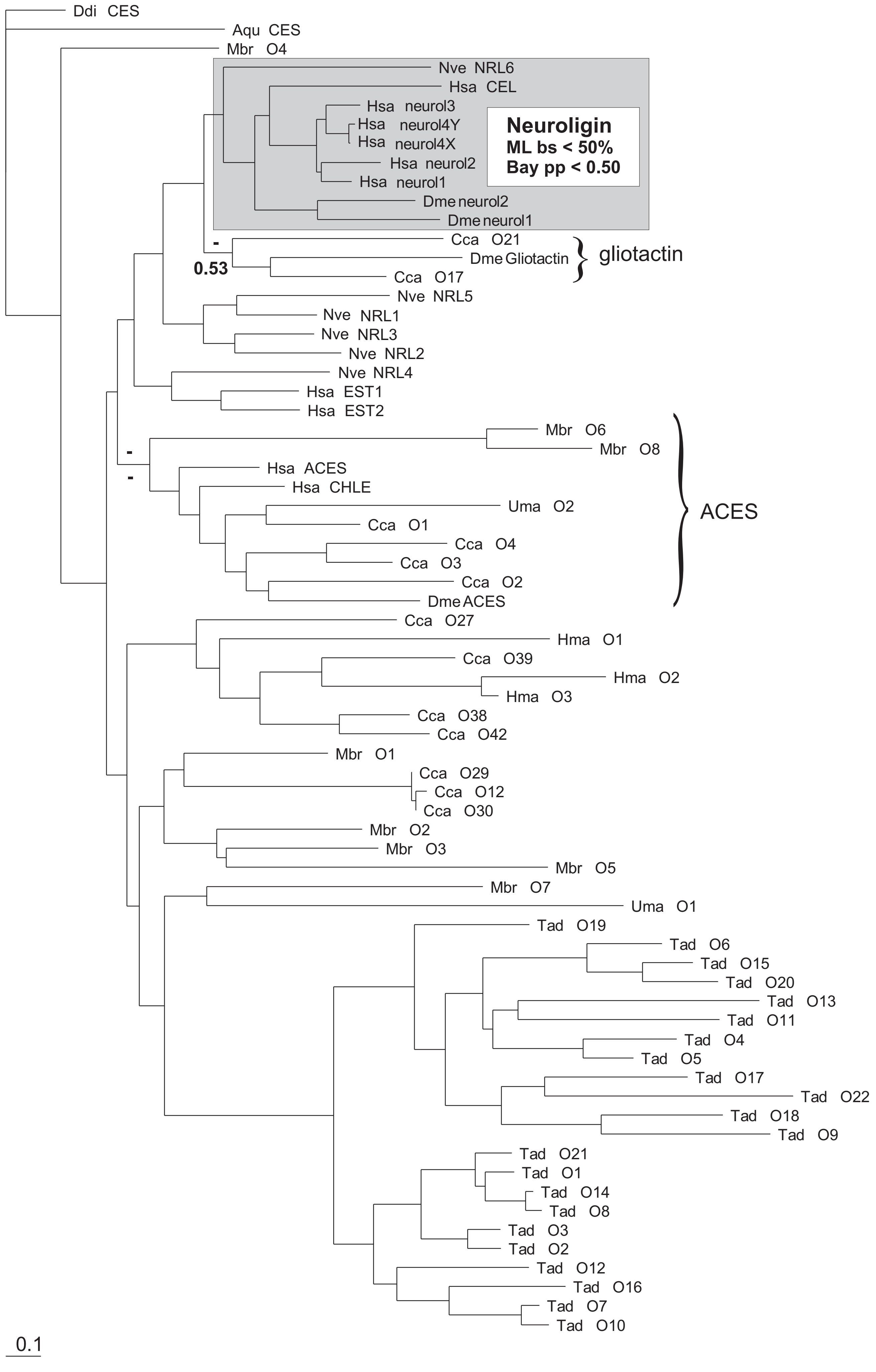

Ddi CES
Aqu CES
Mbr O4
Nve NRL6
Hsa CEL
Hsa neurol3
Hsa neurol4Y
Hsa neurol4X
Hsa neurol2
Hsa neurol1
Dme neurol2
Dme neurol1
Neuroligin
ML bs < 50%
Bay pp < 0.50
Cca O21
Dme Gliotactin
Cca O17
gliotactin
-
0.53
Nve NRL5
Nve NRL1
Nve NRL3
Nve NRL2
Nve NRL4
Hsa EST1
Hsa EST2
Mbr O6
Mbr O8
-
-
Hsa ACES
Hsa CHLE
Uma O2
Cca O1
Cca O4
Cca O3
Cca O2
Dme ACES
ACES
Cca O27
Hma O1
Cca O39
Hma O2
Hma O3
Cca O38
Cca O42
Mbr O1
Cca O29
Cca O12
Cca O30
Mbr O2
Mbr O3
Mbr O5
Mbr O7
Uma O1
Tad O19
Tad O6
Tad O15
Tad O20
Tad O13
Tad O11
Tad O4
Tad O5
Tad O17
Tad O22
Tad O18
Tad O9
Tad O21
Tad O1
Tad O14
Tad O8
Tad O3
Tad O2
Tad O12
Tad O16
Tad O7
Tad O10
0.1

NOS partition A

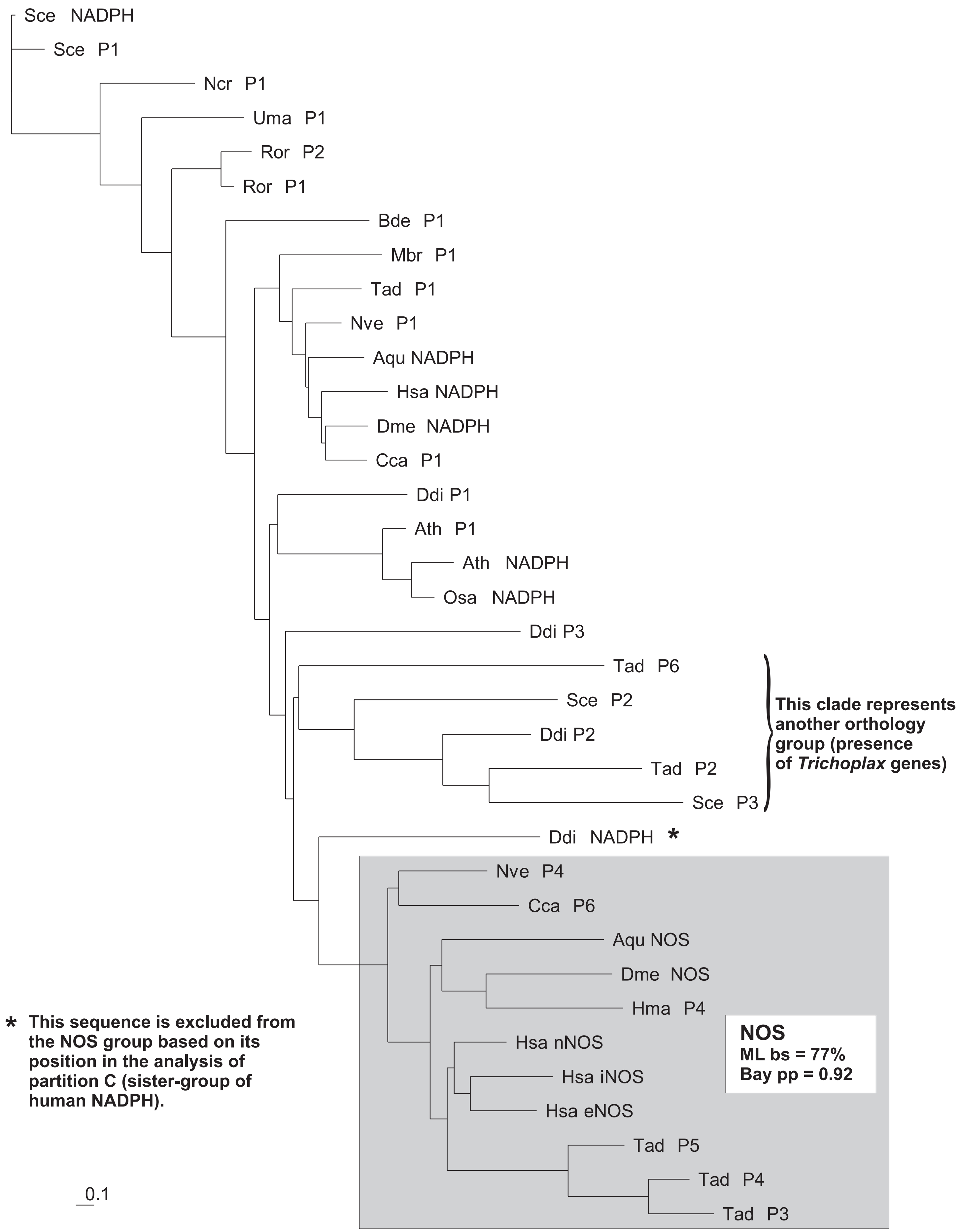

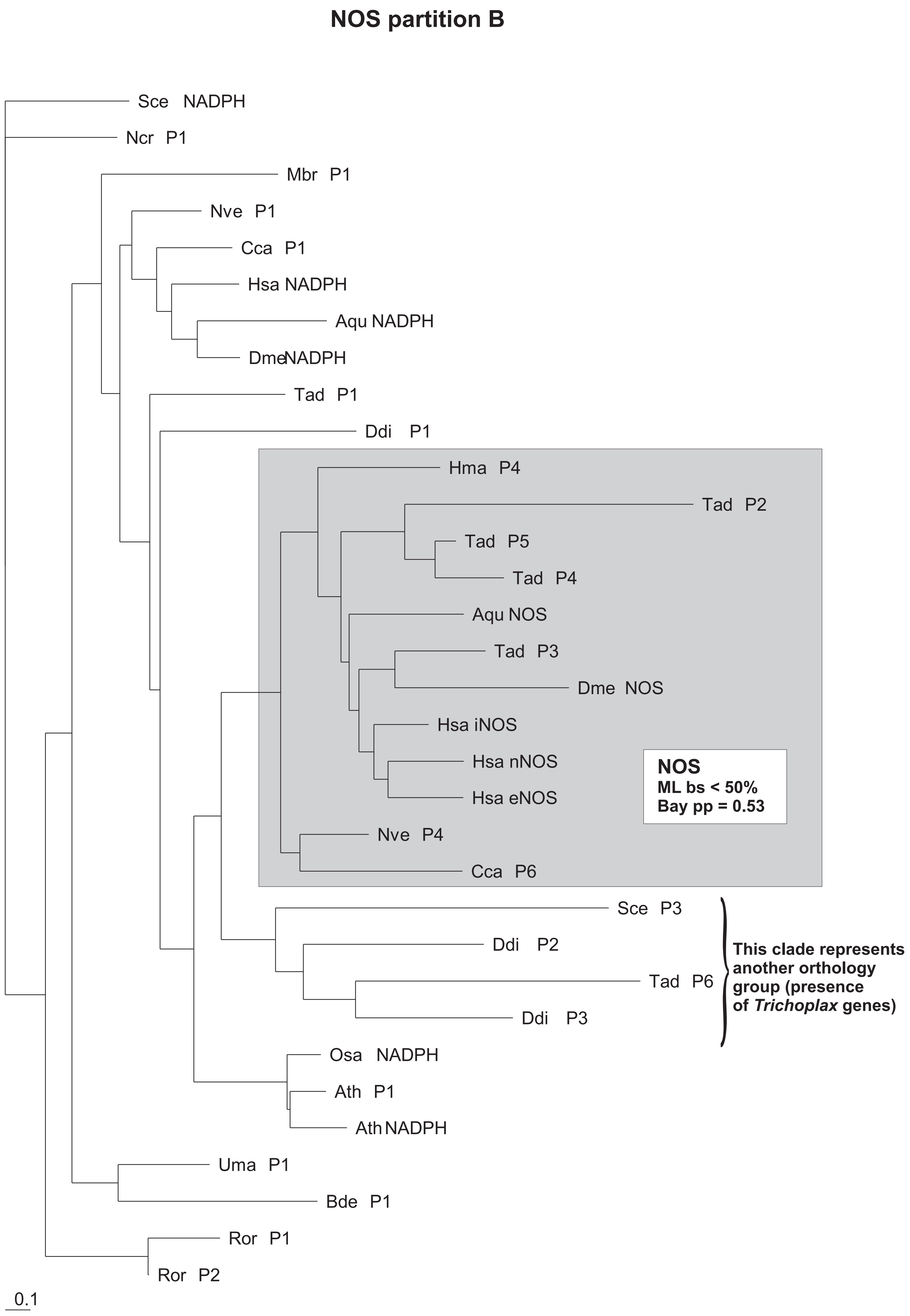
NOS partition B
Sce NADPH
Ncr P1
Mbr P1
Nve P1
Cca P1
Hsa NADPH
Aqu NADPH
DmeNADPH
Tad P1
Ddi P1
Hma P4
Tad P2
Tad P5
Tad P4
Aqu NOS
Tad P3
Dme NOS
Hsa iNOS
Hsa nNOS
Hsa eNOS
NOS
ML bs < 50%
Bay pp = 0.53
Nve P4
Cca P6
Sce P3
Ddi P2
Tad P6
Ddi P3
This clade represents another orthology group (presence of *Trichoplax* genes)
Osa NADPH
Ath P1
Ath NADPH
Uma P1
Bde P1
Ror P1
Ror P2
0.1

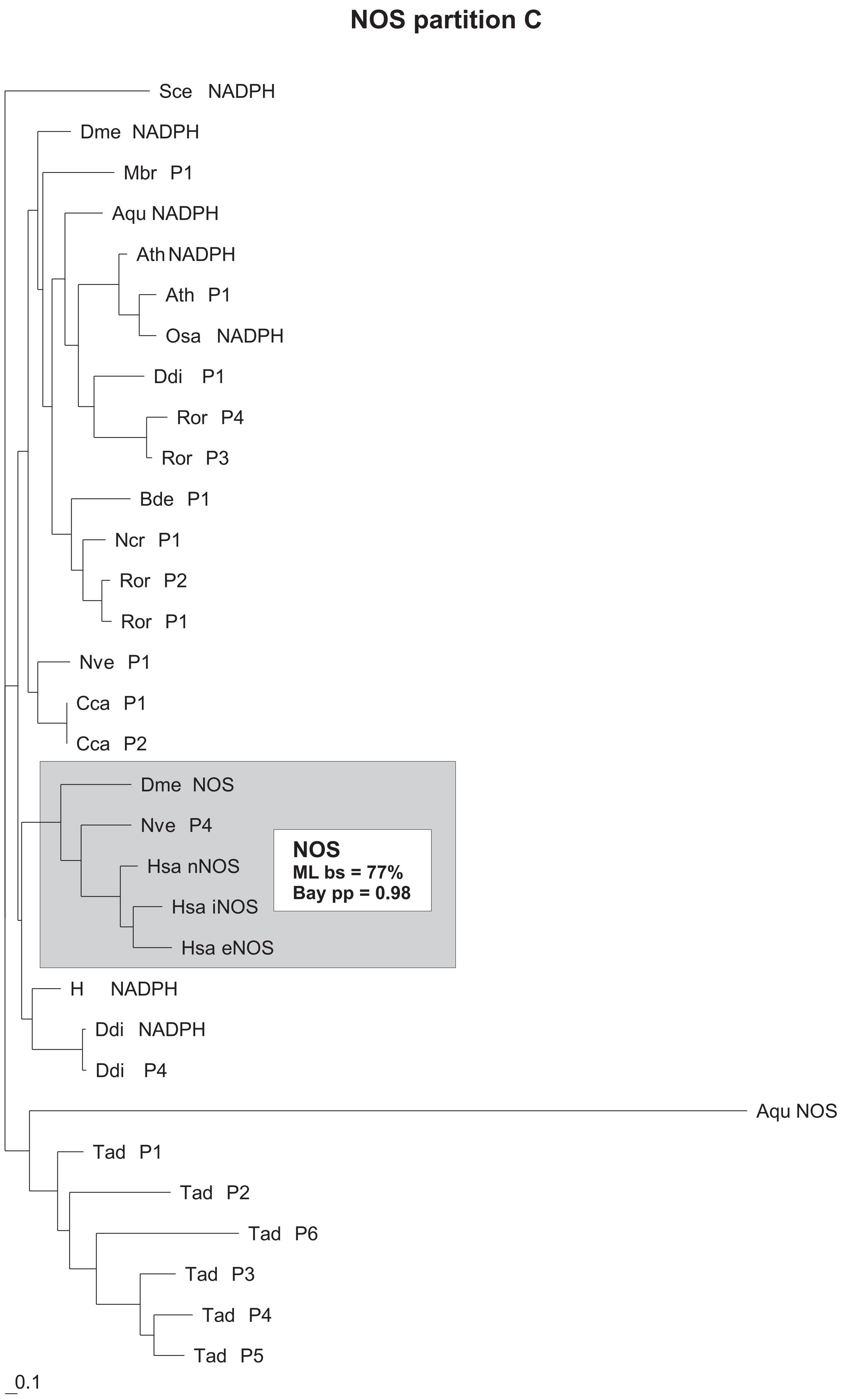
NOS partition C
Sce NADPH
Dme NADPH
Mbr P1
Aqu NADPH
Ath NADPH
Ath P1
Osa NADPH
Ddi P1
Ror P4
Ror P3
Bde P1
Ncr P1
Ror P2
Ror P1
Nve P1
Cca P1
Cca P2
Dme NOS
Nve P4
Hsa nNOS
Hsa iNOS
Hsa eNOS
NOS
ML bs = 77%
Bay pp = 0.98
H NADPH
Ddi NADPH
Ddi P4
Aqu NOS
Tad P1
Tad P2
Tad P6
Tad P3
Tad P4
Tad P5
0.1

PDZ domain

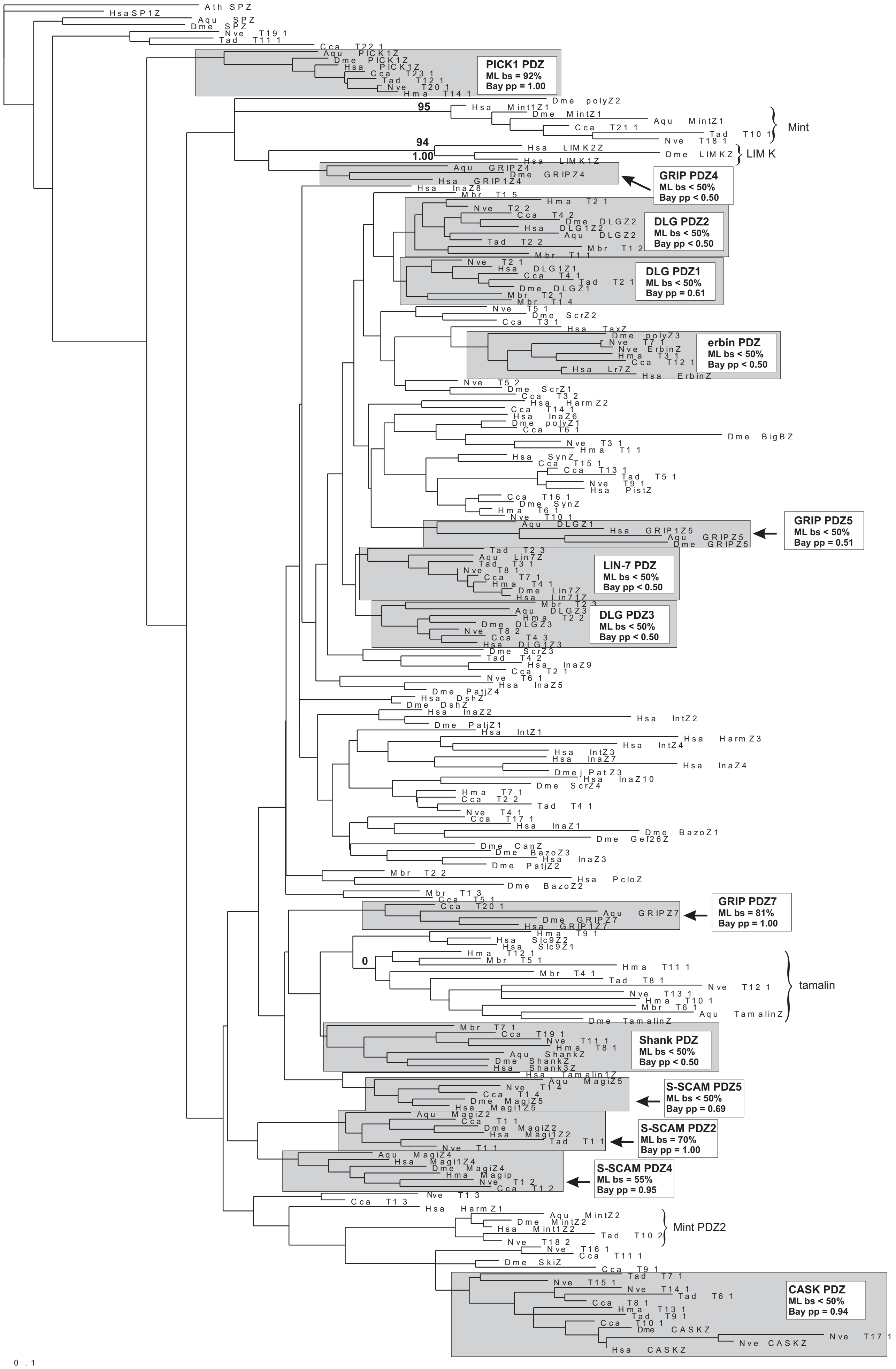
PICK1 PDZ
ML bs = 92%
Bay pp = 1.00
95
Mint
94
1.00
LIM K
GRIP PDZ4
ML bs < 50%
Bay pp < 0.50
DLG PDZ2
ML bs < 50%
Bay pp < 0.50
DLG PDZ1
ML bs < 50%
Bay pp = 0.61
erbin PDZ
ML bs < 50%
Bay pp < 0.50
GRIP PDZ5
ML bs < 50%
Bay pp = 0.51
LIN-7 PDZ
ML bs < 50%
Bay pp < 0.50
DLG PDZ3
ML bs < 50%
Bay pp < 0.50
GRIP PDZ7
ML bs = 81%
Bay pp = 1.00
0
tamalin
Shank PDZ
ML bs < 50%
Bay pp < 0.50
S-SCAM PDZ5
ML bs < 50%
Bay pp = 0.69
S-SCAM PDZ2
ML bs = 70%
Bay pp = 1.00
S-SCAM PDZ4
ML bs = 55%
Bay pp = 0.95
Mint PDZ2
CASK PDZ
ML bs < 50%
Bay pp = 0.94
0.1

Protein Kinase C

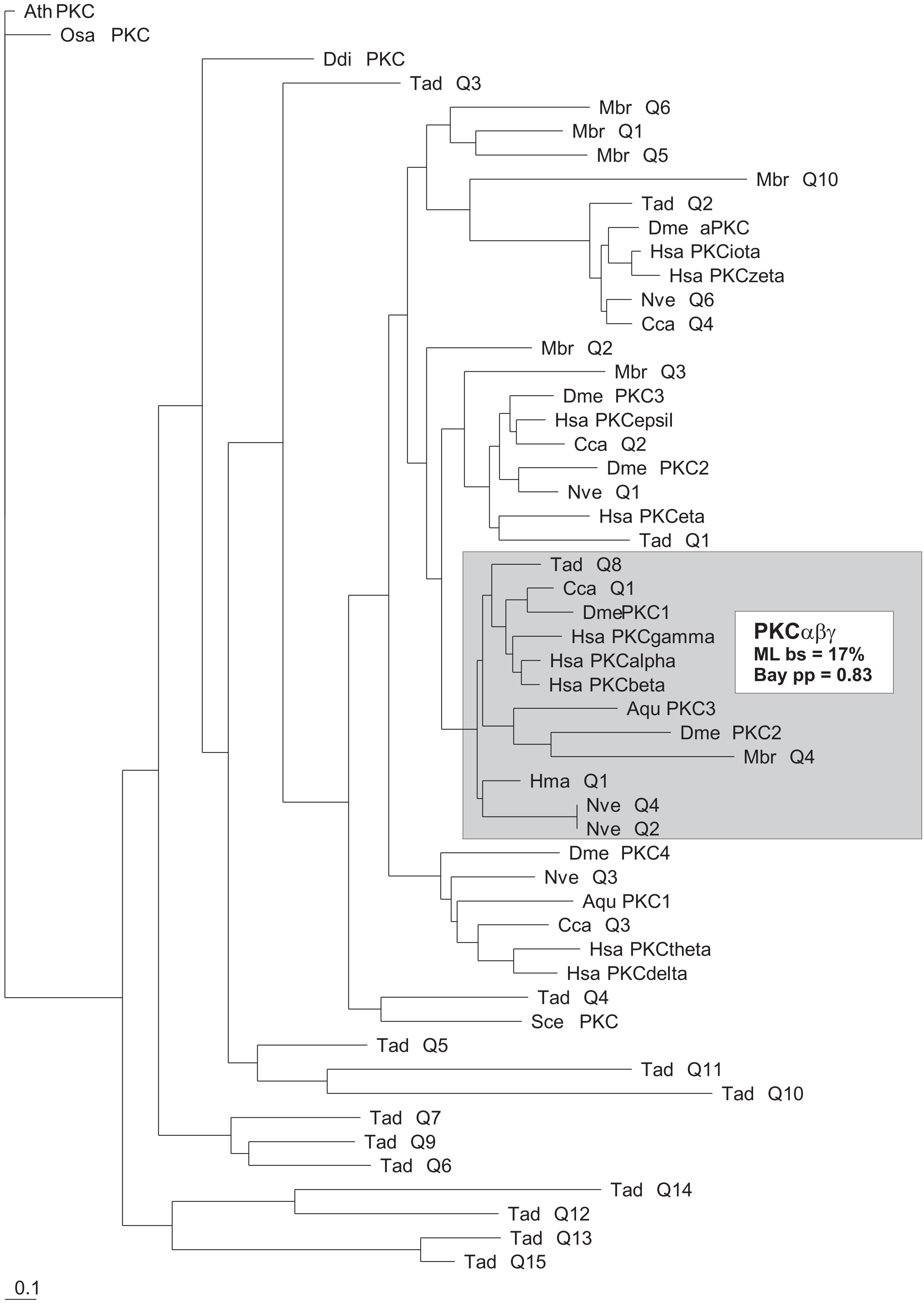

PMCA

PMCA has no similarity to any other protein.

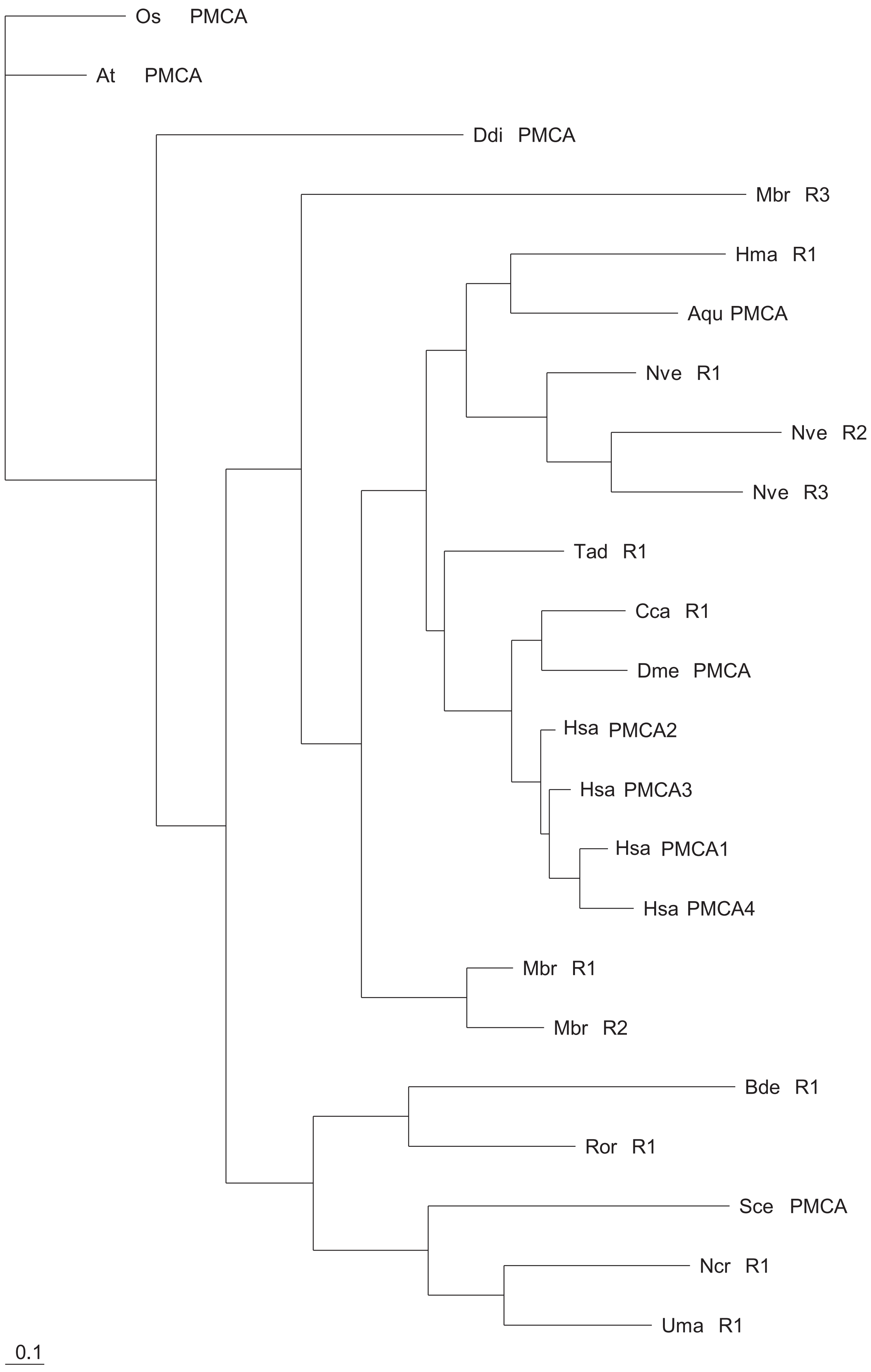

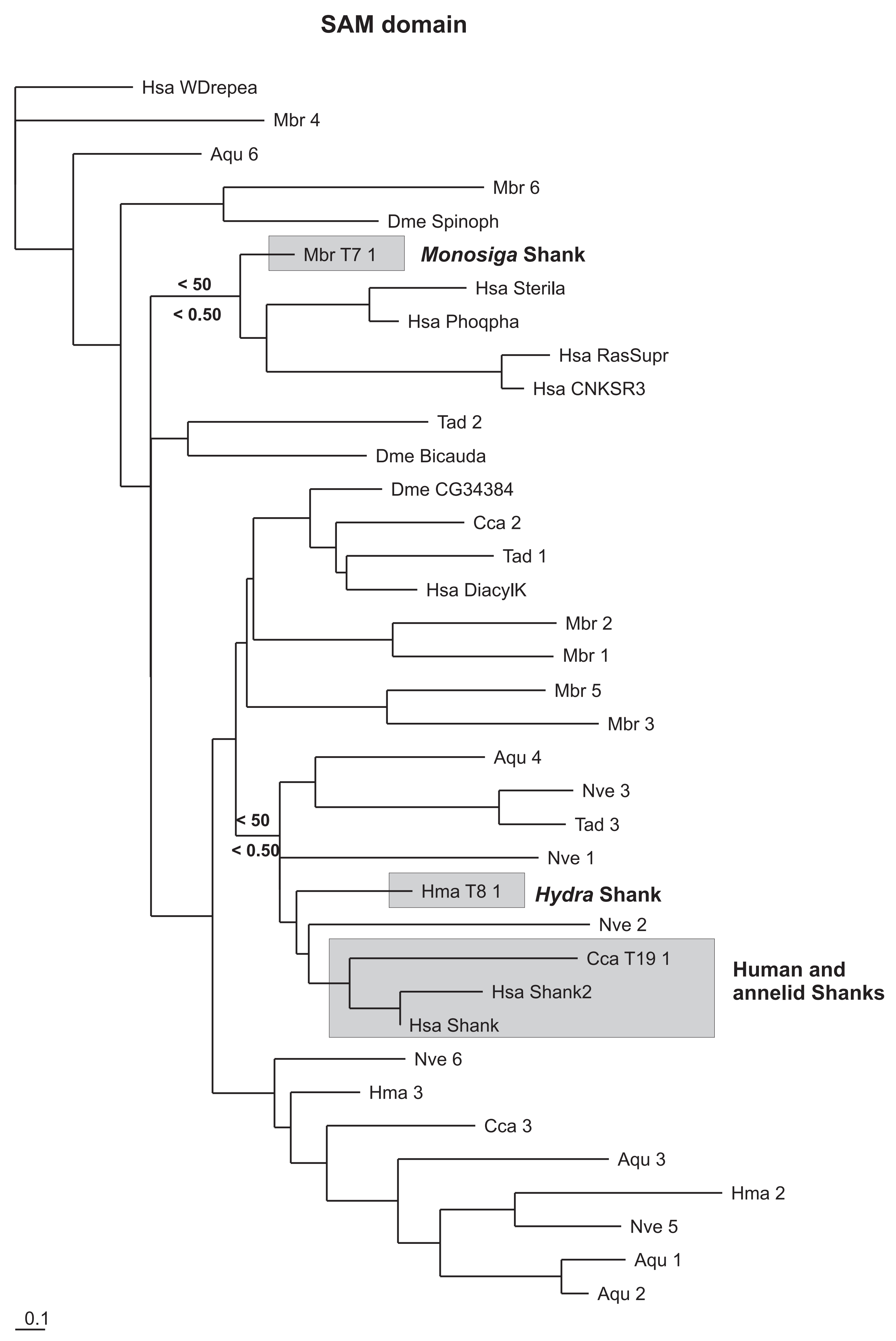
SAM domain
Hsa WDrepea
Mbr 4
Aqu 6
Mbr 6
Dme Spinoph
Mbr T7 1
Monosiga Shank
< 50
< 0.50
Hsa Sterila
Hsa Phoqpha
Hsa RasSupr
Hsa CNKSR3
Tad 2
Dme Bicauda
Dme CG34384
Cca 2
Tad 1
Hsa DiacylK
Mbr 2
Mbr 1
Mbr 5
Mbr 3
Aqu 4
Nve 3
Tad 3
< 50
< 0.50
Nve 1
Hma T8 1
Hydra Shank
Nve 2
Cca T19 1
Hsa Shank2
Hsa Shank
Human and annelid Shanks
Nve 6
Hma 3
Cca 3
Aqu 3
Hma 2
Nve 5
Aqu 1
Aqu 2
0.1

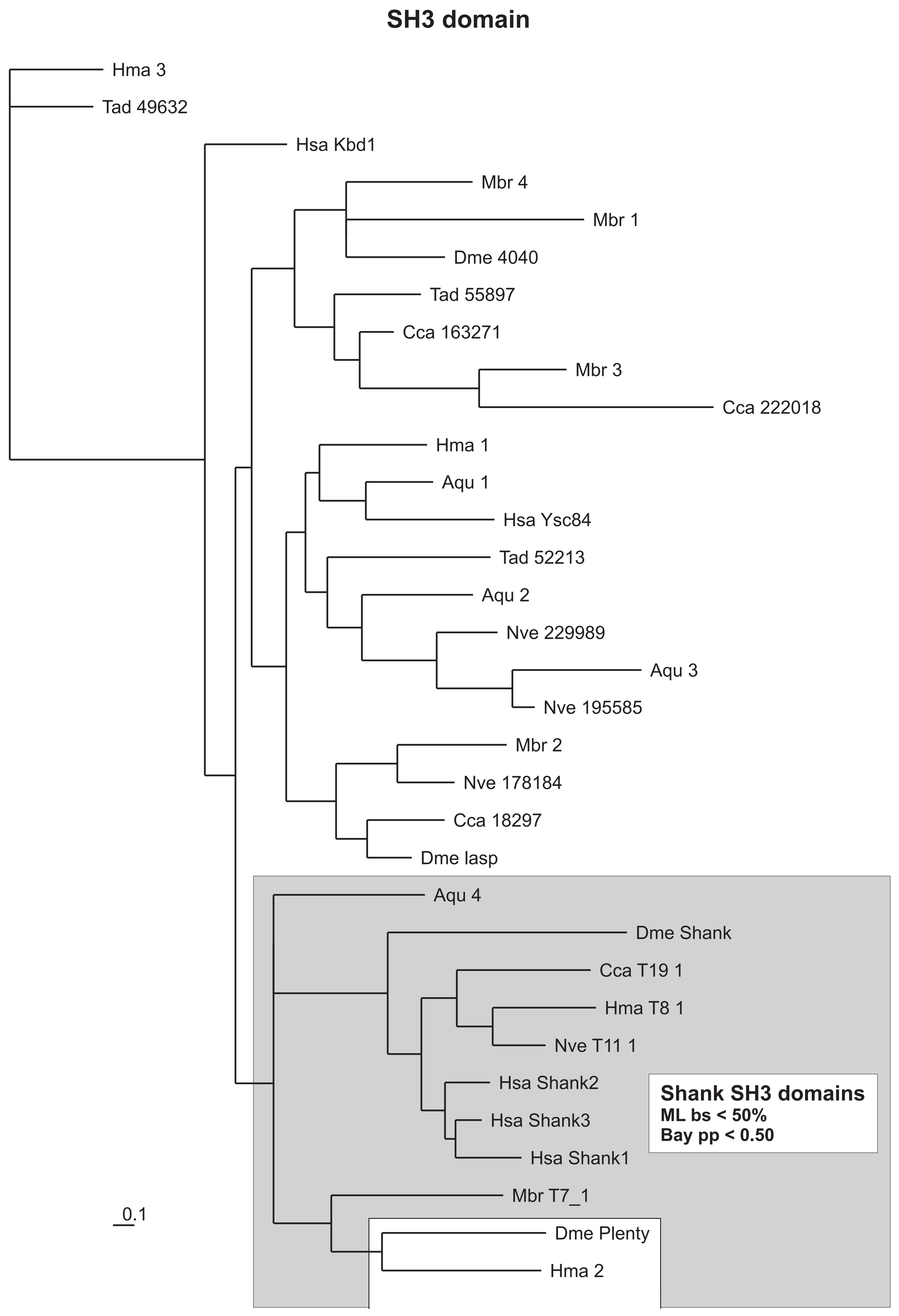

SH3 domain
Hma 3
Tad 49632
Hsa Kbd1
Mbr 4
Mbr 1
Dme 4040
Tad 55897
Cca 163271
Mbr 3
Cca 222018
Hma 1
Aqu 1
Hsa Ysc84
Tad 52213
Aqu 2
Nve 229989
Aqu 3
Nve 195585
Mbr 2
Nve 178184
Cca 18297
Dme lasp
Aqu 4
Dme Shank
Cca T19 1
Hma T8 1
Nve T11 1
Hsa Shank2
Hsa Shank3
Hsa Shank1
Shank SH3 domains
ML bs < 50%
Bay pp < 0.50
Mbr T7_1
Dme Plenty
Hma 2
0.1

K$^+$ channel Shaker

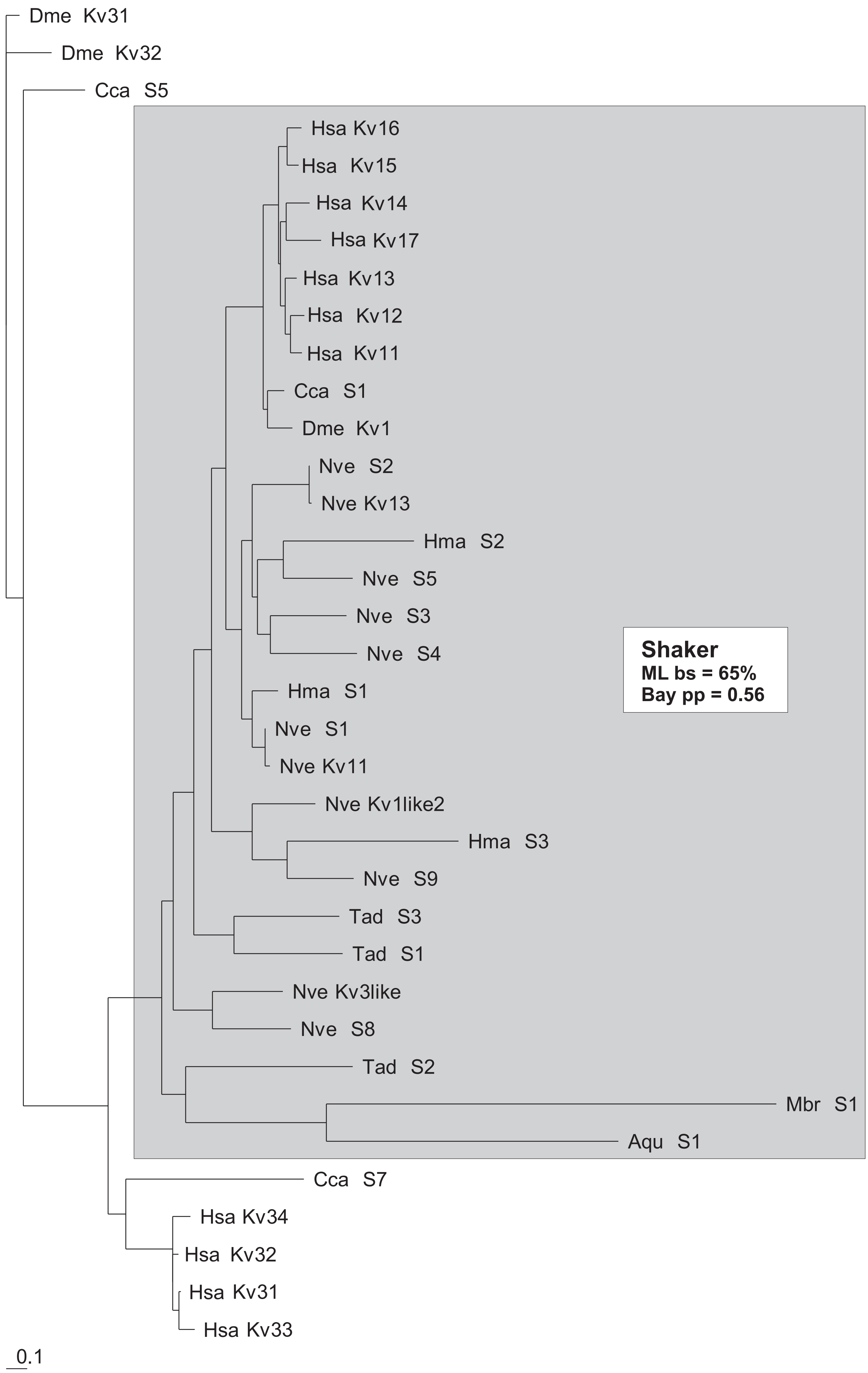

SPAR

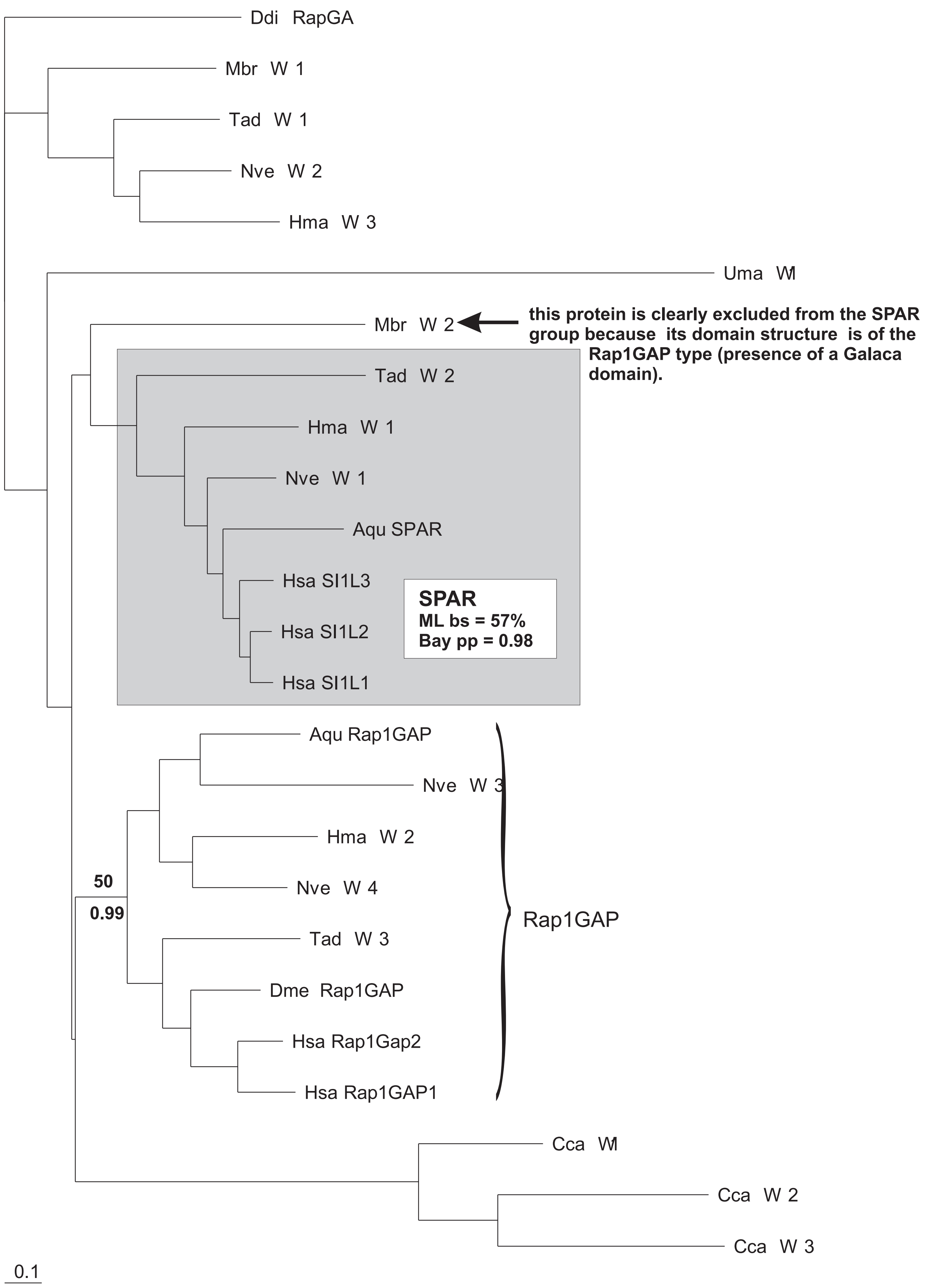
Ddi RapGA
Mbr W 1
Tad W 1
Nve W 2
Hma W 3
Uma W1
Mbr W 2
this protein is clearly excluded from the SPAR group because its domain structure is of the Rap1GAP type (presence of a Galaca domain).
Tad W 2
Hma W 1
Nve W 1
Aqu SPAR
Hsa SI1L3
Hsa SI1L2
Hsa SI1L1
SPAR
ML bs = 57%
Bay pp = 0.98
Aqu Rap1GAP
Nve W 3
Hma W 2
Nve W 4
50
0.99
Tad W 3
Dme Rap1GAP
Hsa Rap1Gap2
Hsa Rap1GAP1
Rap1GAP
Cca W1
Cca W 2
Cca W 3
0.1

Stargazin

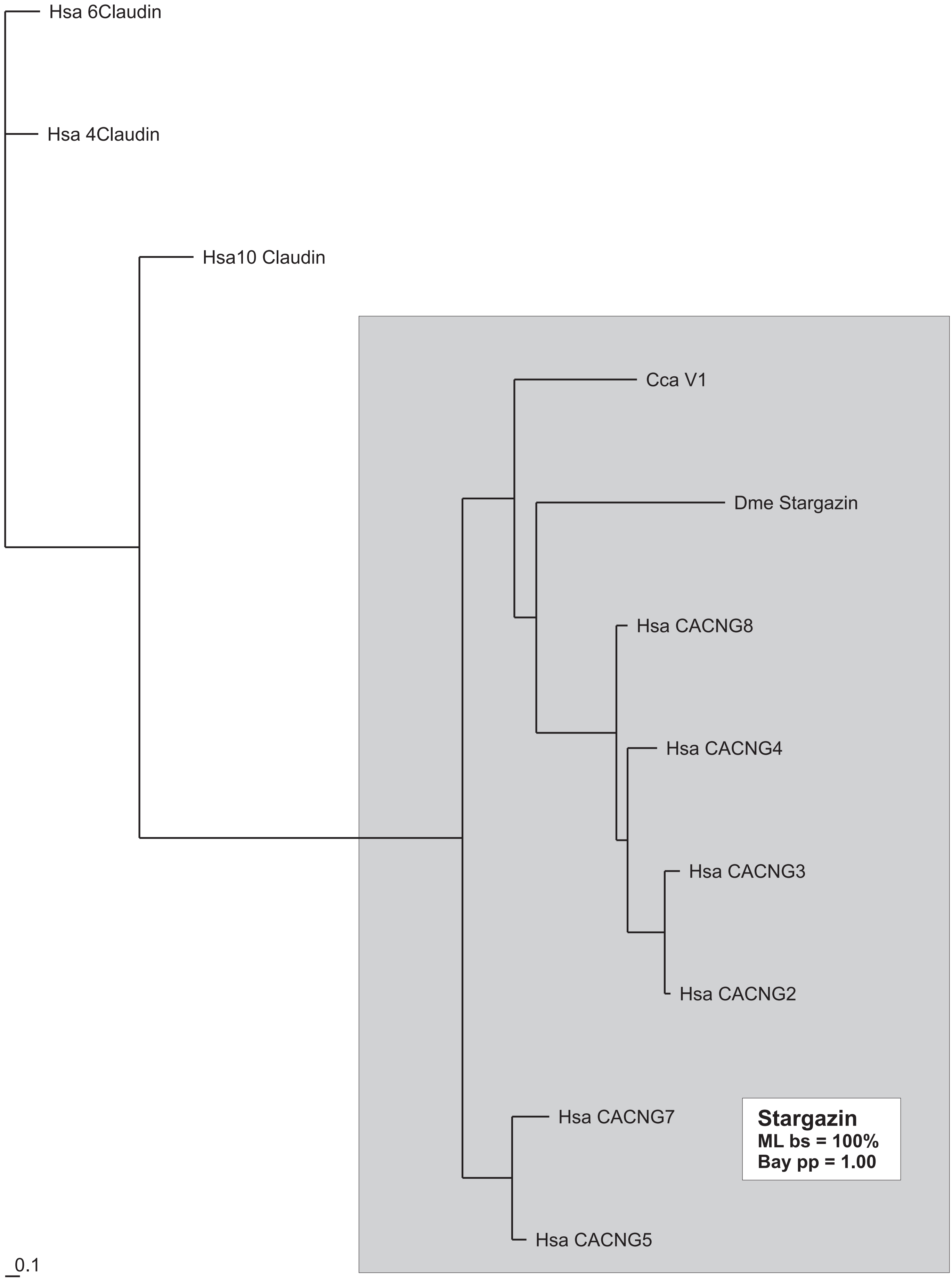

Hsa 6Claudin
Hsa 4Claudin
Hsa10 Claudin
Cca V1
Dme Stargazin
Hsa CACNG8
Hsa CACNG4
Hsa CACNG3
Hsa CACNG2
Hsa CACNG7
Hsa CACNG5
Stargazin
ML bs = 100%
Bay pp = 1.00
0.1

SynGAP

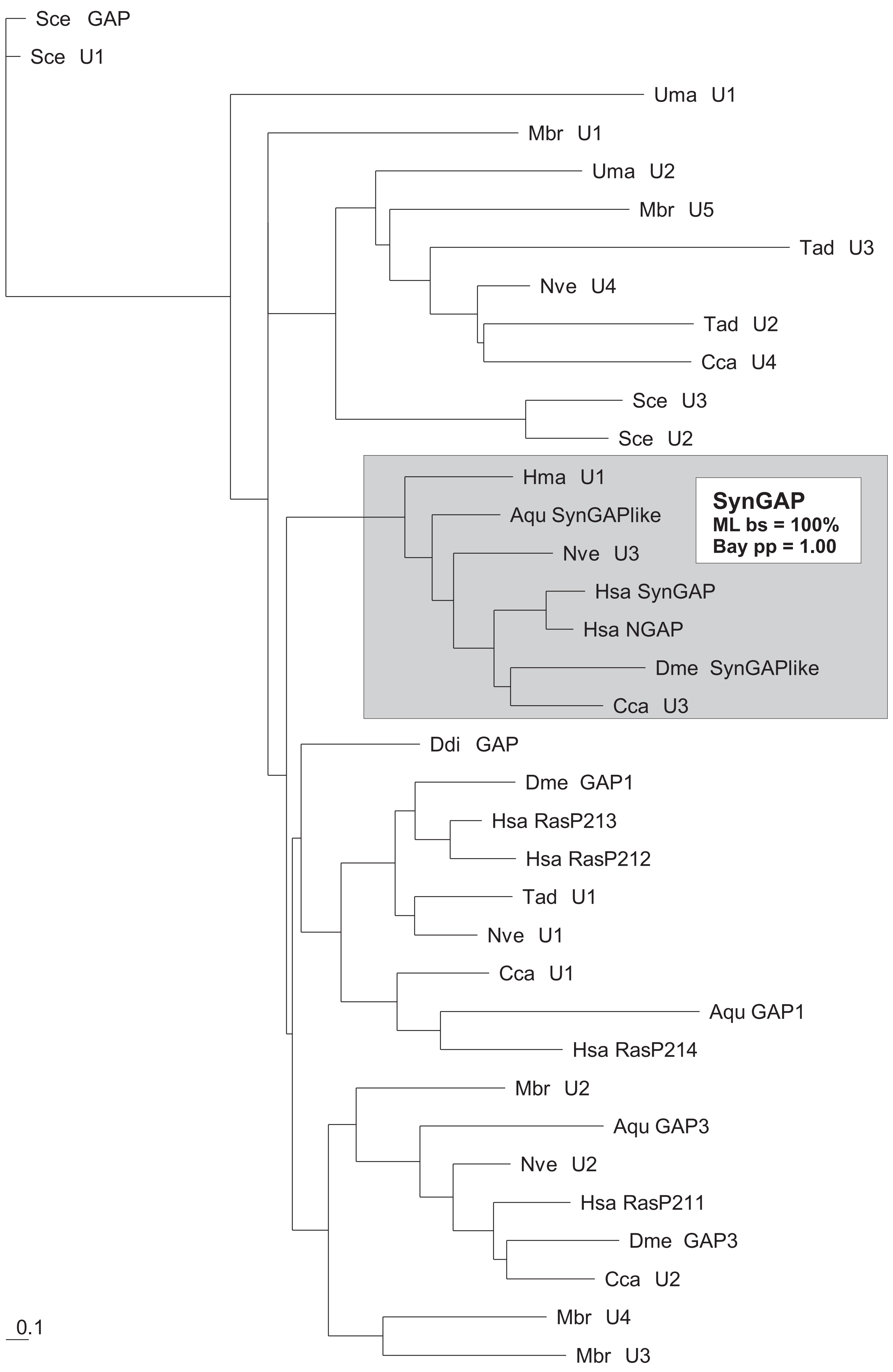
Sce GAP
Sce U1
Uma U1
Mbr U1
Uma U2
Mbr U5
Tad U3
Nve U4
Tad U2
Cca U4
Sce U3
Sce U2
Hma U1
Aqu SynGAPlike
Nve U3
Hsa SynGAP
Hsa NGAP
Dme SynGAPlike
Cca U3
SynGAP
ML bs = 100%
Bay pp = 1.00
Ddi GAP
Dme GAP1
Hsa RasP213
Hsa RasP212
Tad U1
Nve U1
Cca U1
Aqu GAP1
Hsa RasP214
Mbr U2
Aqu GAP3
Nve U2
Hsa RasP211
Dme GAP3
Cca U2
Mbr U4
Mbr U3
0.1
