# Supplementary material for: The backbone of the post-synaptic density originated in a unicellular ancestor of choanoflagellates and metazoans
Source: BMC Evol Biol. 2010 Feb 3;10:34. doi: 10.1186/1471-2148-10-34 (PMC2824662; doi:10.1186/1471-2148-10-34)
Supplement: Additional file 5 — Domain architectures of selected members of the post-synaptic gene families (in alphabetic order). Abbreviations used in displays for taxon names are as in Additional file 2. [file 1471-2148-10-34-S5.PDF]

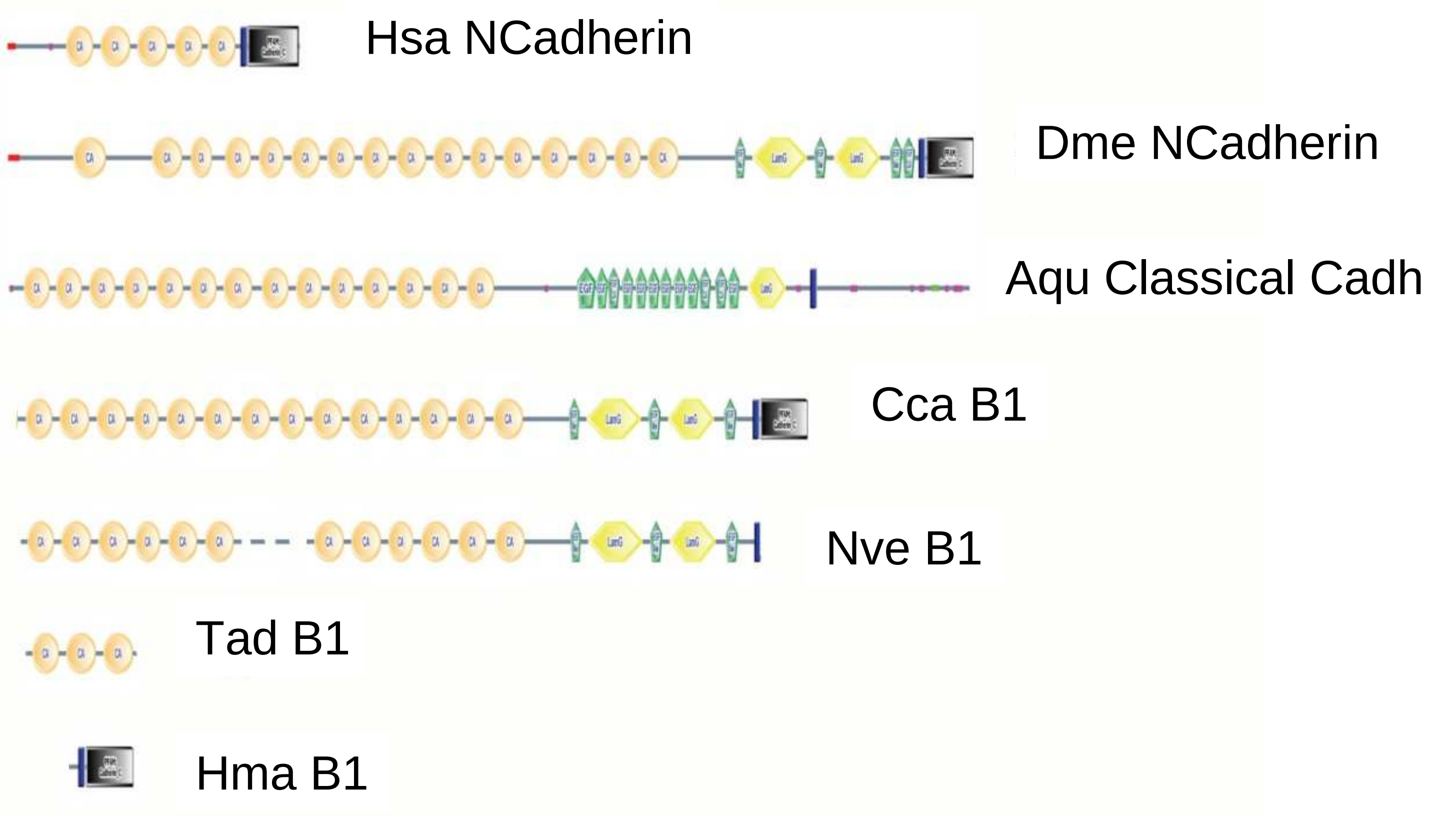
Hsa NCadherin
Dme NCadherin
Aqu Classical Cadh
Cca B1
Nve B1
Tad B1
Hma B1

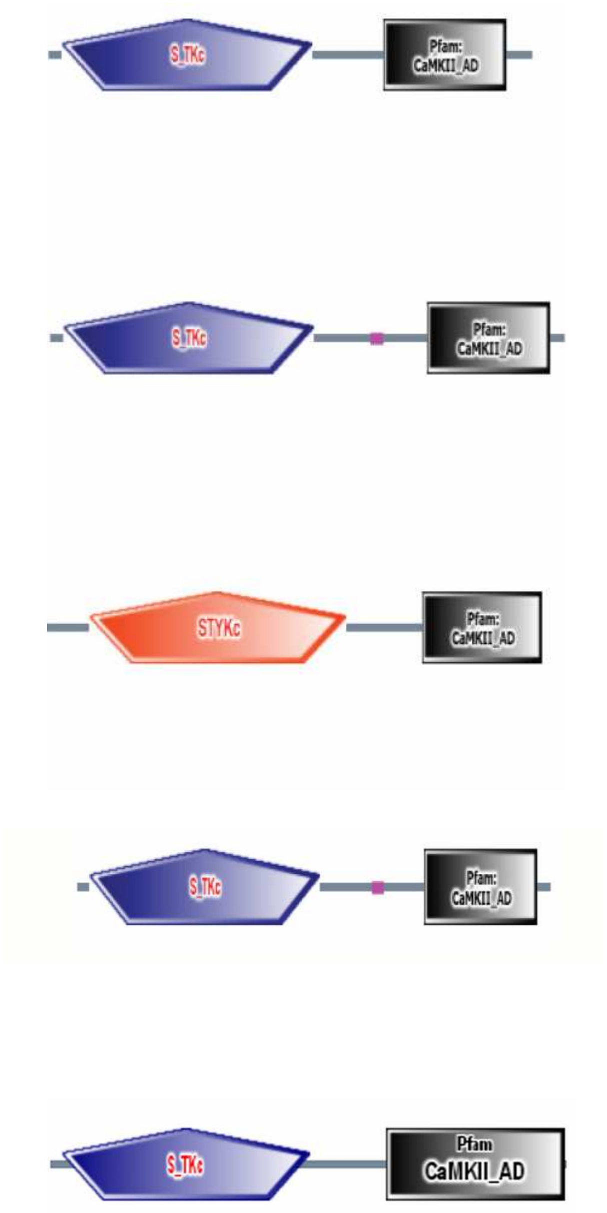

Hsa CamKIIdel

Dme CamKII

Aqu CamKIIlik

Tad C1

Mbr C1

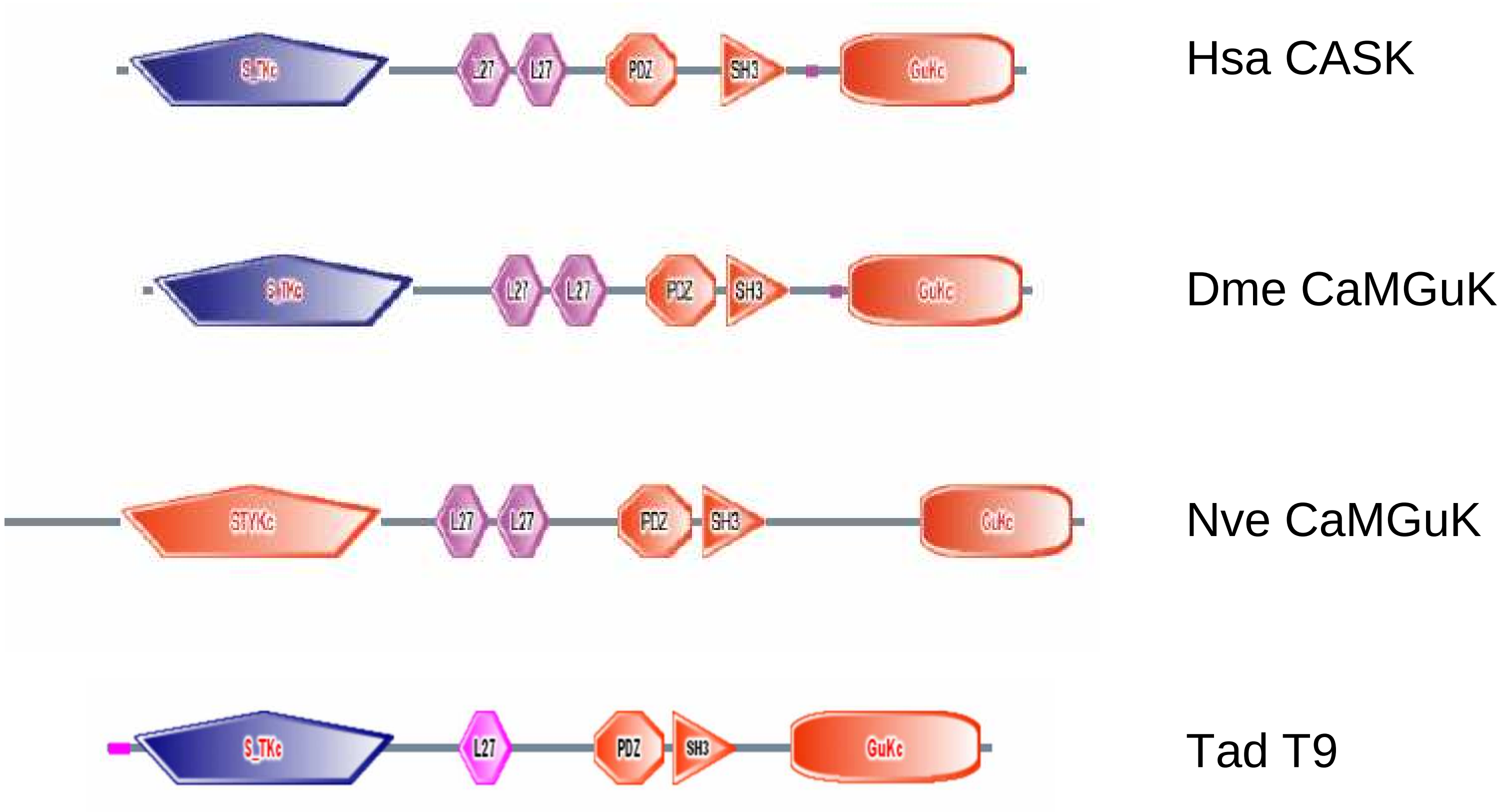
S_TKc
L27
L27
PDZ
SH3
GuKc
Hsa CASK
Dme CaMGuK
STYKc
Nve CaMGuK
Tad T9

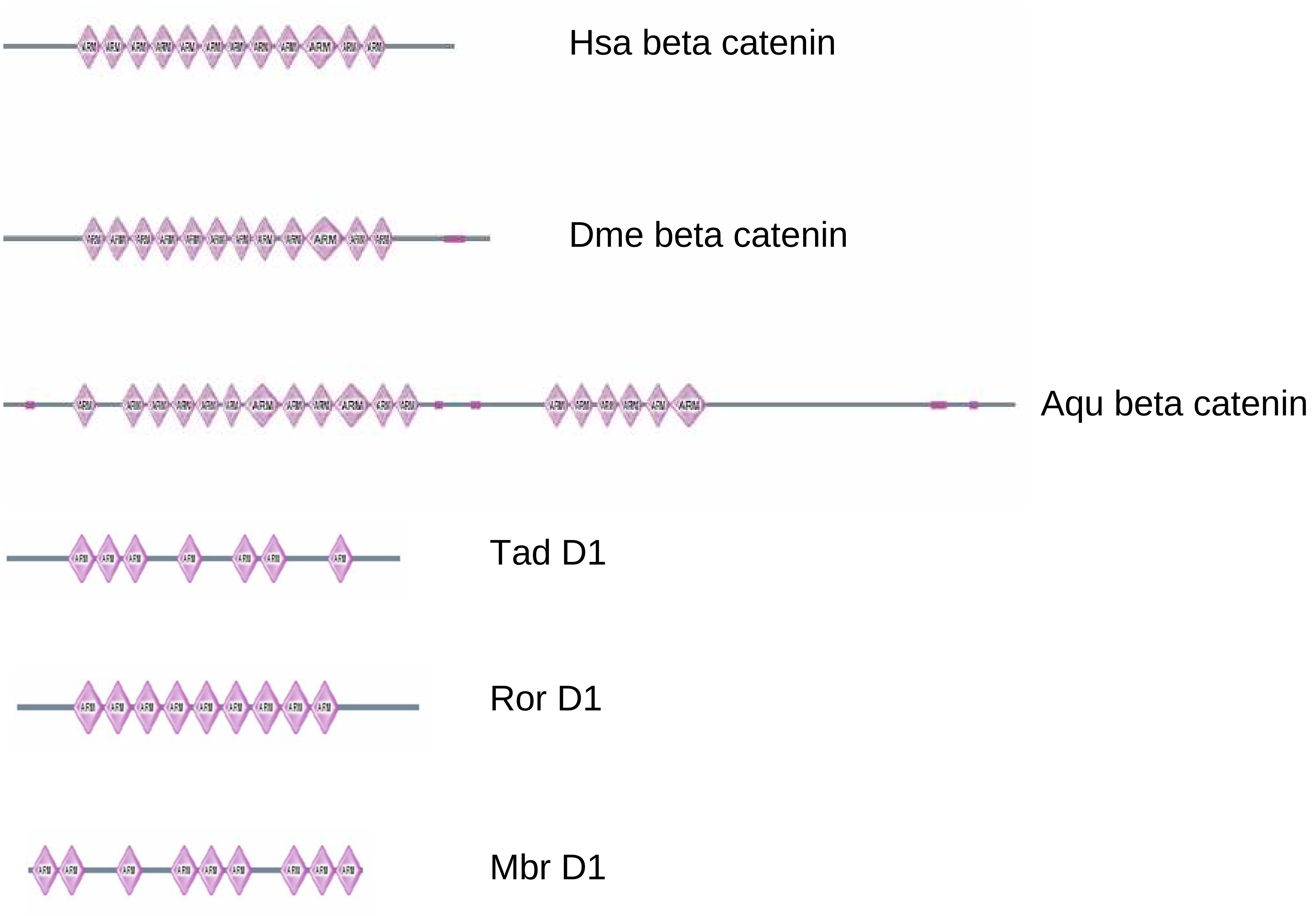
Hsa beta catenin
Dme beta catenin
Aqu beta catenin
Tad D1
Ror D1
Mbr D1

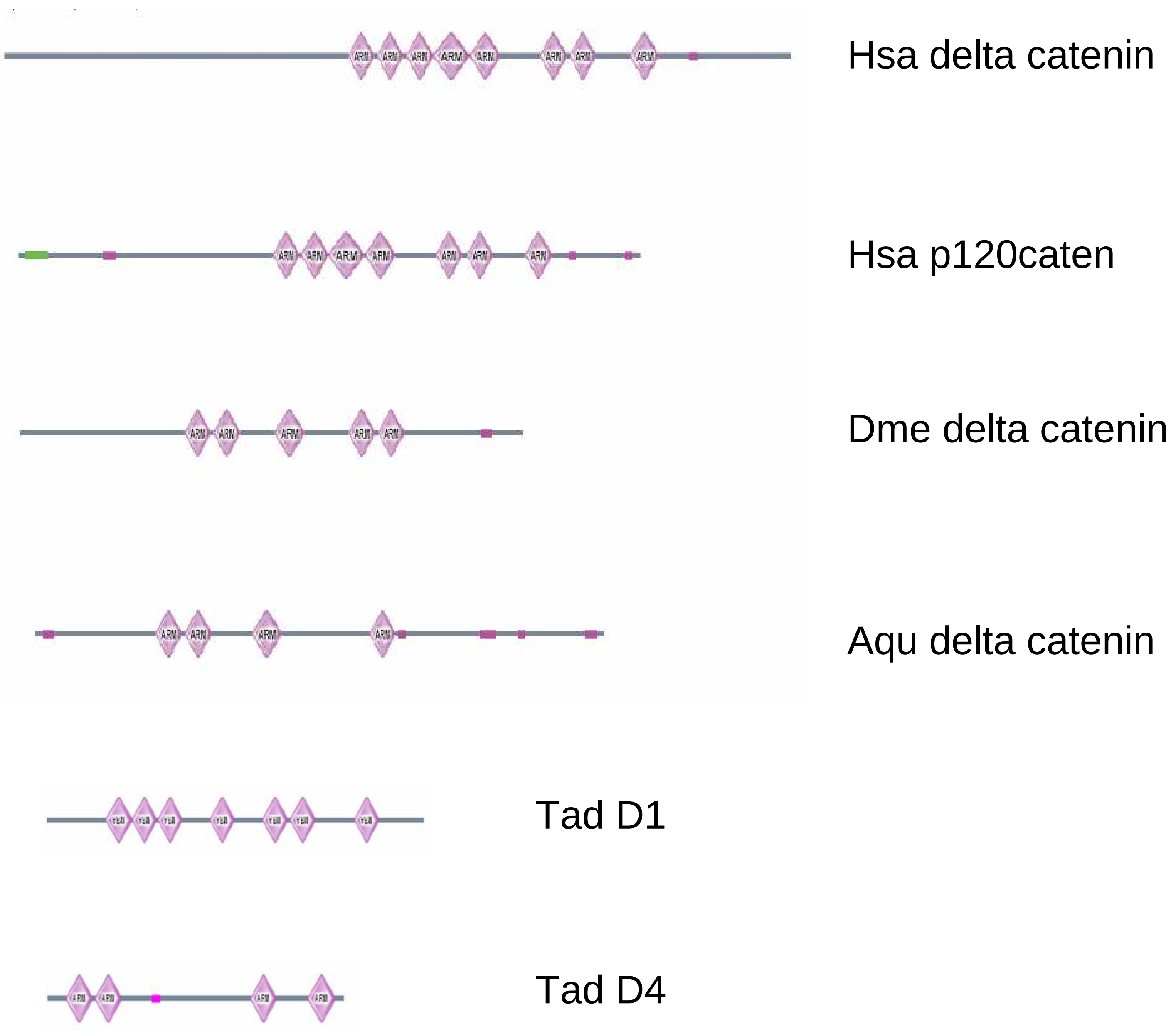
Hsa delta catenin
Hsa p120caten
Dme delta catenin
Aqu delta catenin
Tad D1
Tad D4

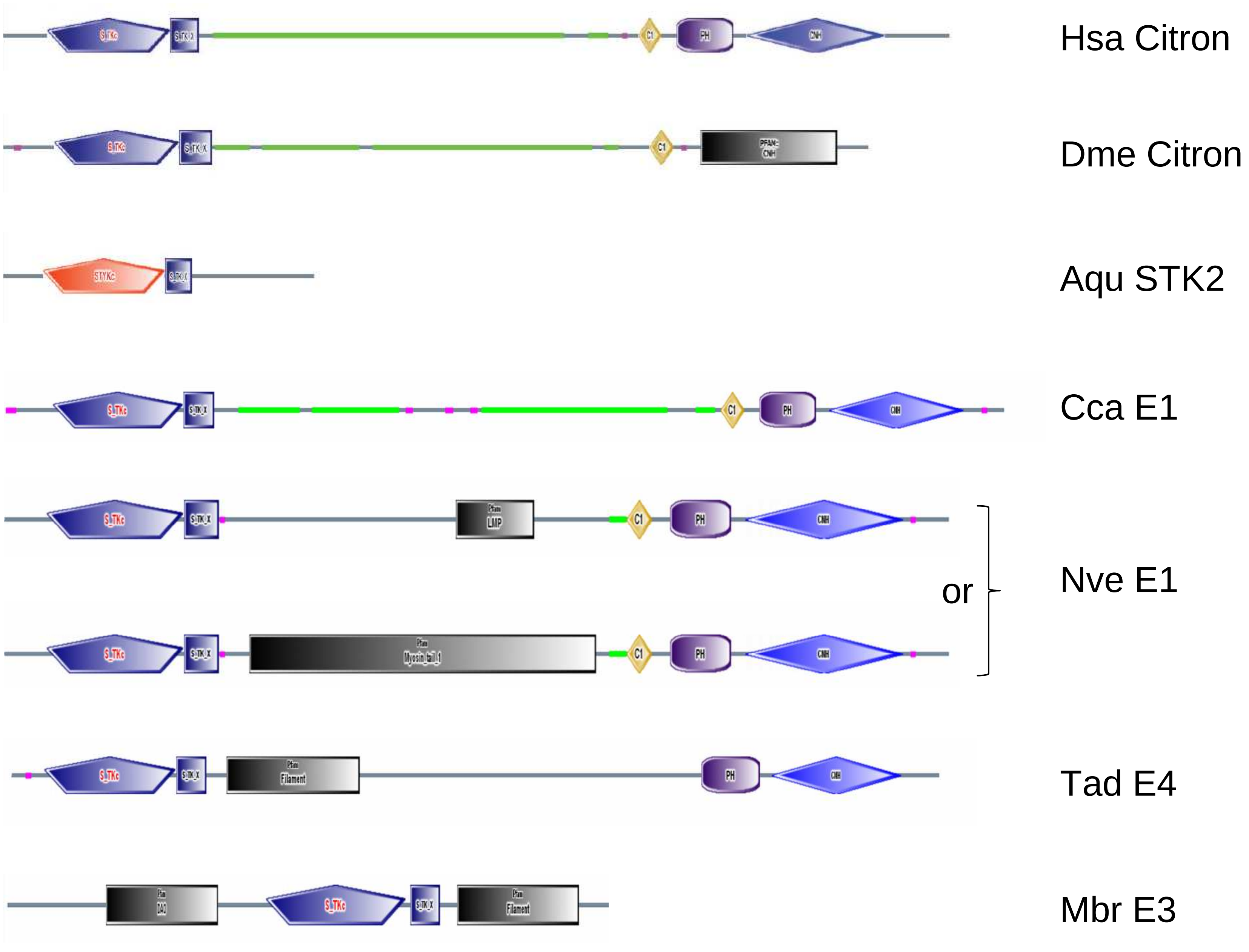
Hsa Citron
Dme Citron
Aqu STK2
Cca E1
or
Nve E1
Tad E4
Mbr E3

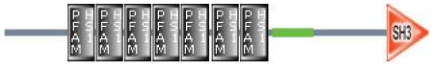

Hsa Cortactin

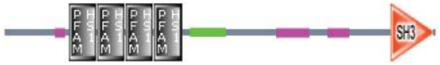

Dme Cortactin

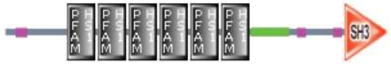

Aqu Cortactin

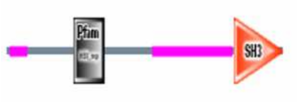

Mbr F1

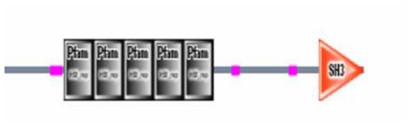

Tad F1

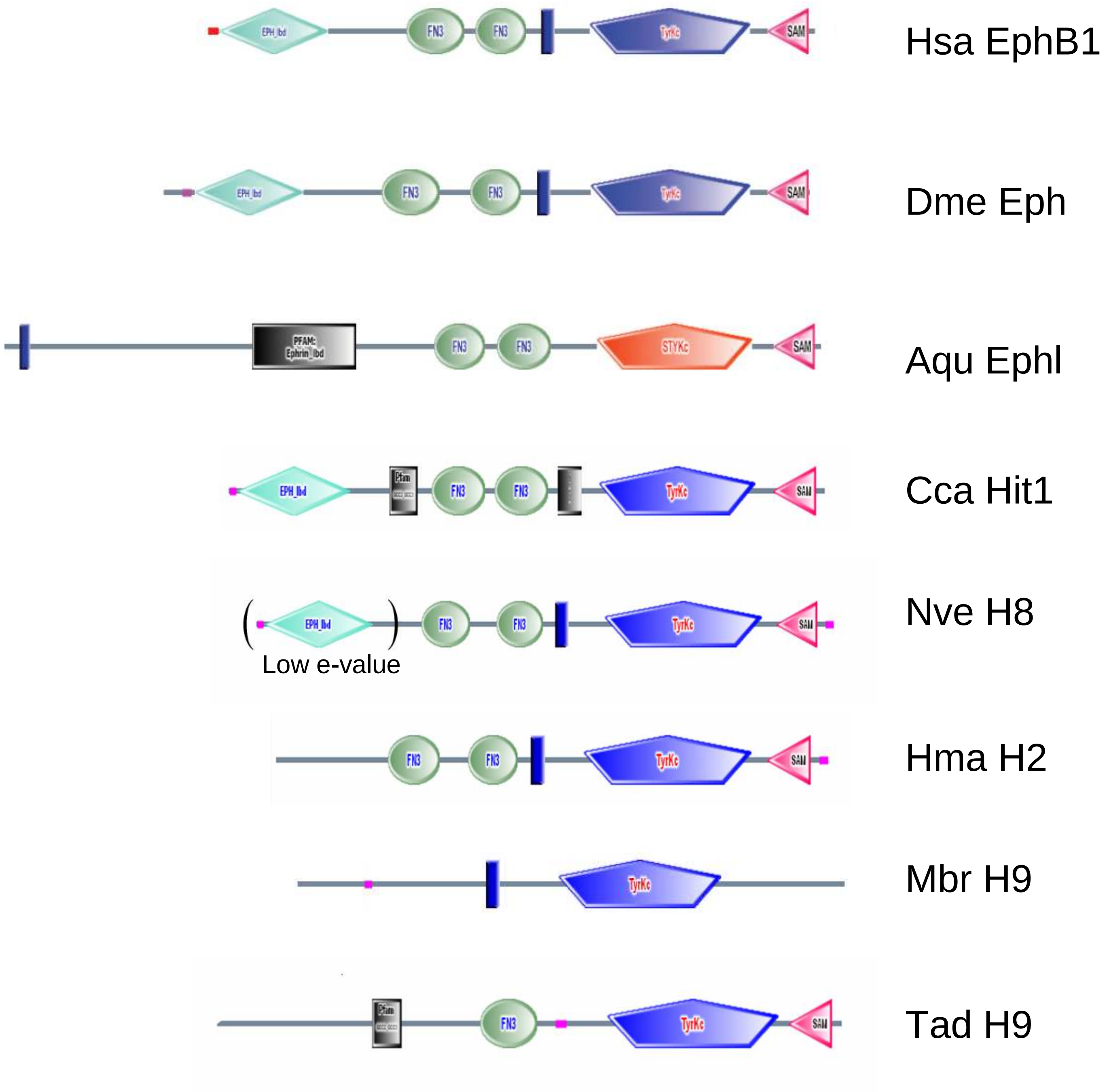
EPH_lbd
FN3
FN3
TyrKc
SAM
Hsa EphB1
EPH_lbd
FN3
FN3
TyrKc
SAM
Dme Eph
PFAM: Ephrin_lbd
FN3
FN3
STYKc
SAM
Aqu Ephl
EPH_lbd
Pfam
FN3
FN3
TyrKc
SAM
Cca Hit1
EPH_lbd
Low e-value
FN3
FN3
TyrKc
SAM
Nve H8
FN3
FN3
TyrKc
SAM
Hma H2
TyrKc
Mbr H9
Pfam
FN3
TyrKc
SAM
Tad H9

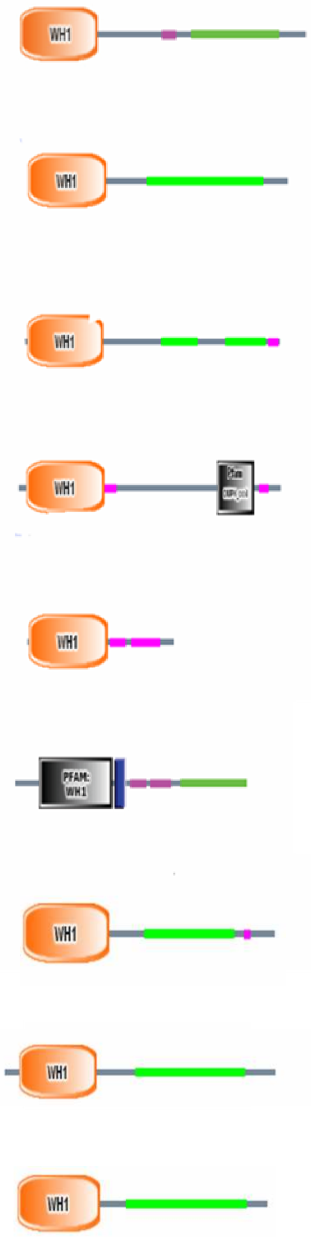

Dme Homer

Hsa Homer1 and Homer2

Hsa Homer3

Nve J1

Hma J1

Aqu Homer

Tad J1

Mbr J1

Cca J1

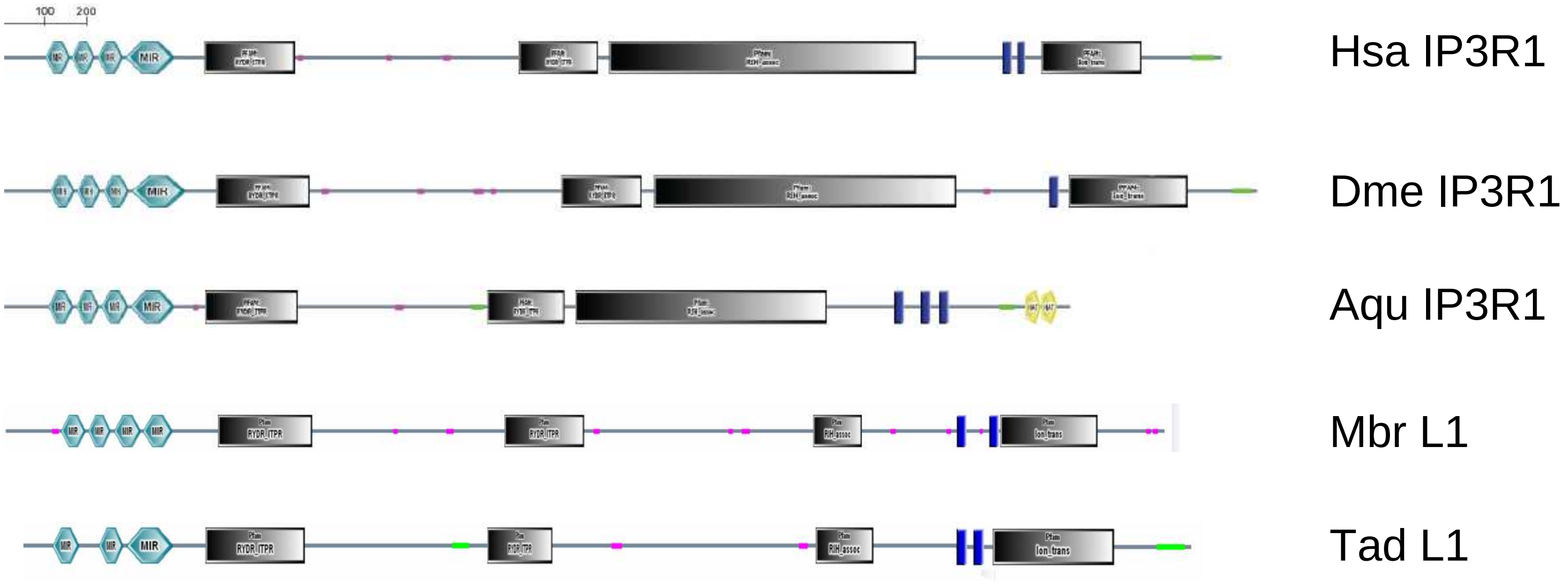

100
200
MIR
Hsa IP3R1
Dme IP3R1
Aqu IP3R1
Mbr L1
Tad L1

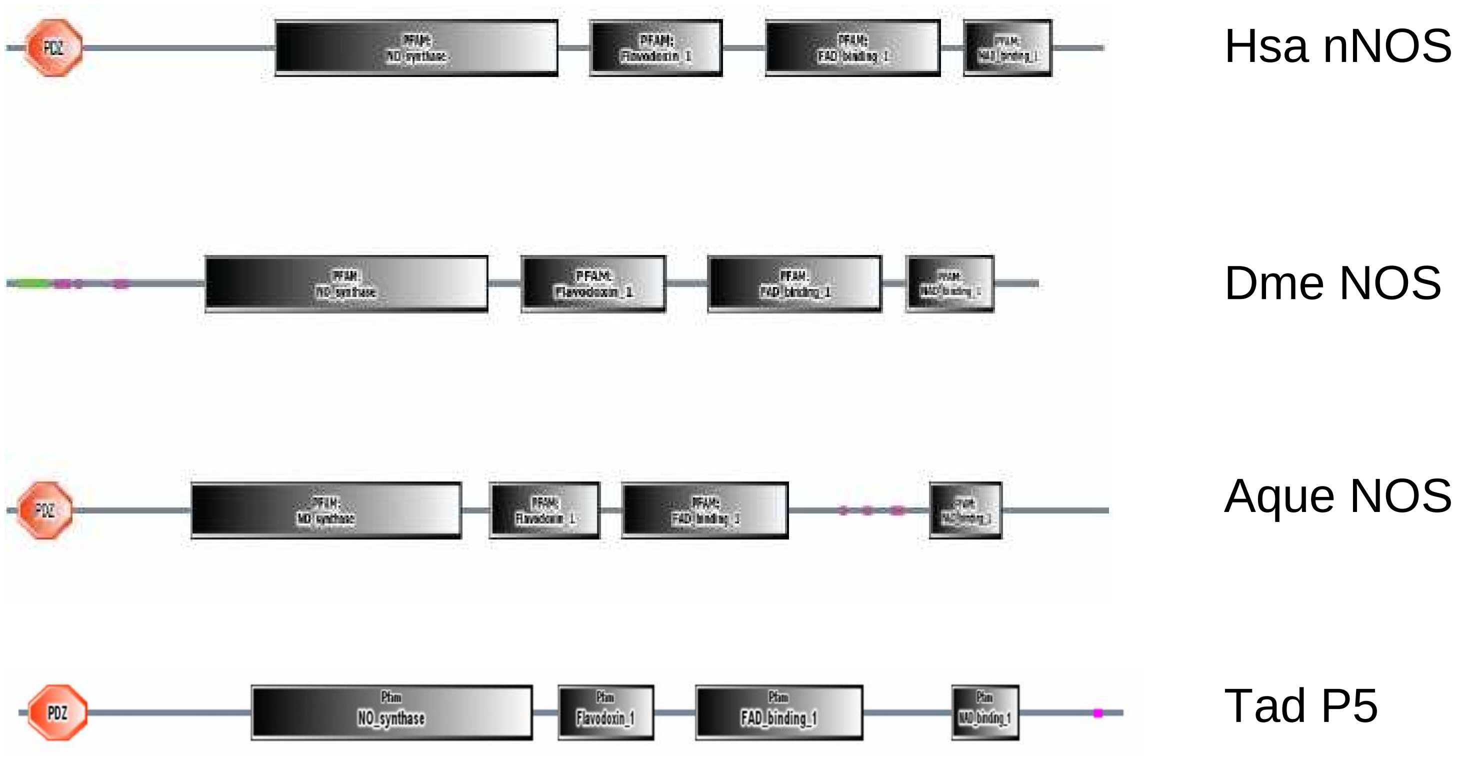

PDZ
PFAM: NO_synthase
PFAM: Flavodoxin_1
PFAM: FAD_binding_1
Hsa nNOS
Dme NOS
Aque NOS
Pfam NO_synthase
Pfam Flavodoxin_1
Pfam FAD_binding_1
Tad P5

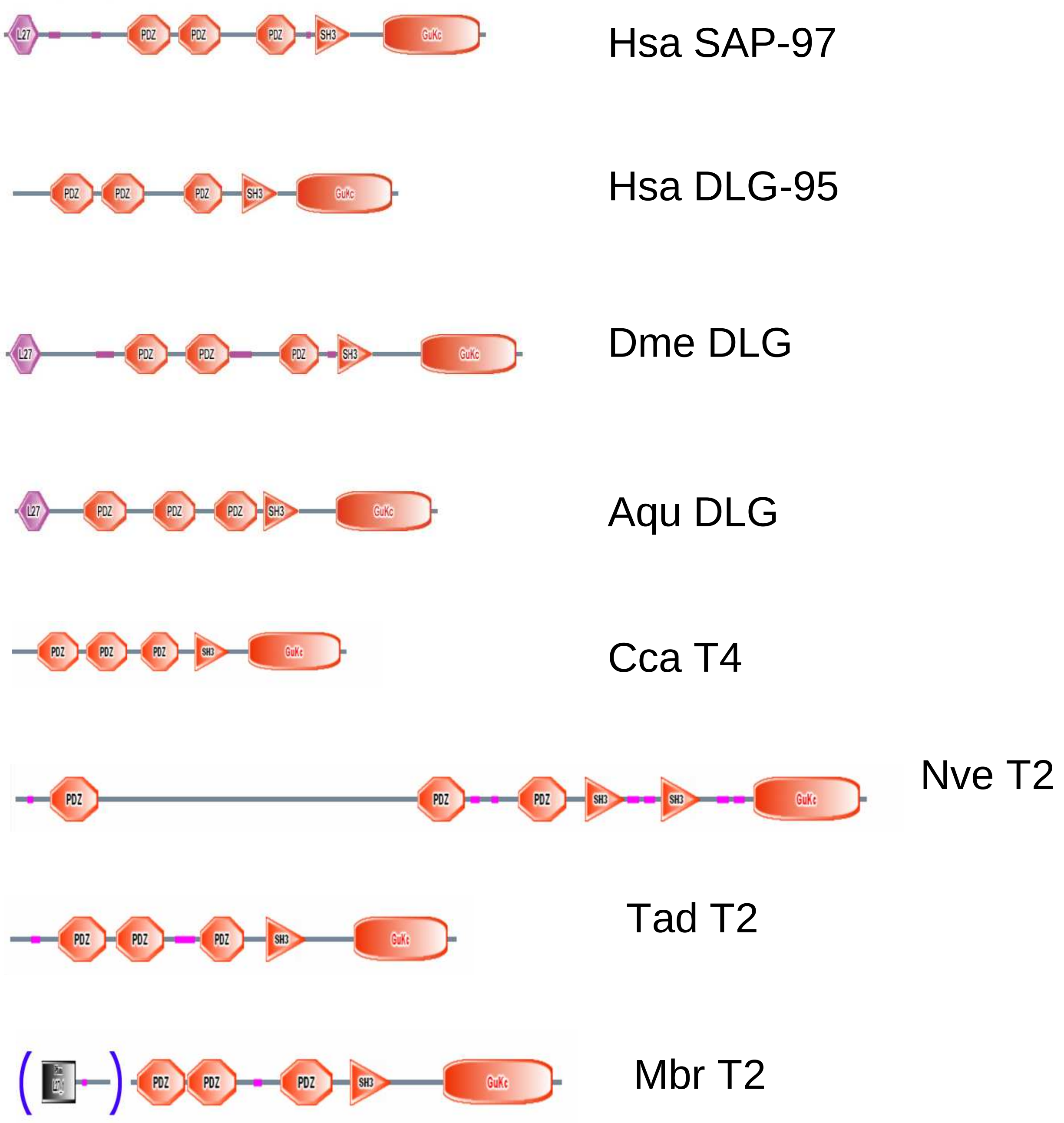

L27 on the same scafflod, in a different reading frame

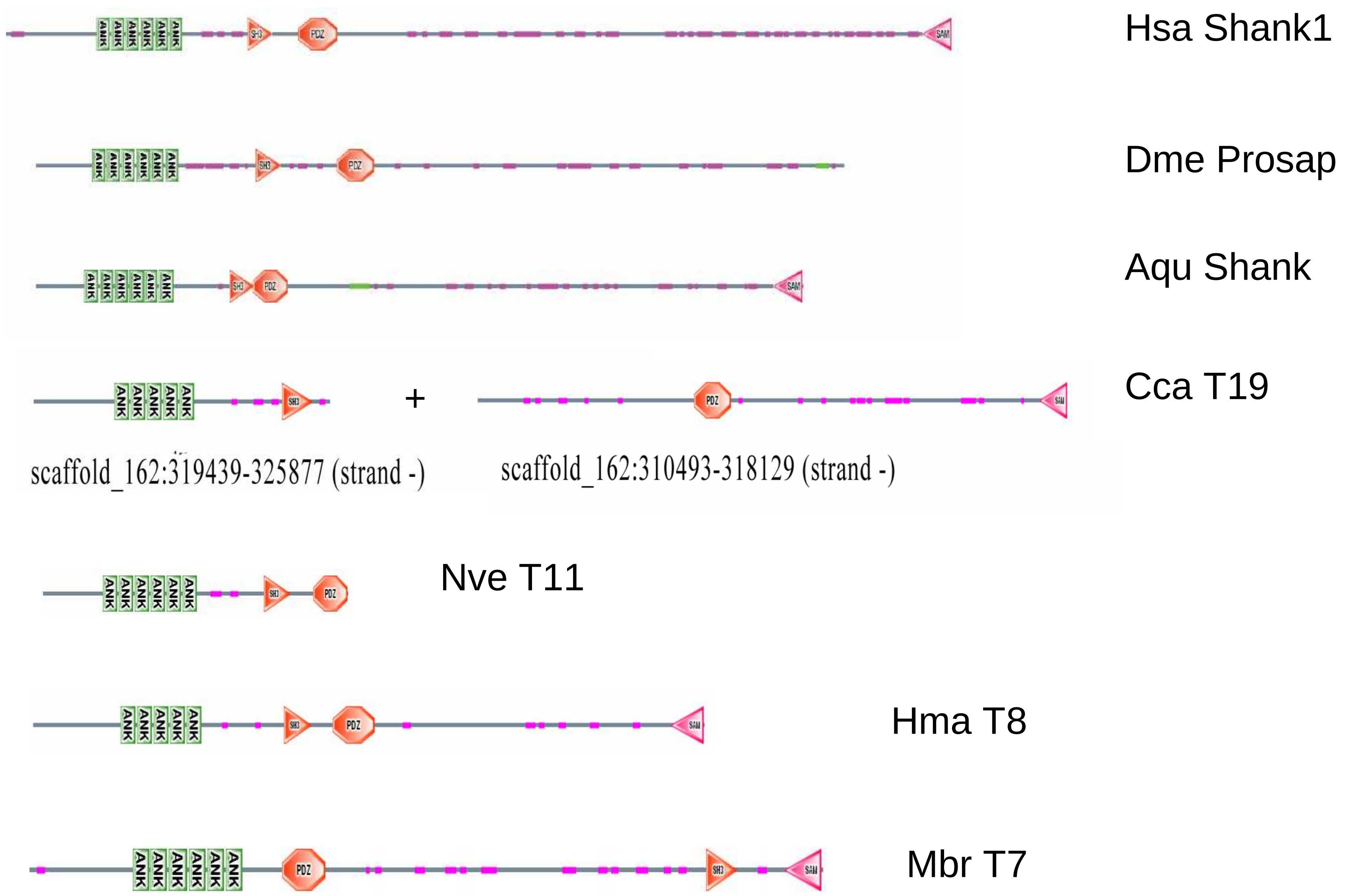
Hsa Shank1
Dme Prosap
Aqu Shank
Cca T19
+
scaffold_162:319439-325877 (strand -)
scaffold_162:310493-318129 (strand -)
Nve T11
Hma T8
Mbr T7
ANK
SH3
PDZ
SAM

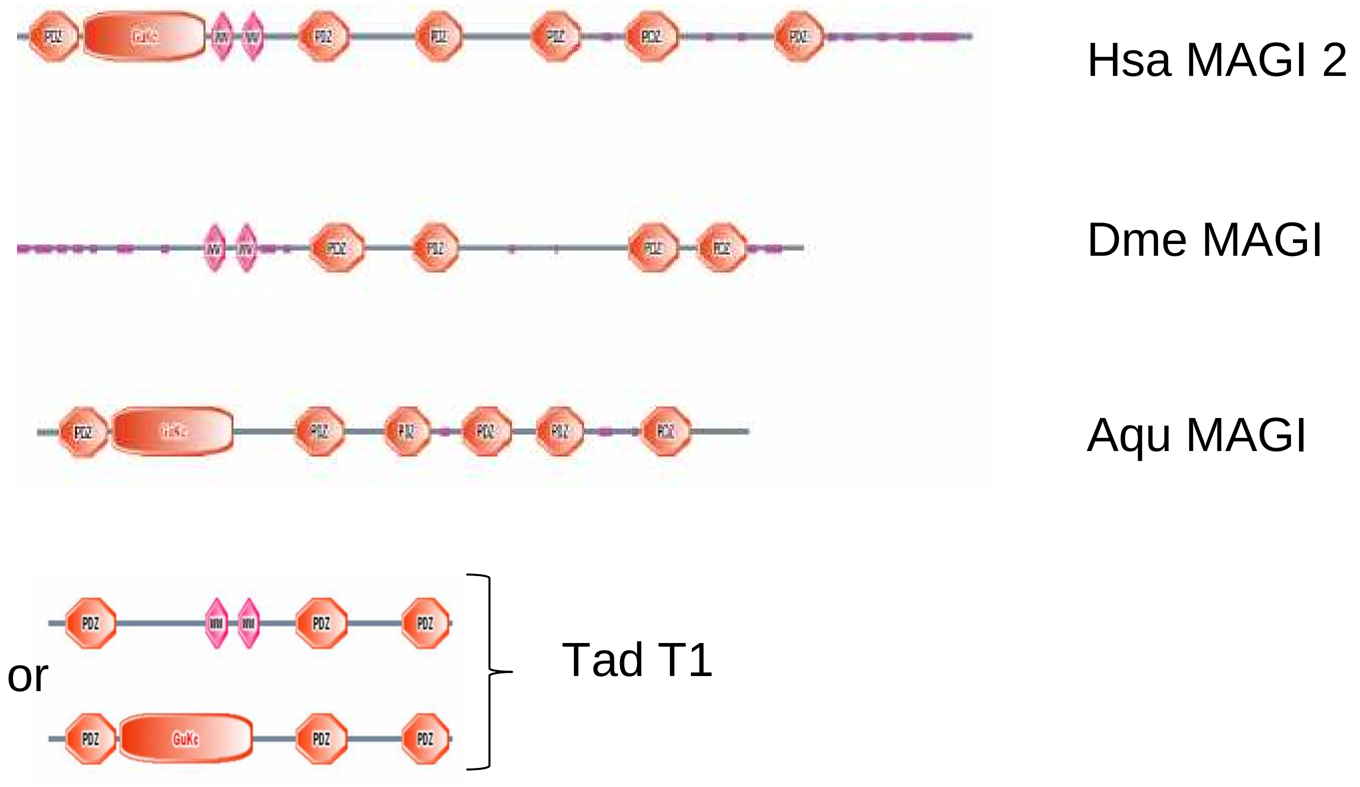
Hsa MAGI 2
Dme MAGI
Aqu MAGI
or
Tad T1
PDZ
GuKc
WW

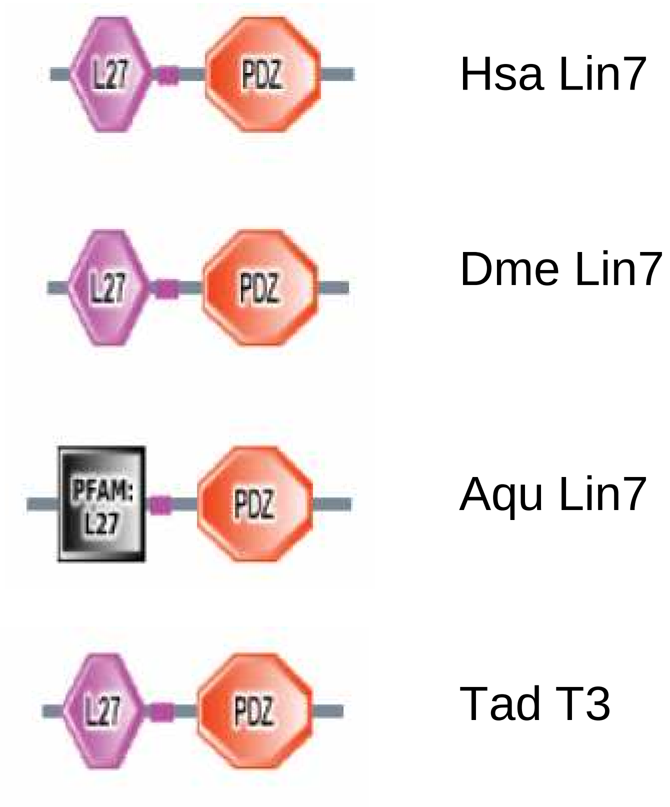
L27
PDZ
Hsa Lin7
L27
PDZ
Dme Lin7
PFAM:
L27
PDZ
Aqu Lin7
L27
PDZ
Tad T3

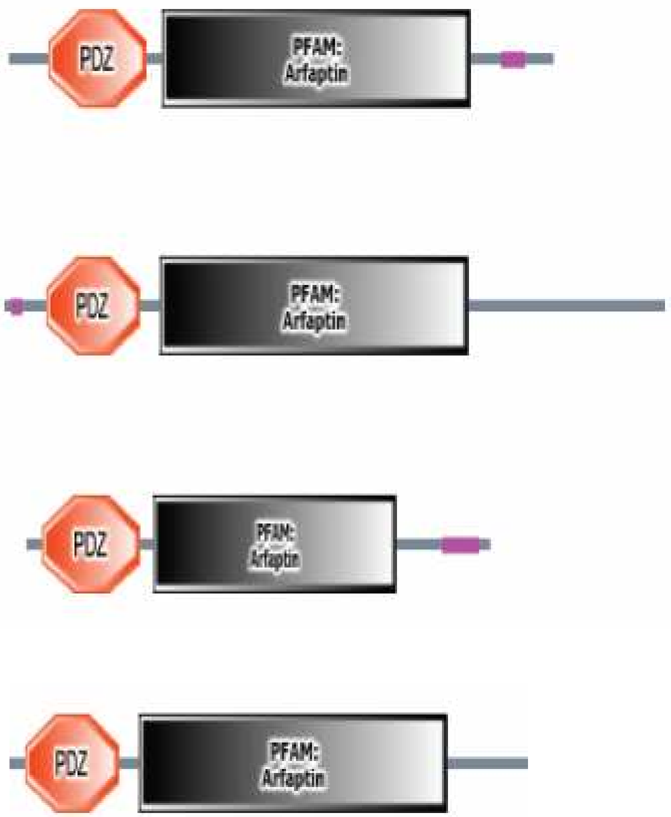

Hsa PICK1

Dme PICK1

Aqu PICK1

Tad T12

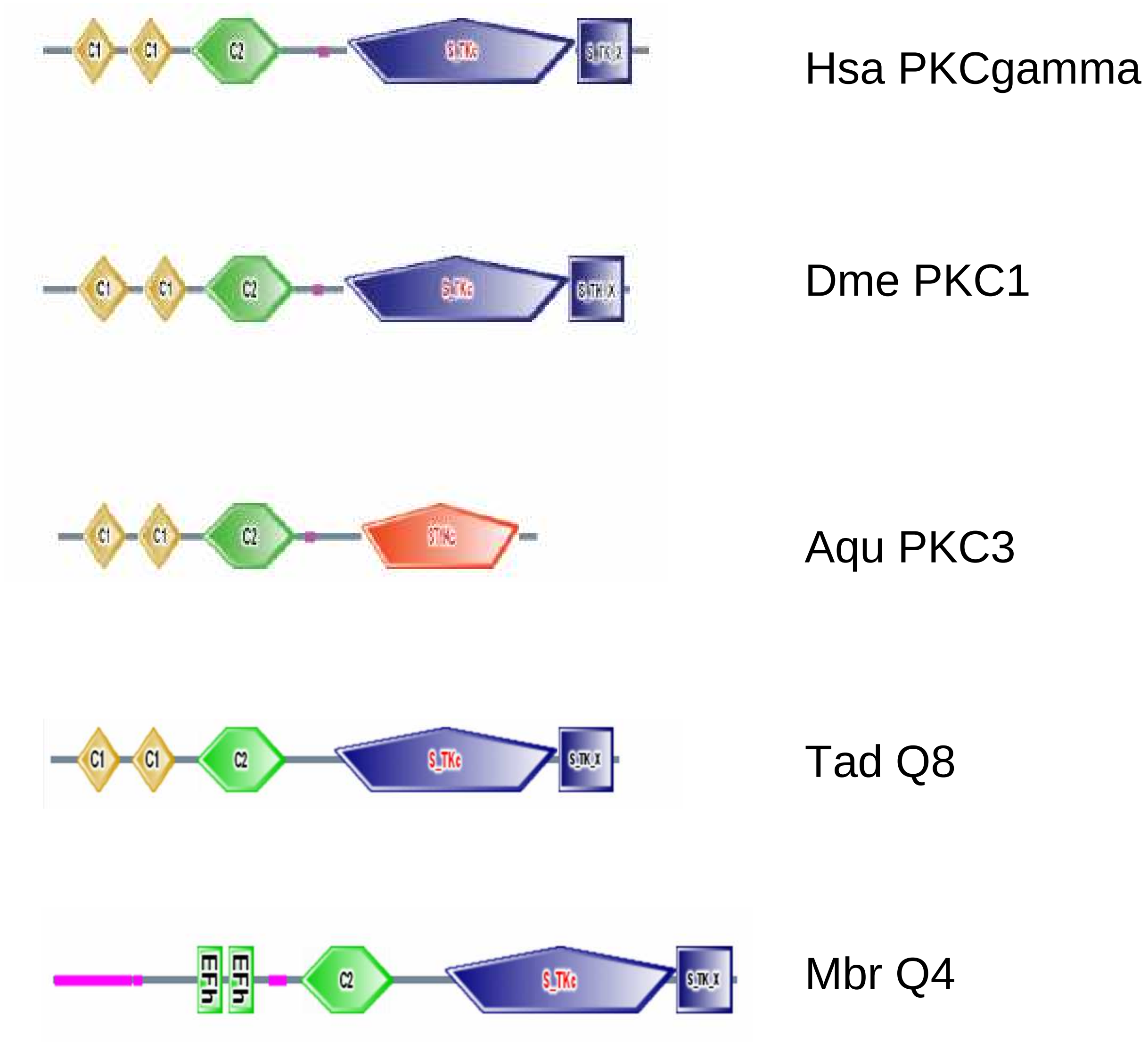

Hsa PKCgamma
Dme PKC1
Aqu PKC3
Tad Q8
Mbr Q4
C1
C2
S_TKc
S_TK_X
EFh

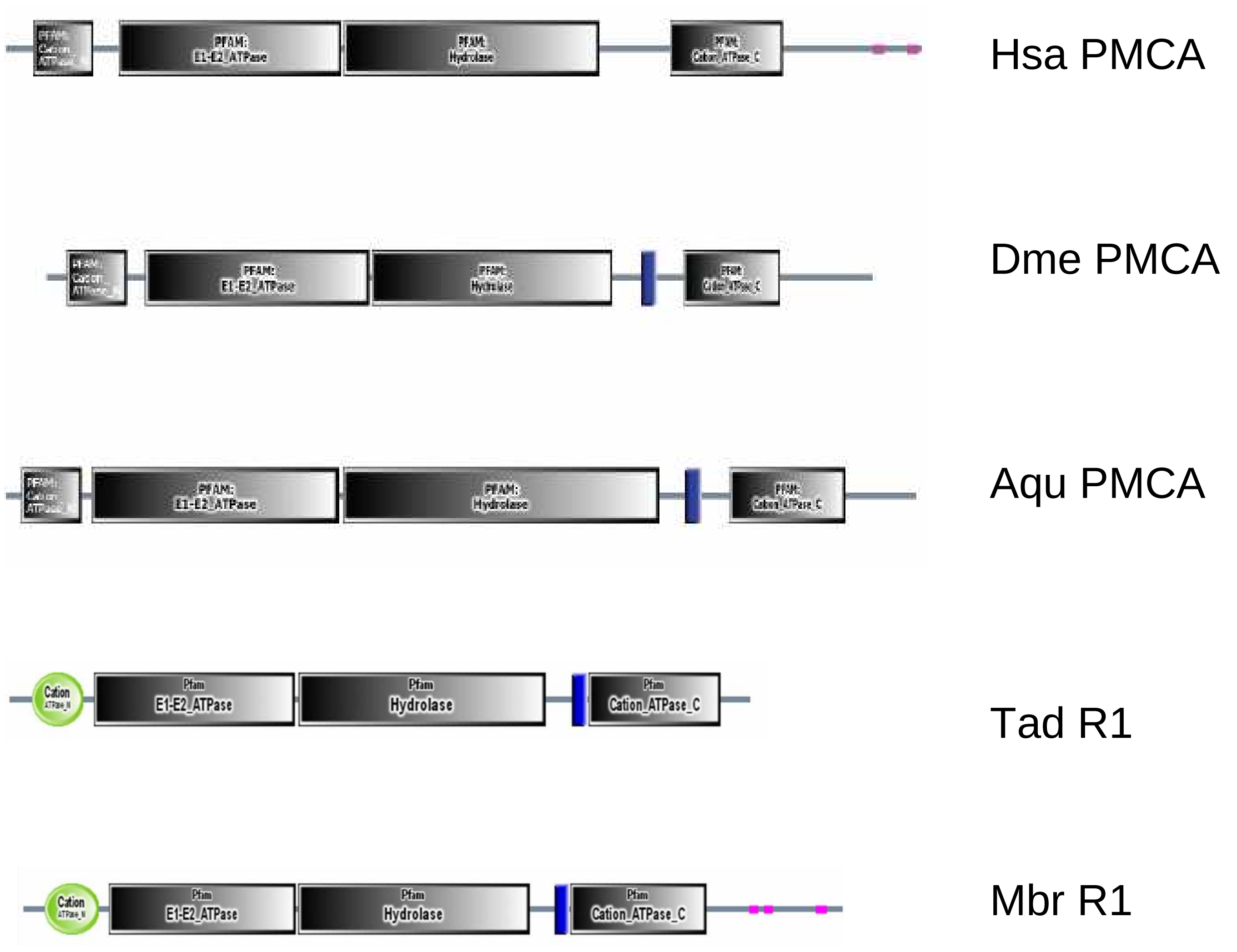

Hsa PMCA
Dme PMCA
Aqu PMCA
Tad R1
Mbr R1
Pfam
E1-E2_ATPase
Hydrolase
Cation_ATPase_C
Cation

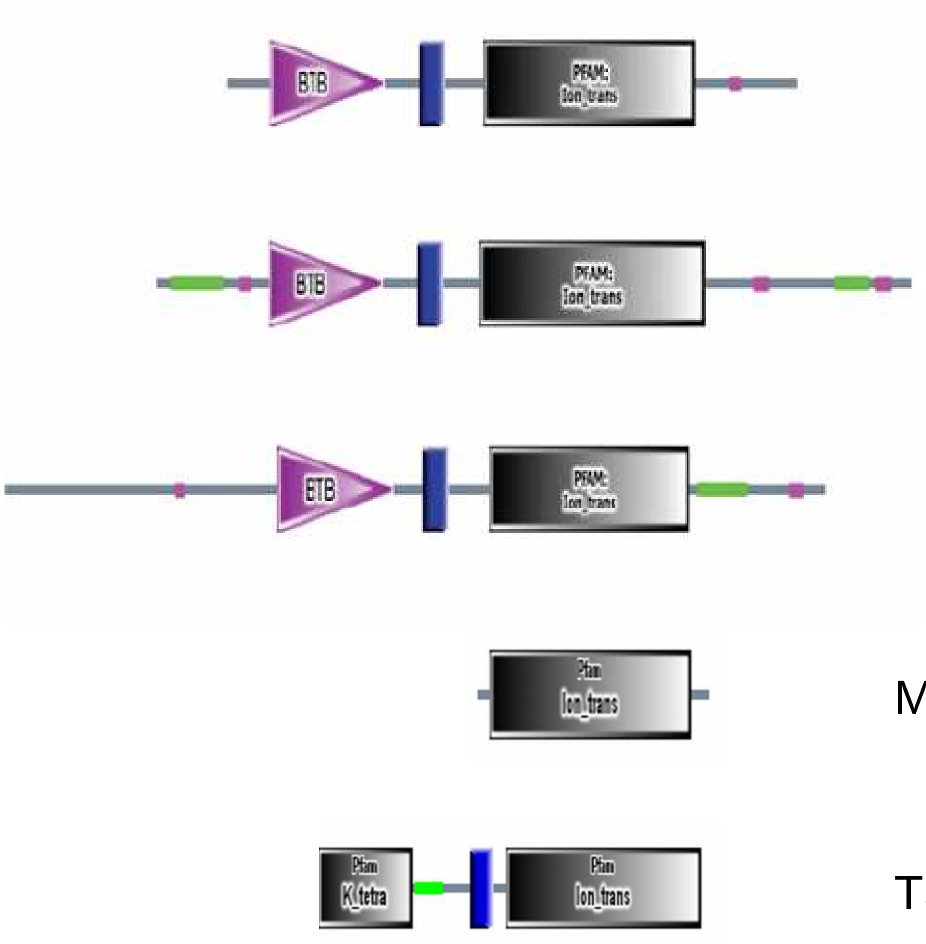

Hsa Kv11

Dme Kv1

Nve Kv 1 like

Mbr S1

Tad S1

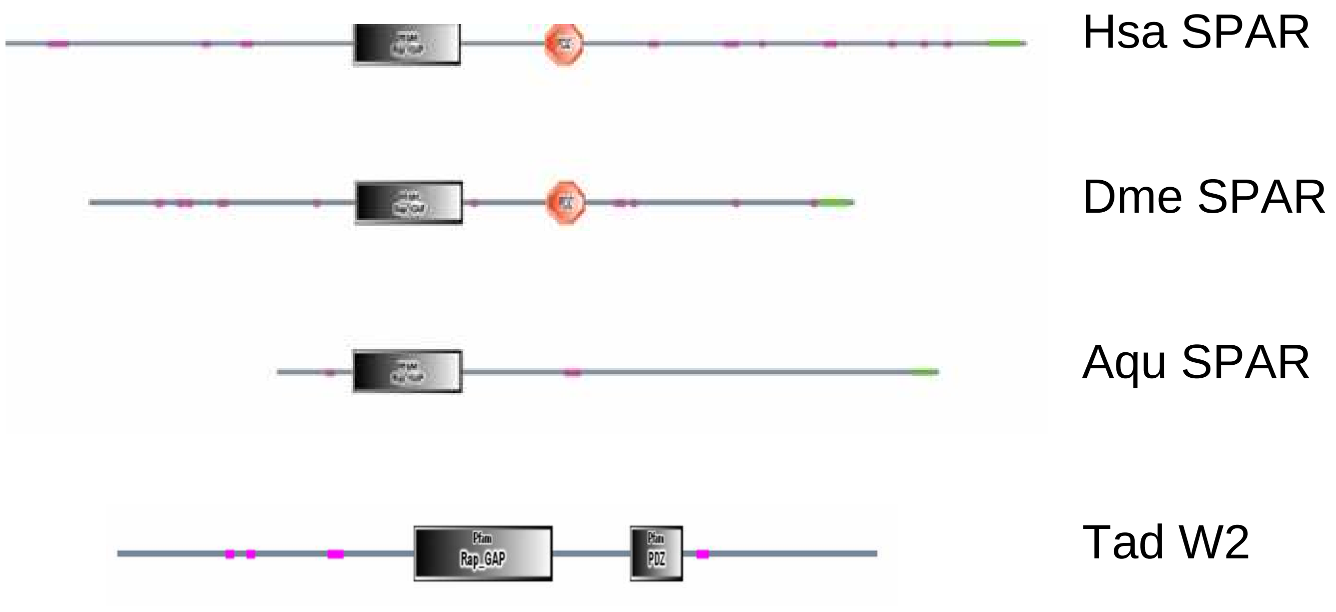
Hsa SPAR
Dme SPAR
Aqu SPAR
Pfam
Rap_GAP
Pfam
PDZ
Tad W2
