# Supplementary figures and images for: The backbone of the post-synaptic density originated in a unicellular ancestor of choanoflagellates and metazoans
Source: BMC Evol Biol. 2010 Feb 3;10:34. doi: 10.1186/1471-2148-10-34 (PMC2824662; doi:10.1186/1471-2148-10-34)

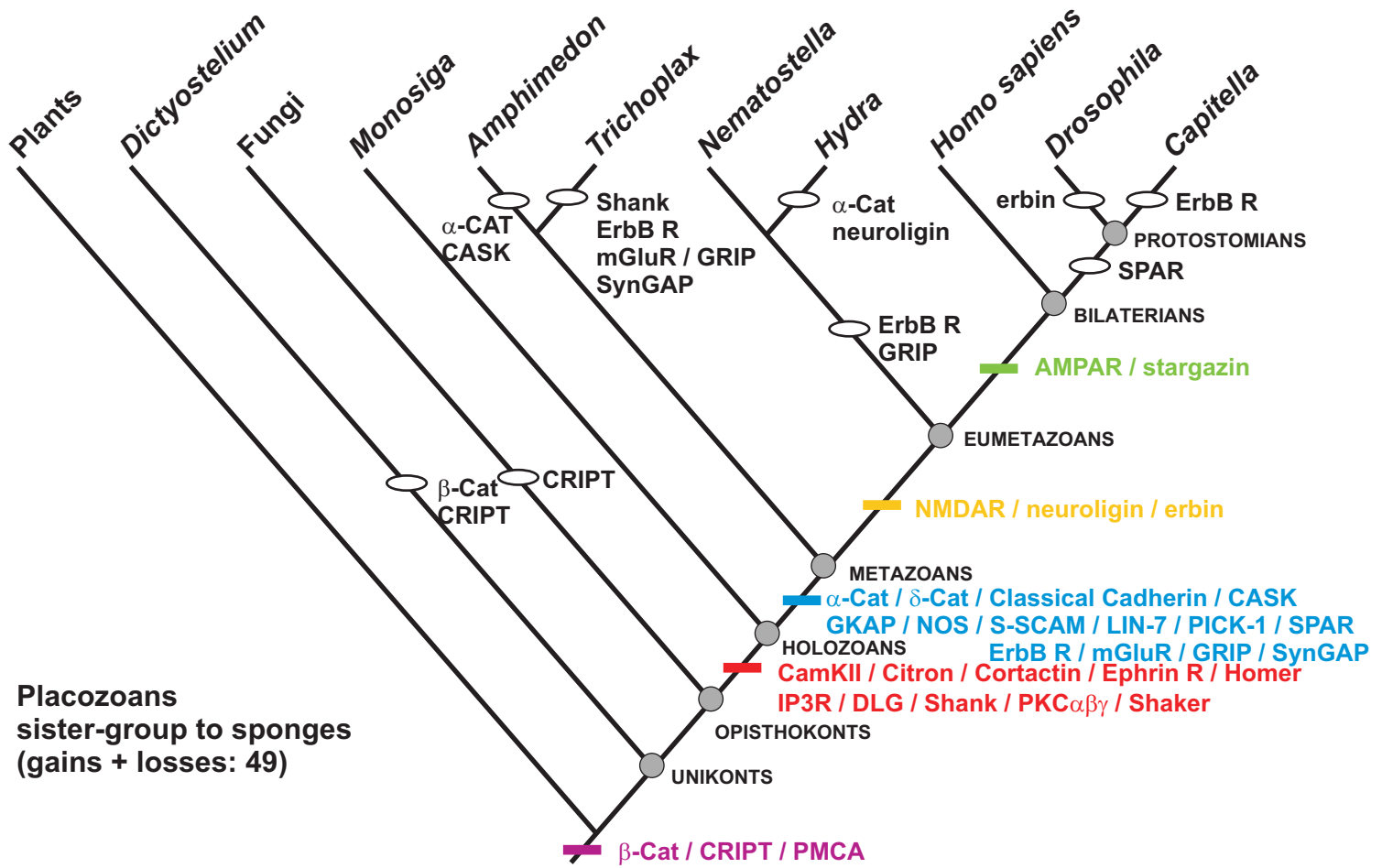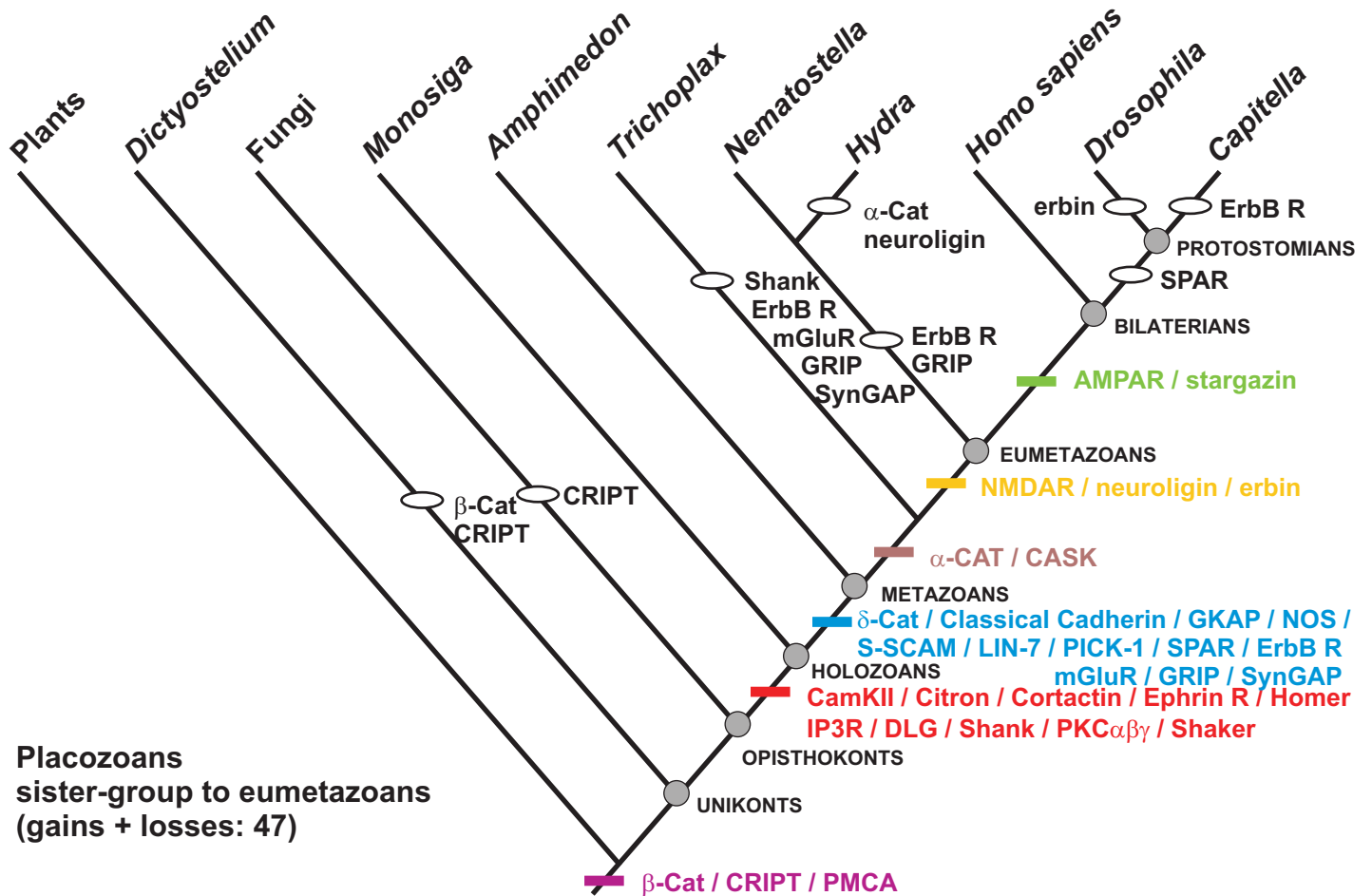

Supplement: Additional file 7 — Gains (coloured dashes) and losses (ellipses) of post-synaptic proteins reconstructed with placozoans as the sister-group to sponges, and with placozoans as the sister-group to eumetazoans. The number of gains + losses is indicated for each tree. [file 1471-2148-10-34-S7.PDF]
