# Supplementary material for: The backbone of the post-synaptic density originated in a unicellular ancestor of choanoflagellates and metazoans
Source: BMC Evol Biol. 2010 Feb 3;10:34. doi: 10.1186/1471-2148-10-34 (PMC2824662; doi:10.1186/1471-2148-10-34)
Supplement: Additional file 8 — Comparison of intron positions in the Shank proteins of metazoans and Monosiga. Overview of intron distribution along the whole protein sequences (A) and detailed view of intron position on the alignment of the N-terminal region upstream of the Ankyrin repeats (B). [file 1471-2148-10-34-S8.DOC]

**A. Distribution of intron positions and frames in the Shank orthologues from human (Hsa), fly (Dme), annelid (Cca), sea anemone (Nve) and choanoflagellate (Mbr):**


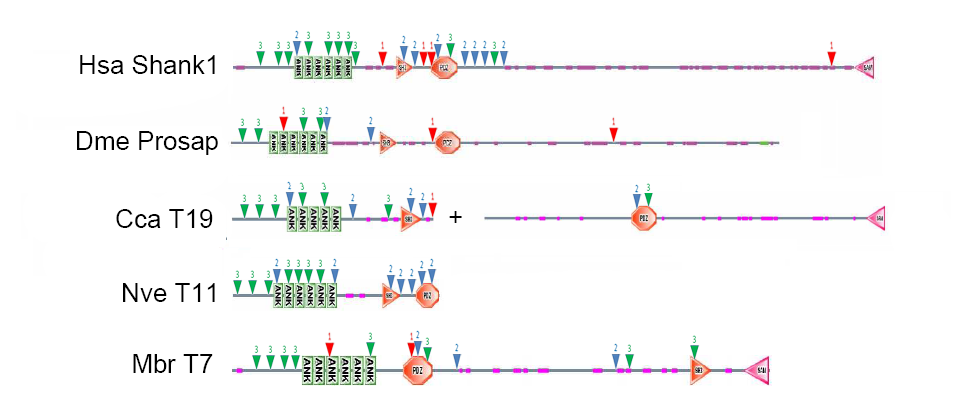


Intron frame indicated by a colour code:


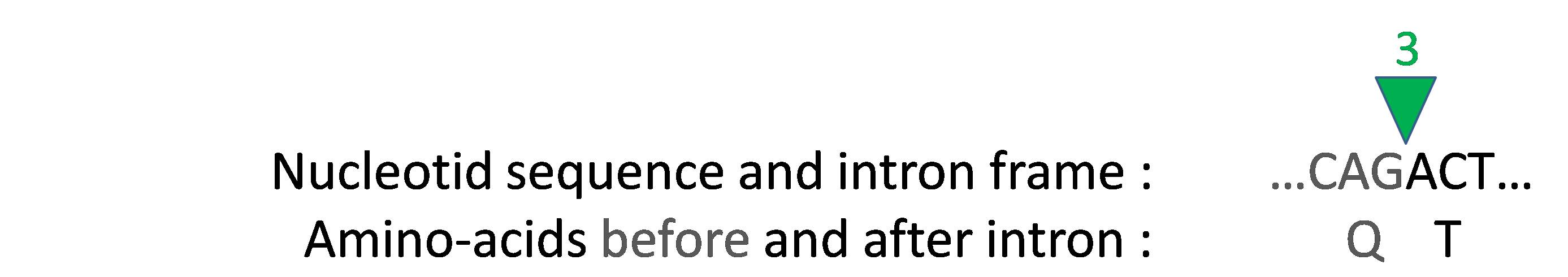

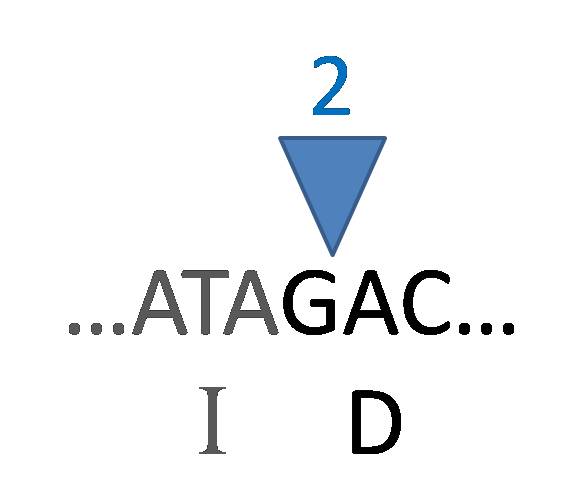

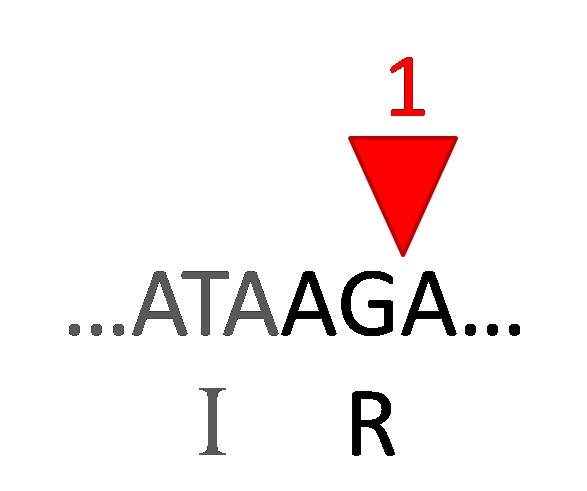


**B. Alignment of the N-terminal** **region (upstream of the Ankyrin repeats):**

Intron frame indicated by colour code as above.

....|....| ....|....| ....|....| ....|....| ....|....|

10 20 30 40 50

**HsaShank1**  MTHSPATSED EERHSASECP EGGSESDSSP DGPGRGPRGT RGQGSGAPGS

**Cca T19**  ---------- ---------- ---------- ---------- ----------

**Nve T11**  ---------- ---------- ---------- ---------- ----------

**DmeProsap**  ---------- ---------- ---------- ---------- -MSGSGA---

**Mbr T7**  ---------- ---------- ---------- -----MAQPQ TSSATGSPMR

....|....| ....|....| ....|....| ....|....| ....|....|

60 70 80 90 100


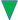


**HsaShank1**  LASVRGLQGR SMSVPDDAHF SMMVFRIGIP DLHQTKCLRF NPDATIWTAK

**Cca T19**  ---------- ---------- ---------- -----KCLQF SQEDSVWTAK

**Nve T11**  ---------- ---------- ---------- -----KLFLF DDQERIWLAK

**DmeProsap**  FDDEPPPE-- -------PRD GWLLVRIHVP ELNVYKCLQF PSERLVWDVK

**Mbr T7**  LADAIPQASA SAVAAALTND DTILCRVSIP EQELQKCLVF DKTETVWTAK

....|....| ....|....| ....|....| ....|....| ....|....|

110 120 130 140 150

**HsaShank1**  QQVLCALSES LQDVLNYGLF QPATSGRDAN FLEEERLLRE YPQSFEKGVP


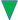


**Cca T19**  QRVLSTLAKD LRDGLNYGLY CPPINGKAGK FLDEERPLKD YP--LPGPIG


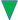


**Nve T11**  LQVISELAKG VKDALNYGFY EPPCNGRSGK FLDEERTFRE YPQRVKP--A


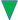


**DmeProsap**  QQVLASLPKE LKESFNYGLF APPANGKAGK FLDEERRLGD YP--FNGPVG


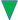


**Mbr T7**  QLVLNKLAKD IPGNINYGLY LPPANGRAGK YLSEERLLGD YG--LAGPVS

....|....| ....|....| ....|....| ....|....| ....|....|

160 170 180 190 200


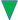

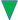


**HsaShank1**  YLEFRYKTRV YKQTNLDEKQ LAKLHTKTGL KKFLEYVQLG TSDKVARLLD


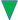

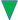


**Cca T19**  FLEFKYKRRV YKLMQINPKK LKQLHTKANL KLFMEMIRRR EVEKILKTVN


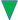

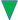


**Nve T11**  QLEFKYKRRV YKSLQYDAKA LQKVHTKGNL KKLLDAVSLG SIPQVTKLTD


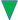


**DmeProsap**  YLELKYKRRV YKMLTLDERQ LKALHTRANL RRFLECINGG HVEKIAKMCA


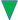

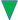


**Mbr T7**  ELQFKLKRRI YLSLPTTSKS LAKLHSRANV RKFFDAVVKH NYERVTNMLV

....|....| ..

210

.......... ..

**HsaShank1**  KGLDPNYHDS DS

**Cca T19**  KGLDPNFHDH E-

**Nve T11**  KGLDPNFQED K-

**DmeProsap**  KGLDPNFHCS E-

**Mbr T7**  KGIDPNMVDD E-
